# Organocatalytic asymmetric Michael addition of unprotected 3-substituted oxindoles to 1,4-naphthoquinone

**DOI:** 10.3762/bjoc.8.157

**Published:** 2012-08-23

**Authors:** Jin-Sheng Yu, Feng Zhou, Yun-Lin Liu, Jian Zhou

**Affiliations:** 1 Shanghai Key Laboratory of Green Chemistry and Chemical Processes, Department of Chemistry, East China Normal University, 3663 N. Zhongshan Road, Shanghai, 200062, P. R. China

**Keywords:** 3,3-disubstituted oxindoles, Michael addition, organocatalysis, quaternary stereogenic center, unprotected 3-substituted oxindoles

## Abstract

We reported the first example of organocatalytic Michael addition of unprotected 3-prochiral oxindoles **1** to 1,4-naphthoquinone. Quinidine derivative (DHQD)_2_PYR was found to be able to catalyze this reaction in up to 83% ee, with moderate to excellent yields. This method could be used for the synthesis of enantioenriched 3,3-diaryloxindoles, and the catalytic synthesis of which was unprecedented.

## Introduction

The catalytic asymmetric synthesis of 3,3-disubstituted oxindoles has recently received great attention because of the wide occurrence of this structural motif in natural products and pharmaceutically active compounds [1–3]. In addition, structure–activity relationship studies have revealed that the absolute configuration and the substituent of the C3 position of oxindole greatly influenced the biological activities [4]. Accordingly, the development of efficient synthetic methods to enable the synthesis of 3,3-disubstituted oxindoles in great structural diversity is of current interest, and much progress had been made in the catalytic enantioselective synthesis of 3-hydroxyoxindoles [5–10], 3-aminooxindoles [11–15] and 3-quaternary oxindoles [16–20]. Despite achievements, the catalytic asymmetric synthesis of 3,3-diaryloxindoles has not been reported. This is possibly due to the challenge in the construction of such congested quaternary stereogenic centers. Only Sammakia tried the S_N_Ar reaction of unprotected 3-phenyloxindole with chiral electron-deficient 5-halooxazoles, promoted by 1.0 equiv of Cs_2_CO_3_ [21], with ca. 1:1 diastereoselectivity obtained.

In this context, we are interested in the catalytic economical asymmetric diverse synthesis of 3,3-disubstituted oxindoles, using cheap and easily available starting materials and simple chiral catalysts to facilitate biological evaluation. We have developed the catalytic asymmetric addition of acrolein, allyltrimethylsilane or difluoroenoxysilanes to isatins to furnish differently substituted enantioenriched 3-hydroxyoxindoles [22–24]. For the synthesis of chiral 3-aminooxindoles, we developed the first example of catalytic asymmetric addition of nucleophiles to isatin-derived ketoimines using TMSCN [25] and the amination of unprotected 3-prochiral oxindoles using di-*tert*-butyl azodicarboxylate [26–27]. To construct the C3 quaternary stereogenic carbon center, we have designed a novel cinchona alkaloid-based phosphoramide bifunctional catalyst to realize a highly enantioselective Michael addition of both unprotected 3-alkyl- and 3-aryloxindoles to nitroolefins [28]. Based on these results, together with our efforts in the synthesis of unsymmetric 3,3-diaryloxindoles [29], we try to develop a catalytic asymmetric method to enantioenriched 3,3-diaryloxindoles.

In 2007, Jørgensen and coworkers pioneered the organocatalytic asymmetric addition reactions to quinones [30–31] which turned out to be a powerful strategy for the α-arylation of β-ketoesters and aldehydes. Inspired by their work, we anticipated that the catalytic asymmetric addition of 3-aryloxindoles to quinones would possibly install a hydroquinone moiety at the C3 position of oxindole to furnish the desired chiral 3,3-diaryloxindoles. It also came to our attention that, while the addition of 3-prochiral oxindole to a variety of Michael acceptors had been studied [32–46], the use of quinones as the Michael acceptor had not been realized. Therefore, in this letter we are going to report our initial results about the catalytic asymmetric Michael addition of unprotected 3-prochiral oxindoles to 1,4-naphthoquinone.

## Results and Discussion

**Figure 1:**
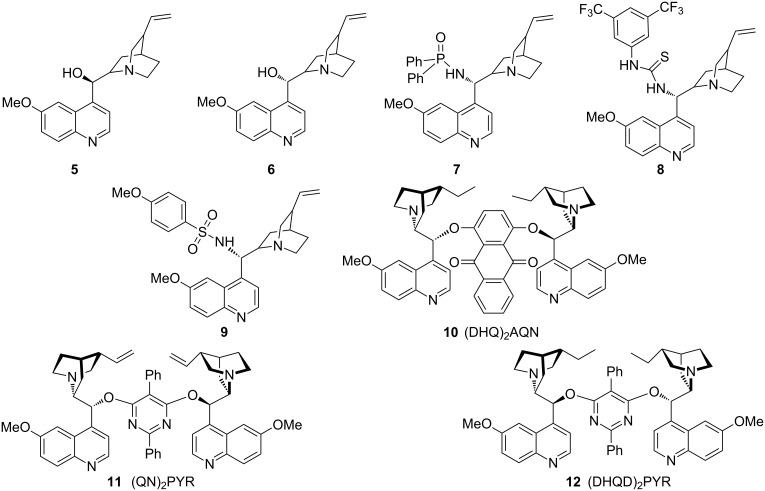
Cinchona alkaloid-derived catalysts screened for condition optimization (Table 1).

We began the reaction development by the evaluation of different chiral catalysts derived from cinchona alkaloids in the reaction of 3-phenyloxindole **1a** and 1,4-naphthoquinone (**2a**), with ethyl acetate (EtOAc) as the solvent at 0 °C (Table 1, Figure 1). A variety of bifunctional cinchona alkaloid-derived catalysts **5**–**9** were first tried, aiming to facilitate the reaction by the dual activation of both reaction partners, with H-bonding donor moiety of the catalyst to activate quinone **2a** and the tertiary amine to deprotonatively activate oxindole **1**. The reaction generally proceeded slowly, and only the oxidation product, 1,4-naphthoquinone derivative **3a**, was obtained in moderate yield after five days. No hydroquinone product **4** was detected by TLC and NMR analysis of the crude reaction mixture. While the simple quinine and quinidine as catalysts could deliver product **3a** in 59% ee (Table 1, entry 2), all other bifunctional catalysts turned out to be much less enantioselective (Table 1, entries 3–5). However, the dinuclear Brønsted base catalysts **10**–**12** could achieve higher ee for the desired product **3a** with comparable yields (Table 1, entries 6–8). When the hydrogenated catalyst **12** was used, 77% ee for product **3a** was obtained with 51% yield (Table 1, entry 8). In light of this, we used catalyst **12** for the following screenings.

**Table 1:** Condition optimization for the reaction of **1a** and **2a**.

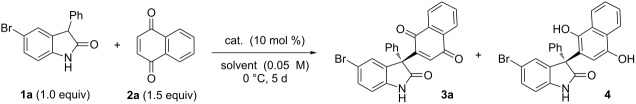					

Entry^a^	Cat.	Solvent	Additive	Yield of **3a** (%)^b^	ee (%)^c^

1	**5**	EtOAc	–	52	43^d^
2	**6**	EtOAc	–	61	59
3	**7**	EtOAc	–	40	14
4	**8**	EtOAc	–	34	4
5	**9**	EtOAc	–	14	15
6	**10**	EtOAc	–	60	64^d^
7	**11**	EtOAc	–	64	73^d^
8	**12**	EtOAc	–	51	77
9	**12**	THF	–	50	64
10	**12**	Acetone	–	61	64
11	**12**	CH_3_CN	–	32	47
12	**12**	DCM	–	21	36
13	**12**	Toluene	–	31	73
14	**12**	EtOAc	MS 4Å	43	80
15	**12**	EtOAc	MS 5Å	29	70
16	**12**	EtOAc	H_2_O (5.0 equiv)	50	78
17	**12**	EtOAc	H_2_O (10.0 equiv)	50	78
18	**12**	EtOAc	PhCO_2_H^e^	33	76
19	**12**	EtOAc	(*S*)-BINOL^e^	36	40
20	**12**	EtOAc	(*R*)-BINOL^e^	43	77
21	**12**	EtOAc	LiCl^e^	65	4

^a^Reactions were run on a 0.10 mmol scale. ^b^Isolated yield. ^c^Determined by chiral HPLC analysis. ^d^Opposite enantiomer. ^e^10 mol % used.

We further examined the solvent effects, and found that EtOAc turned out to be the most suitable solvent which afforded product **3a** in highest ee (Table 1, entries 8–13). Since the reactivity was unsatisfactory, we further tried the use of some additives to improve the reaction rate. The use of MS 4Å could improve the enantioselectivity to 80%, but decreased the yield from 51% to 43% (Table 1, entry 8 versus 14). The use of MS 5Å had a negative effect on both the reactivity and the enantioselectivity (Table 1, entry 15). Water had no obvious effect on the reaction outcome (Table 1, entries 16 and 17). The addition of acids led to diminished yield and enantioselectivity (Table 1, entries 18–20).

Based on these screenings, we determined to examine the substrate scope by running the reaction at 0 °C in EtOAc, with 20 mol % of (DHQD)_2_PYR **12** to improve the reactivity. Different substituted 3-prochiral oxindoles were first examined and the results are shown in Table 2. An electron-withdrawing substituent at the C5 position of the oxindole had a positive effect on the reactivity and enantioselectivity of the reaction. The corresponding products **3a**–**d** could be obtained in good to excellent yields with up to 81% ee. Without an electron-withdrawing group, products **3e** and **3f** were obtained in diminished yields and enantioselectivities. Different aryl substituents at the C3 position were also investigated, the corresponding products **3g**–**k** were obtained in acceptable yields and up to 83% ee. We also tried if this method could be extended to 3-alkyloxindoles but had to find out that product **3l** was obtained in only moderate enantioselectivity and yield. The absolute configuration of product **3f** was determined to be (*S*) by chemical transformation to the corresponding known compound [47]; all other products were tentatively assigned in analogy (for details, see Supporting Information File 1).

**Table 2:** Substrate scope of unprotected 3-prochiral oxindoles^a–c^.

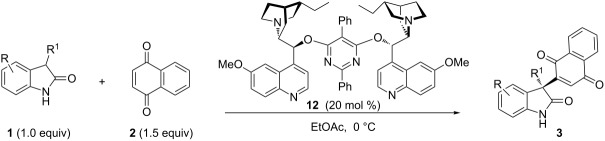			

Product, yield, enantioselectivity and reaction time			

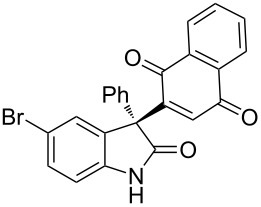 **3a**	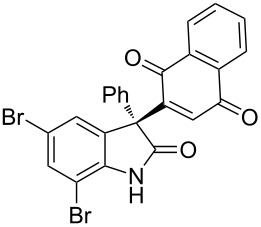 **3b**	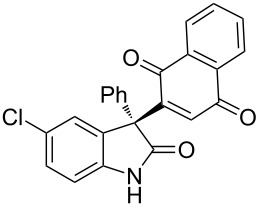 **3c**	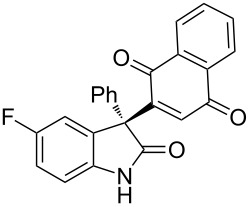 **3d**
70% yield, 79% ee, 5 d	97% yield, 80% ee, 6 d	74% yield, 81% ee, 6 d	71% yield, 79% ee, 8 d
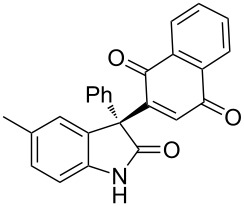 **3e**	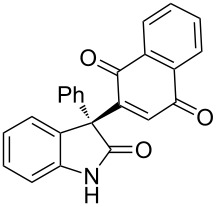 **3f**	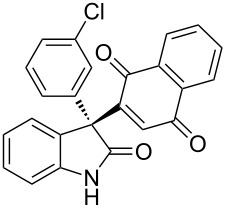 **3g**	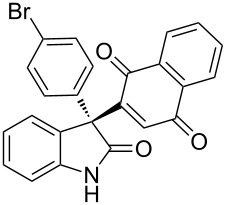 **3h**
46% yield, 69% ee, 9 d	51% yield, 70% ee, 9 d	76% yield, 81% ee, 6 d	45% yield, 54% ee, 8 d
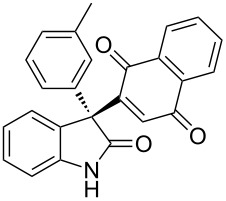 **3i**	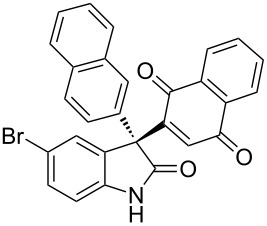 **3j**	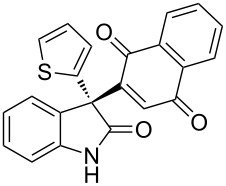 **3k**	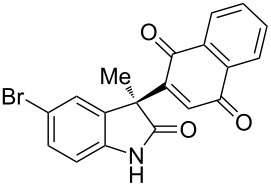 **3l**
55% yield, 83% ee, 6 d	68% yield, 77% ee, 6 d	53% yield, 65% ee, 6 d	58% yield, 19% ee, 8 d

^a^Run on a 0.25 mmol scale. ^b^Isolated yield. ^c^Determined by chiral HPLC analysis.

Other quinones such as 2,6-dichloro-1,4-benzoquinone and 1,4-benzoquinone were also examined, however, none of them could react with 3-phenyloxindole **1a** to give the desired product.

**Scheme 1:**
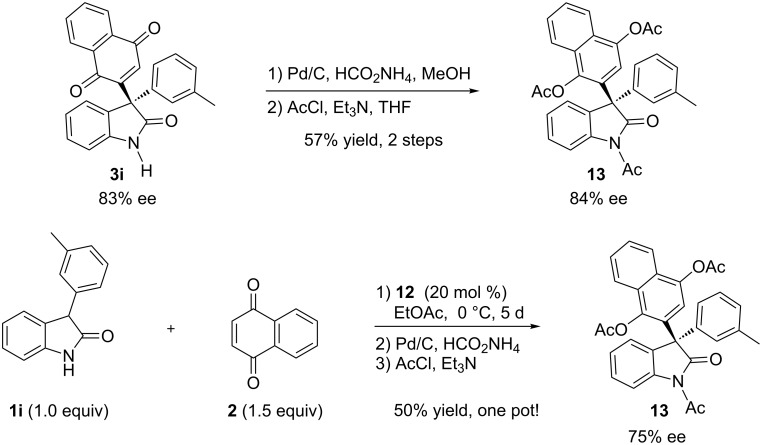
A one-pot synthesis of enantioenriched 3,3-diaryloxindoles.

While the oxidation product **3** was obtained as the only product from the Michael addition, it could be hydrogenated to the corresponding hydroquinone product **4.** The free hydroxy groups were protected to prevent re-oxidation. For example, product **3i** was reduced and converted to the desired 3,3-diaryloxindole **13** in 57% yield without the loss of ee. We further checked if this protocol could be operated as a “one-pot” sequential reaction. After the reaction of **1i** and **2** was run at 0 °C for five days a small amount of oxindole **1i** still remained. Then, the reaction was warmed to room temperature, followed by the addition of Pd/C and ammonium formate. When TLC analysis revealed that the hydrogenation of product **3i** was completed, acetyl chloride and triethylamine were added. The desired product **13** was obtained in 50% yield with 75% ee (Scheme 1). The diminished enantioselectivity was due to the fact that the remaining oxindole **1i** continued to react with **2** at room temperature during the following steps. Even if there is much potential for further improvement in the ee, this sequential reaction represented the first example of catalytic asymmetric synthesis of 3,3-diaryl oxindoles.

## Conclusion

In summary, we have developed the first example of organocatalytic Michael addition of unprotected 3-prochiral oxindoles to 1,4-naphthoquinone [48] with good to high yields and enantioselectivities. This method could be used for the synthesis of enantioenriched 3,3-diaryloxindoles and the catalytic synthesis of which was unprecedented. The development of new chiral catalysts to improve both the reactivity and enantioselectivity of this reaction is now in progress in our lab.

## Supporting Information

File 1 General experimental procedures and compound characterization.
