# Supplementary material for: Organocatalytic asymmetric Michael addition of unprotected 3-substituted oxindoles to 1,4-naphthoquinone
Source: Beilstein J Org Chem. 2012 Aug 23;8:1360–5. doi: 10.3762/bjoc.8.157 (PMC3458762; doi:10.3762/bjoc.8.157)
Supplement: File 1 — General experimental procedures and compound characterization. [file Beilstein_J_Org_Chem-08-1360-s001.pdf]

Supporting Information

for

Organocatalytic asymmetric Michael addition of unprotected 3-substituted oxindoles to 1,4-naphthoquinone

Jin-Sheng Yu, Feng Zhou, Yun-Lin Liu and Jian Zhou*

Address: Shanghai Key Laboratory of Green Chemistry and Chemical Processes, Department of Chemistry, East China Normal University, 3663 N. Zhongshan Road, Shanghai, 200062 (P. R. China)

Email: Jian Zhou* - jzhou@chem.ecnu.edu.cn

* Corresponding author

General experimental procedures and compound characterization

Table of contents

General information 2

Experimental data 3

Determination of absolute configuration 11

Reference 12

^{1}H, ^{13}C, ^{19}F NMR and HPLC spectra.. 13

General information

Reactions were monitored by thin layer chromatography (TLC) using UV light to visualize the course of reaction. Purification of reaction products was carried out by flash chromatography on silica gel. Chemical yields refer to pure isolated substances. The $[\alpha]_D$ was recorded using PolAAr 3005 High Accuracy Polarimeter. Infrared (IR) spectra were obtained using a Bruker tensor 27 infrared spectrometer. ^{1}H and ^{13}C NMR spectra were obtained using a Bruker DPX-400 spectrometer. Chemical shifts are reported in ppm from tetramethylsilane with the solvent resonance as the internal standard. The following abbreviations were used to designate chemical shift multiplicities: s = singlet, d = doublet, t = triplet, q = quartet, h = heptet, m = multiplet, br = broad.

All reactions were carried out in air except noted. Anhydrous ethyl acetate was prepared by distillation over activated calcium sulfate and 5 Å MS prior to use. The chiral catalyst $(DHQD)_2PYR$ and 1,4-naphthoquinone were commercially available. 3-Substituted oxindoles **1** [1] were prepared from the corresponding isatins according to literature reports.

Experimental data

General procedure for the Michael addition of unprotected 3-substituted oxindoles 1 and 1,4-naphthoquinone (2).

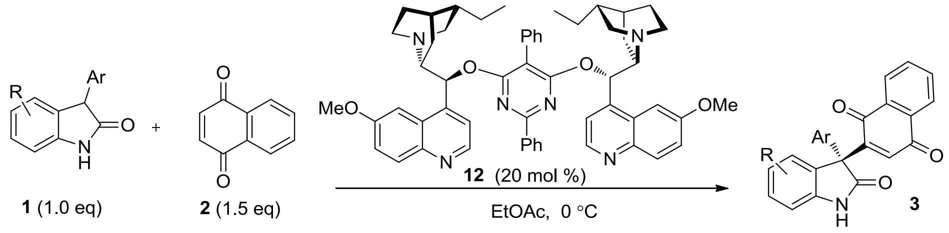

To a 10 mL vial were added catalyst **12** (44.0 mg, 0.05 mmol) and 3-substituted oxindoles **1** (0.25 mmol) followed by 5 mL of anhydrous ethyl acetate. The reaction mixture was stirred vigorously at room temperature until the full dissolution of catalyst **12**. The resulting mixture was stirred at 0 °C for about 20 min before 1,4-naphthoquinone (**2**, 59.3 mg, 0.375 mmol) was added. After completion of the reaction (TLC analysis), the solvent was carefully removed under reduced pressure. The residue was directly subjected to column chromatography to afford the desired product **3**, using CH_2Cl_2/acetone (from 150:1 to 100:1) as eluent.

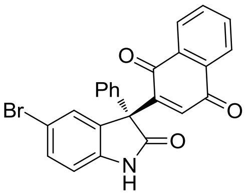

3a

Product **3a** was obtained in 70% yield as yellow solid; mp 192-194 °C; HPLC analysis (Chiralcel OD-H, iPrOH/hexane 20/80, 1.0 mL/min, 230 nm; t_r (minor) = 8.76 min, t_r (major) = 25.17 min) gave the product: 79% ee; $[\alpha]^{25}_D$ = +198.1 (c = 0.96, acetone); ^{1}H NMR (400 MHz, $CDCl_3$): δ 8.07-8.05 (m, 1H), 8.00-7.98 (m, 1H), 7.82 (s, 1H), 7.77-7.71 (m, 2H), 7.45-7.43 (m, 3H), 7.39-7.37 (m, 3H), 7.28 (d, J = 1.6 Hz, 1H), 6.90 (d, J = 8.4 Hz, 1H), 6.65 (s, 1H); ^{13}C NMR (100 MHz, $CDCl_3$): δ 184.66, 183.48, 178.45, 151.95, 140.55, 138.99, 135.01, 134.18, 134.09, 132.53, 132.09, 131.74, 128.96, 128.72, 128.61, 128.46, 127.18, 126.27, 114.63, 112.20, 59.77; IR (ATR): 3165, 1708, 1664, 1617, 1473, 1297, 1252, 1001, 779, 705; MS (EI): 445 [M(^{81}Br)$^+$, 76), 443 [M(^{79}Br)$^+$, 77], 444 [(M + H)$^+$, 26], 398, 400 (88, 100), 364 (9), 319 (20), 278 (31), 203 (26), 105 (26), 76 (51); HRMS (EI): Exact mass calcd for $C_{24}H_{14}NO_3{}^{79}Br$ $[M]^+$: 443.0157, found: 443.0155.

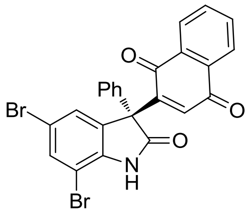

3b

Product **3b** was obtained in 97% yield as yellow solid; mp 283-285 °C; HPLC analysis (Chiralcel OD-H, iPrOH/hexane 20/80, 1.0 mL/min, 230 nm; t_r (minor) = 7.42 min, t_r (major) = 17.95 min) gave the product: 80% ee; $[\alpha]^{25}_{D}$ = +148.0 (*c* = 0.80, acetone); ^{1}H NMR (400 MHz, $CDCl_3$): δ 8.07-8.05 (m, 1H), 8.01-7.98 (m, 1H), 7.82 (s, 1H), 7.78-7.71 (m, 2H), 7.61 (d, *J* = 2.0 Hz, 1H), 7.42-7.39 (m, 5H), 7.22 (d, *J* = 1.6 Hz, 1H), 6.62 (s, 1H); ^{13}C NMR (100 MHz, $CDCl_3$): δ 184.59, 183.40, 176.84, 151.64, 140.19, 139.15, 134.35, 134.31, 134.25, 134.17, 133.02 131.79, 131.76, 129.14, 129.03, 128.58, 127.39, 127.25, 126.39, 114.80, 104.13, 60.85; IR (ATR): 3670, 2988, 2902, 1715, 1665, 1611, 1462, 1257, 1067, 801, 749; MS (EI): 523 $[M(^{79}Br^{81}Br)^+$, 52], 521 $[M(^{79}Br_2)^+$, 29], 526 $[(M + H)^+$, 7], 478 (100), 462 (17), 367 (23), 277 (23), 202 (32), 104 (34), 76 (68); HRMS (EI): Exact mass calcd for $C_{24}H_{13}NO_3{}^{79}Br_2$ $[M]^+$: 520.9262, found: 520.9257.

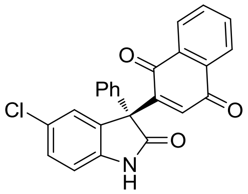

3c

Product **3c** was obtained in 74% yield as yellow solid; mp 175-177 °C; HPLC analysis (Chiralcel OD-H, iPrOH/hexane 20/80, 1.0 mL/min, 254 nm; t_r (minor) = 8.32 min, t_r (major) = 24.42 min) gave the product: 81% ee; $[\alpha]^{25}_{D}$ = +173.8 (*c* = 0.51, acetone); ^{1}H NMR (400 MHz, $CDCl_3$): δ 8.09-7.97 (m, 3H), 7.77-7.71 (m, 2H), 7.45-7.44 (m, 2H), 7.41-7.37 (m, 3H), 7.29 (dd, *J* = 8.4 Hz, *J* = 2.0 Hz, 1H), 7.15 (d, *J* = 2.0 Hz, 1H), 6.94 (dd, *J* = 8.4, *J* = 4.4, 1H), 6.66 (s, 1H); ^{13}C NMR (100 MHz, $CDCl_3$): δ 184.67, 183.46, 178.83, 151.88, 140.14, 138.94, 135.01, 134.15, 134.07, 132.09, 131.67, 129.15, 128.91, 128.67, 128.56, 127.34, 127.14, 126.23, 125.68, 111.82, 59.87; IR (ATR): 3012, 1717, 1665, 1616, 1478, 1301, 1255, 999, 817, 730; MS (EI): 401 $[M(^{37}Cl)^+$, 26], 399 $[M(^{35}Cl)^+$, 69], 400 $[(M + H)^+$, 20], 382 (22), 354, 356 (100, 44), 338 (15), 278 (15), 203 (13), 104 (11), 76 (25); HRMS (EI): Exact mass calcd for $C_{24}H_{14}NO_3{}^{35}Cl$ $[M]^+$: 399.0662, found: 399.0664.

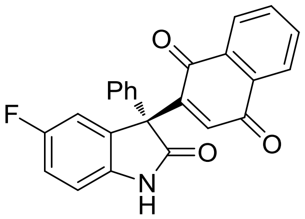

3d

Product **3d** was obtained in 71% yield as yellow solid; mp 245-247 °C; HPLC analysis (Chiralcel OD-H, ^{i}PrOH/hexane = 20/80, 1.0 mL/min, 230 nm; t_r (minor) = 8.79 min, t_r (major) = 25.82 min) gave the product: 79% ee; $[\alpha]^{25}_{D}$ = +191.9 (c = 0.61, acetone); ^{1}H NMR (400 MHz, $CDCl_3$): δ 8.07-8.04 (m, 1H), 8.00-7.97 (m, 1H), 7.76-7.70 (m, 3H), 7.46-7.45 (m, 2H), 7.39-7.36 (m, 3H), 7.03 (td, *J* = 8.8 Hz, *J* = 2.4 Hz, 1H), 6.96-6.93 (m, 2H), 6.67 (s, 1H); ^{13}C NMR (100 MHz, $CDCl_3$): δ 184.70, 183.46, 178.99, 159.71, 157.31, 151.87, 138.87, 135.24, 134.13, 134.07, 131.88, 131.68, 128.89, 128.60, 128.50, 127.13, 126.22, 115.70, 115.47, 113.60, 113.35, 111.44, 111.36, 60.20; ^{19}F NMR (376 MHz, $CDCl_3$): δ -120.01 (s, 1F); IR (ATR) : 3375, 3289, 3006, 1740, 1658, 1613, 1483, 1252, 999, 699; GC-MS: 383 (M^+, 46), 354 (13), 338 (100), 322 (23), 309 (19), 296(10), 105 (13), 76 (19); HRMS (EI): Exact mass calcd for $C_{24}H_{14}NO_3F$ $[M]^+$: 383.0958, found: 383.0955.

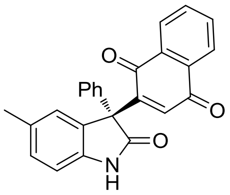

3e

Product **3e** was obtained in 46% yield as yellow solid; mp 173-175 °C; HPLC analysis (Chiralcel OD-H, iPrOH/hexane 20/80, 1.0 mL/min, 230 nm; t_r (minor) = 8.92 min, t_r (major) = 20.84 min) gave the product: 69% ee; $[\alpha]^{25}_{D}$ = +161.8 (*c* = 0.91, acetone); ^{1}H NMR (400 MHz, $CDCl_3$): δ 8.06-8.04 (m, 1H), 8.00-7.98 (m, 1H), 7.75-7.69 (m, 2H), 7.52-7.46 (m, 3H), 7.37-7.35 (m, 3H), 7.12 (d, *J* = 8.0 Hz, 1H), 6.97 (s, 1H), 6.90 (d, *J* = 8.0 Hz, 1H), 6.66 (s, 1H), 2.32 (s, 3H); ^{13}C NMR (100 MHz, $CDCl_3$): δ 184.95, 183.46, 178.96, 152.63, 138.95, 138.52, 135.97, 133.94, 133.93, 132.02, 131.71, 131.56, 130.40, 129.54, 128.74, 128.71, 128.35, 127.18, 126.19, 126.09, 110.47, 59.86, 21.14; IR (ATR): 3649, 2996, 2957, 1703, 1665, 1623, 1490, 1255, 894, 703; GC-MS: 379 (M^+, 68), 362 (30), 334 (100), 318 (21), 246 (30), 189 (14), 105 (10), 76 (12); HRMS (EI): Exact mass calcd for $C_{25}H_{17}NO_3$ $[M]^+$: 379.1208, found: 379.1211.

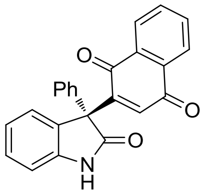

3f

Product **3f** was obtained in 51% yield as yellow solid; mp 286-288 °C; HPLC analysis (Chiralcel OD-H, iPrOH/hexane 20/80, 1.0 mL/min, 230 nm; t_r (minor) = 9.83 min, t_r (major) = 29.17 min) gave the product: 70% ee; $[\alpha]^{25}_{D}$ = +193.1 (*c* = 0.52, acetone); ^{1}H NMR (400 MHz, $CDCl_3$): δ 8.05-8.04 (m, 1H), 7.99-7.97 (m, 1H), 7.75-7.68 (m, 2H), 7.53 (s, 1H), 7.47-7.46 (m, 2H), 7.36-7.31 (m, 4H), 7.18 (d, *J* = 7.6 Hz, 1H), 7.07 (t, *J* = 7.6 Hz, 1H), 7.01 (d, *J* = 7.6 Hz, 1H), 6.67 (s, 1H); ^{13}C NMR (100 MHz, $CDCl_3$): δ 184.89, 183.50, 178.54, 152.58, 141.27, 138.65, 135.72, 134.01, 132.00, 131.74, 130.42, 129.23, 128.81, 128.78, 128.50, 127.15, 126.16, 125.61, 122.17, 110.58, 59.64; IR (ATR): 3664, 2989, 2959, 1712, 1664, 1615, 1472, 1254, 896, 755; GC-MS: 365 (M^+, 50), 320 (100), 291 (19), 246 (18), 207 (24), 105 (12), 76 (18), 44 (49); HRMS (EI): Exact mass calcd for $C_{24}H_{15}NO_3$ $[M]^+$: 365.1052, found: 365.1051.

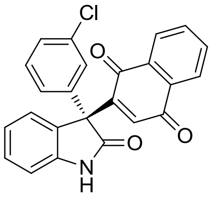

3g

Product **3g** was obtained in 76% yield as yellow solid; mp 164-166 °C; HPLC analysis (Chiralcel OD-H, iPrOH/hexane 20/80, 1.0 mL/min, 254 nm; t_r (minor) = 9.55 min, t_r (major) = 20.01 min) gave the product: 81% ee; $[\alpha]^{25}_{D}$ = +232.7 (*c* = 0.50, acetone); ^{1}H NMR (400 MHz, $CDCl_3$): δ 8.08-8.04 (m, 2H), 7.98-7.96 (m, 1H), 7.75-7.69 (m, 2H), 7.42 (s, 2H), 7.35-7.29 (m, 3H), 7.18 (d, *J* = 7.2 Hz, 1H), 7.08 (t, *J* = 7.2 Hz, 1H), 7.02 (d, *J* = 7.6 Hz, 1H), 6.67 (s, 1H); ^{13}C NMR (100 MHz, $CDCl_3$): δ 184.69, 183.30, 178.04, 151.94, 141.26, 138.49, 137.97, 134.77, 134.14, 134.10, 131.91, 131.69, 130.00, 129.65, 129.56, 128.78, 127.17, 127.08, 126.23, 125.64, 122.47, 110.83, 59.37; IR (ATR): 3700, 2918, 1711, 1664, 1616, 1473, 1257, 1099, 912, 783; GC-MS: 399 (M^+, 71), 355 (87), 319 (52), 290 (43), 207 (38), 105 (54), 76 (100), 44 (81); HRMS (EI): Exact mass calcd for $C_{24}H_{14}NO_3{}^{35}Cl$ $[M]^+$: 399.0662, found: 399.0645.

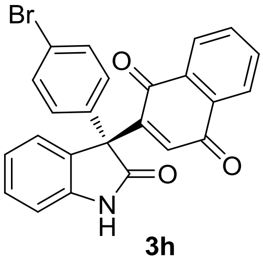

3h

Product **3h** was obtained in 45% yield as yellow solid; mp 180-182 °C; HPLC analysis (Chiralcel OD-H, iPrOH/hexane 20/80, 1.0 mL/min, 230 nm; t_r (minor) = 10.58 min, t_r (major) = 25.61 min) gave the product: 54% ee; $[\alpha]^{25}_{D}$ = +140.6 (c = 0.67, acetone); ^{1}H NMR (400 MHz, $CDCl_3$): δ 8.61 (s, 1H), 8.05-8.03 (m, 1H), 7.98-7.94 (m, 1H), 7.74-7.68 (m, 2H), 7.50-7.46 (m, 2H), 7.34-7.28 (m, 3H), 7.16 (d, J = 7.6 Hz, 1H), 7.06 (t, J = 7.6 Hz, 1H), 7.02-6.99 (m, 1H), 6.67 (s, 1H); ^{13}C NMR (100 MHz, $CDCl_3$): δ 184.67, 183.31, 178.46, 151.90, 141.38, 138.43, 135.03, 134.10, 134.07, 131.90, 131.64, 130.40, 129.80, 129.45, 127.15, 126.19, 125.52, 122.33, 110.92, 59.35; IR (ATR): 3664, 2987, 2901, 1710, 1660, 1615, 1394, 1254, 1066, 895, 754; MS (EI): 445 [M(^{81}Br)$^+$, 94], 443 [M(^{79}Br)$^+$, 91], 444 [(M + H)$^+$, 27], 400 (68), 364 (100), 319 (41), 290 (27), 278 (41), 203 (31), 105 (32), 76 (66); HRMS (EI): Exact mass calcd for $C_{24}H_{14}NO_3{}^{79}Br$ [M]$^+$: 443.0157, found: 443.0153.

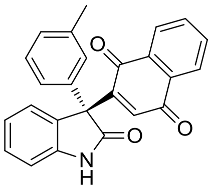

3i

Product **3i** was obtained in 55% yield as yellow solid; mp 270-272 °C; HPLC analysis (Chiralcel OD-H, iPrOH/hexane 20/80, 1.0 mL/min, 230 nm; t_r (minor) = 7.79 min, t_r (major) = 16.93 min) gave the product: 83% ee; $[\alpha]^{25}_{D}$ = +199.5 (c = 0.42, acetone); ^{1}H NMR (400 MHz, $CDCl_3$): δ 8.06-8.03 (m, 1H), 7.99-7.96 (m, 1H), 7.80 (s, 1H), 7.73-7.69 (m, 2H), 7.33-7.29 (m, 2H), 7.23-7.22 (m, 2H), 7.18-7.14 (m, 2H), 7.06 (td, J = 7.6 Hz, J = 1.2 Hz, 1H), 7.00 (d, J = 8.0 Hz, 1H), 6.69 (s, 1H), 2.32 (s, 3H); ^{13}C NMR (100 MHz, $CDCl_3$): δ 184.98, 183.51, 178.68, 152.65, 141.27, 138.70, 138.52, 135.54, 133.99, 132.01, 131.75, 130.56, 129.29, 129.14, 128.61, 127.14, 126.14, 125.80, 125.60, 122.11, 110.55, 59.59, 21.59; IR (ATR): 3240, 2927, 1717, 1663, 1595, 1472, 1256, 1000, 894, 748; GC-MS: 379 (M$^+$, 89), 361 (78), 281 (21), 254 (67), 226(100), 207 (51), 108 (39), 94 (45); HRMS (EI): Exact mass calcd for $C_{25}H_{17}NO_3$ [M]$^+$: 379.1208, found: 379.1204.

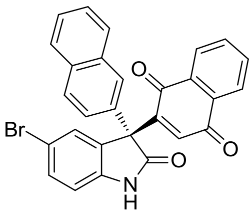

3j

Product **3j** was obtained in 68% yield as yellow solid; mp 262-264 °C; HPLC analysis (Chiralcel OD-H, iPrOH/hexane 20/80, 1.0 mL/min, 254 nm; t_r (minor) = 11.35 min, t_r (major) = 36.45 min) gave the product: 77% ee; $[\alpha]^{25}_{D} =$ +246.3 (*c* = 0.97, acetone); ^{1}H NMR (400 MHz, $CDCl_3$): δ 8.07-8.04 (m, 1H), 8.01-7.98 (m, 1H), 7.91-7.84 (m, 3H), 7.78-7.70 (m, 4H), 7.67 (s, 1H), 7.55-7.47 (m, 3H), 7.37 (d, *J* = 1.6 Hz, 1H), 6.93 (d, *J* = 8.0 Hz, 1H), 6.65 (s, 1H); ^{13}C NMR (100 MHz, $CDCl_3$): δ 184.58, 183.47, 178.06, 151.86, 140.46, 139.09, 134.23, 134.12, 133.05, 132.91, 132.47, 132.38, 132.24, 131.86, 131.75, 128.94, 128.55, 128.30, 127.91, 127.56, 127.20, 126.97, 126.54, 126.30, 125.96, 114.77, 112.17, 59.78; IR (ATR): 3662, 2990, 1713, 1666, 1594, 1478, 1257, 1066, 900, 746; MS (EI): 495 [M(^{81}Br)$^+$, 46], 493 [M(^{79}Br)$^+$, 42], 494 [(M + H)$^+$, 20], 448 (80), 450 (93), 328 (20), 258 (22), 207 (18), 105 (28), 76 (34), 44 (100); HRMS (EI): Exact mass calcd for $C_{28}H_{16}NO_3{}^{79}Br$ [M]$^+$: 493.0314, found: 493.0323.

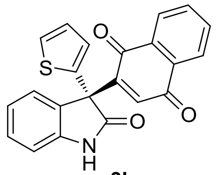

3k

Product **3k** was obtained in 53% yield as yellow solid; mp 231-233 °C; HPLC analysis (Chiralcel OD-H, iPrOH/hexane 20/80, 1.0 mL/min, 254 nm; t_r (minor) = 9.79 min, t_r (major) = 26.09 min) gave the product: 65% ee; $[\alpha]^{25}_{D} =$ +111.3 (*c* = 0.51, acetone); ^{1}H NMR (400 MHz, $CDCl_3$): δ 8.07-8.05 (m, 1H), 7.97-7.95 (m, 1H), 7.77-7.68 (m, 3H), 7.36-7.31 (m, 2H), 7.23 (d, *J* = 7.6 Hz, 1H), 7.19 (d, *J* = 2.8 Hz, 1H), 7.07-7.00 (m, 3H), 6.81 (s, 1H); ^{13}C NMR (100 MHz, $CDCl_3$): δ 184.85, 183.20, 177.99, 151.69, 141.16, 138.58, 138.32, 134.07, 134.03, 131.84, 131.79, 131.57, 129.50, 128.64, 127.24, 127.14, 127.09, 126.21, 124.16, 122.23, 110.79, 56.88; IR (ATR): 3391, 3339, 2923, 1728, 1659, 1590, 1471, 1299, 890, 764; GC-MS: 371 (M$^+$, 35), 338 (100), 327 (28), 281 (26), 253 (19), 207 (59), 76 (33), 44 (94); HRMS (EI): Exact mass calcd for $C_{22}H_{13}NO_3S$ [M]$^+$: 371.0616, found: 371.0612.

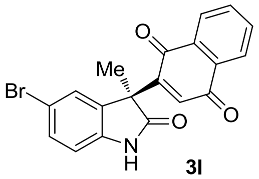

Product **3l** was obtained in 58% yield as yellow solid; mp 285-288 °C; HPLC analysis (Chiralcel OD-H, ^{i}PrOH/hexane = 20/80, 1.0 mL/min, 254 nm; t_r (minor) = 9.21 min, t_r (major) = 13.21 min) gave the product: 19% ee; $[\alpha]^{25}_{D}$ = -5.227 (c = 0.53, acetone); ^{1}H NMR (400 MHz, $CDCl_3$): δ 8.44 (s, 1H), 8.09 (d, J = 7.6 Hz, 1H), 7.92 (d, J = 7.6 Hz, 1H), 7.77-7.68 (m, 2H), 7.34 (dd, J = 8.0 Hz, J = 2.0 Hz, 1H), 7.21 (s, 1H), 7.05 (d, J = 1.2 Hz, 1H), 6.88 (d, J = 8.0 Hz, 1H), 1.66 (s, 3H); ^{13}C NMR (100 MHz, $CDCl_3$): δ 184.55, 183.35, 180.26, 149.28, 139.68, 136.77, 136.08, 134.18, 134.11, 131.79, 131.71, 131.40, 127.08, 126.29, 125.54, 114.92, 111.92, 50.50, 22.38; IR (ATR): 3387, 2999, 2951, 1735, 1662, 1586, 1475, 1290, 809, 748; GC-MS: 383 [M(^{81}Br)$^+$, 20], 381 [M(^{79}Br)$^+$, 21], 337 (100), 309 (57), 230 (58), 140 (20), 115 (57), 101 (76), 76 (71); HRMS (EI): Exact mass calcd for $C_{19}H_{12}NO_3{}^{79}Br$ $[M]^+$: 381.0001, found: 381.0003.

General procedure for the preparation of 13 from 3i.

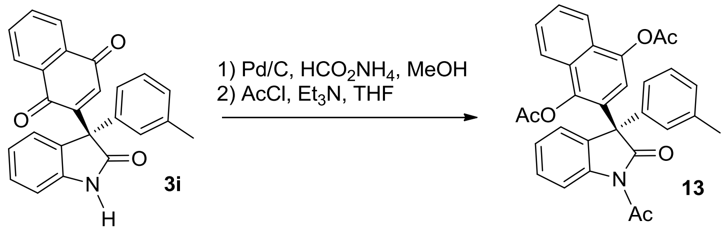

To a stirred solution of **3i** (27.5 mg, 0.072 mmol, 83% ee) and Pd/C (7.6 mg, 0.0072 mmol) in 1.0 mL MeOH was added HCO_2NH_4 (45.4 mg, 0.72 mmol) at room temperature. The resulting mixture was stirred until complete disappearance of **3i** as indicated by TLC analysis. The Pd/C was filtered and the filtrate was concentrated under reduced pressure. Anhydrous THF was added (2.0 mL) to the residue, followed by Et_3N (100 μL, 0.72 mmol) and AcCl (50 μL, 0.72 mmol). The reaction mixture was stirred at room temperature till full conversion. Then, 2 mL saturated $NaHCO_3$ (aq.) was added and extracted with ethyl acetate (3 × 10 mL). The organic phase was combined, dried and concentrated under reduced pressure. The residue was purified by silica gel column chromatography (petroleum ether/ethyl acetate 8/1) to afford the

desired product **13** (21.0 mg) in 57% yield for two steps as white soild. mp 144-146 °C; HPLC analysis (Chiralcel OZ-H, iPrOH/hexane 10/90, 1.0 mL/min, 230 nm; t_r (minor) = 14.13 min, t_r (major) = 28.49 min) gave the product: 84% ee; $[\alpha]^{25}_{D}$ = +130.9 (*c* = 1.00, acetone); ^{1}H NMR (400 MHz, $CDCl_3$): δ 8.35-8.33 (m, 1H), 7.85-7.83 (m, 1H), 7.54-7.38 (m, 5H), 7.25-7.19 (m, 4H), 7.09 (d, *J* = 7.6 Hz, 1H), 6.92 (s, 1H), 2.71 (s, 3H), 2.39 (s, 3H), 2.33 (s, 3H), 1.97 (s, 3H); ^{13}C NMR (100 MHz, $CDCl_3$): δ 177.61, 171.33, 168.91, 167.13, 144.24, 142.61, 139.19, 138.56, 136.93, 130.47, 129.74, 129.35, 129.01, 128.60, 127.75, 127.28, 127.19, 127.13, 126.65, 125.65, 122.22, 121.60, 119.43, 116.55, 60.04, 26.65, 21.57, 20.96, 19.75; IR (ATR): 3073, 1768, 1711, 1601, 1463, 1368, 1273, 1183, 1160, 908, 756; MS (EI): 507 (M^+, 4), 508 [$(M + H)^+$, 1], 465 (32), 423 (26), 381 (100), 336 (24), 319 (9), 202 (3), 105 (4), 43 (38); HRMS (EI): Exact mass calcd for $C_{31}H_{25}NO_6$ $[M]^+$: 507.1682, found: 507.1679.

One-pot procedure for the synthesis of 13 from 1i and 2.

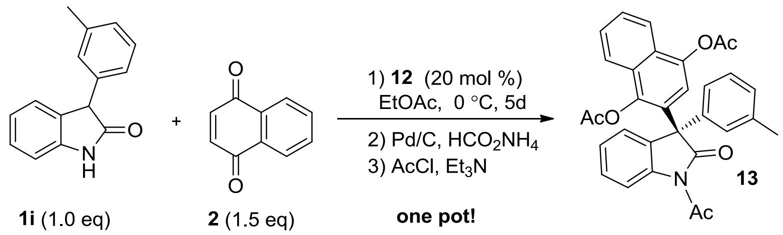

To a 25 mL Schlenk tube were added catalyst **12** (44.0 mg, 0.05 mmol) and 3-substituted oxindole **1i** (55.8 mg, 0.25 mmol), followed by 5 mL of anhydrous ethyl acetate under N_2 atmosphere. The resulting mixture was stirred at 0 °C for about 20 min before 1,4-naphthoquinone (**2**, 59.3 mg, 0.375 mmol) was added. After disappearance of oxindole **1i** (TLC analysis), Pd/C (26.5 mg, 0.025 mmol) and $HCOONH_4$ (157.5 mg, 2.5 mmol) were added and the reaction mixture was stirred at room temperature till full conversion. Then, Et_3N (0.35 mL, 2.5 mmol) and AcCl (179 μL, 2.5 mmol) were added at room temperature. After the reaction was finished, the mixture was treated with 5 mL sat. $NaHCO_3$. Then, the Pd/C was filtered and the filtrate was extracted with ethyl acetate (3 × 10 mL). The organic phase was combined,

dried and concentrated under reduced pressure and the residue was purified by silica gel column chromatography (petroleum ether/ethyl acetate 8/1) to afford product **13** (63.4 mg) in 50% yield for one pot as white solid. HPLC analysis (Chiralcel OZ-H, iPrOH/hexane 10/90, 1.0 mL/min, 230 nm; t_r (minor) = 14.22 min, t_r (major) = 28.53 min) gave the isomeric composition of the product: 75% ee.

Determination of the absolute configuration.

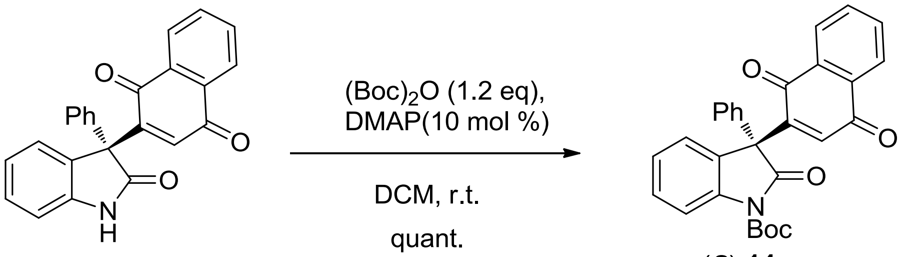

(*S*)-**3f** (*S*)-**14**

The absolute configuration of **3f** was assigned via transformation to the known compound **14**. To a 10 mL Schlenk tube were added **3f** (18.8 mg, 0.052 mmol, 70% ee) and DMAP (0.6 mg，0.0052 mmol), followed by anhydrous CH_2Cl_2 (1 mL) under N_2 atmosphere. The resulting mixture was stirred at room temperature and di-*tert*-butyl dicarbonate (13.6 mg, 0.06 mmol) was added [2]. After consumption of **3f (**TLC analysis) the organic phase was directly charged on silica gel and subjected to FC, quantitatively obtaining the compound **14** as a yellow solid: 23.9 mg; HPLC analysis (Chiralcel IC, iPrOH/hexane 20/80, 1.0 mL/min, 254 nm; t_r (minor) = 7.11 min, t_r (major) = 9.01 min) gave the product: 69% ee; ^{1}H NMR (400 MHz, $CDCl_3$): δ 8.05-7.95 (m, 3H), 7.75-7.67 (m, 2H), 7.44-7.34 (m, 6H), 7.21-7.12 (m, 2H), 6.65 (s, 1H), 1.65 (s, 9H); ^{13}C NMR (100 MHz, $CDCl_3$): δ 184.79, 183.14, 174.70, 152.98, 149.27, 140.23, 138.59, 135.27, 134.10, 134.05, 131.79, 131.71, 129.43, 129.13, 128.90, 128.72, 128.42, 127.30, 126.20, 124.95, 124.01, 116.06, 84.48, 59.69, 28.11. $[\alpha]^{25}_D$ = +75.0 (c = 1.15, CH_2Cl_2); reported rotation for the known compound (*R*)-**14** $[\alpha]^{25}_{D}$ = -113.7 (c = 2.0, CH_2Cl_2) [3].

References

1. Hamashima, Y.; Suzuki, T.; Takano, H.; Shimura, Y. ; Sodeoka, M. *J. Am. Chem. Soc.* **2005**, *127*, 10164.
2. Ding, M.; Zhou, F.; Qian, Z.-Q.; Zhou, J. *Org. Biomol. Chem.* **2010**, *8*, 2912.
3. Siau, W.-Y.; Li, W.; Xue, F.; Ren, Q.; Xu, M.; Sun, S.; Guo, H.; Jiang, X.; Wang, J. *Chem. Eur. J*. **2012**, DOI: 10.1002/chem.201200206.

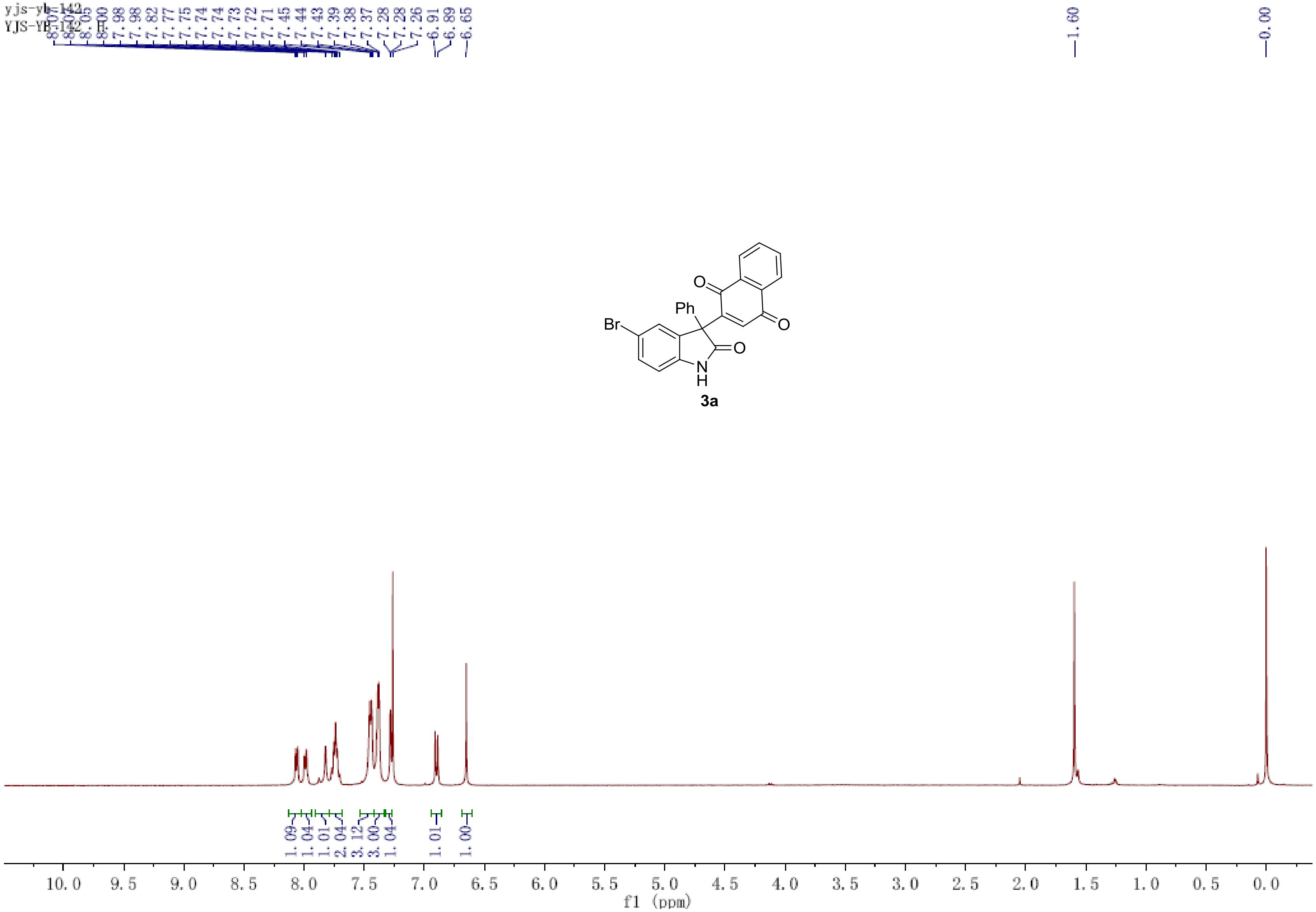

yjs-yb-142
YJS-YB-142_H
8.07
8.05
8.05
8.00
7.98
7.98
7.82
7.77
7.75
7.74
7.74
7.73
7.72
7.71
7.45
7.44
7.43
7.39
7.38
7.37
7.28
7.28
7.26
6.91
6.89
6.65
1.60
0.00
Br
Ph
O
O
O
N
H
3a
1.09
1.04
1.01
2.04
3.12
3.00
1.04
1.01
1.00
10.0
9.5
9.0
8.5
8.0
7.5
7.0
6.5
6.0
5.5
5.0
4.5
4.0
3.5
3.0
2.5
2.0
1.5
1.0
0.5
0.0
f1 (ppm)

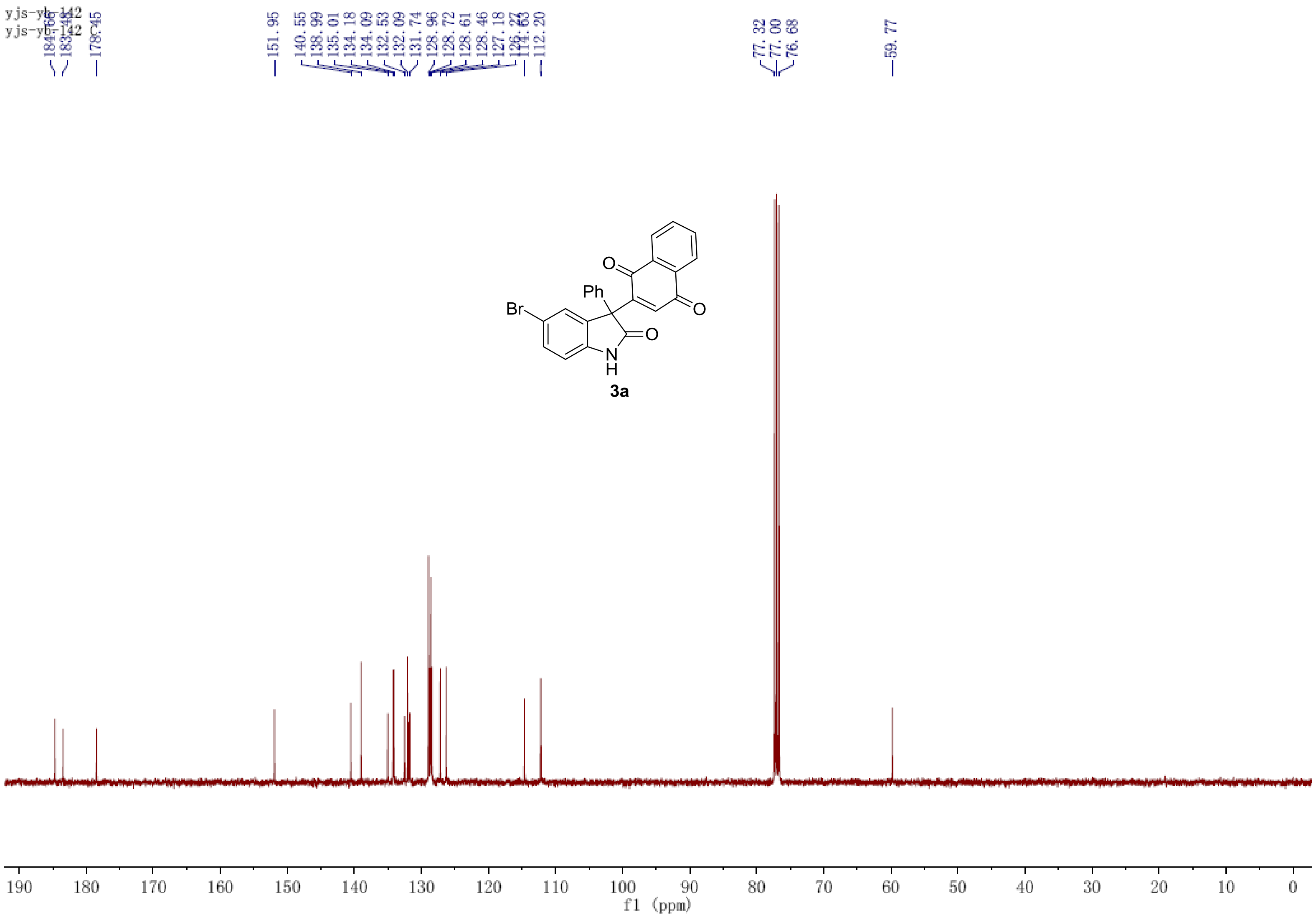
yjs-yb-142
yjs-yb-142 C
178.45
151.95
140.55
138.99
135.01
134.18
134.09
132.53
132.09
131.74
128.96
128.72
128.61
128.46
127.18
112.20
77.32
77.00
76.68
59.77
Br
Ph
O
O
O
N
H
3a
190
180
170
160
150
140
130
120
110
100
90
80
70
60
50
40
30
20
10
0
f1 (ppm)

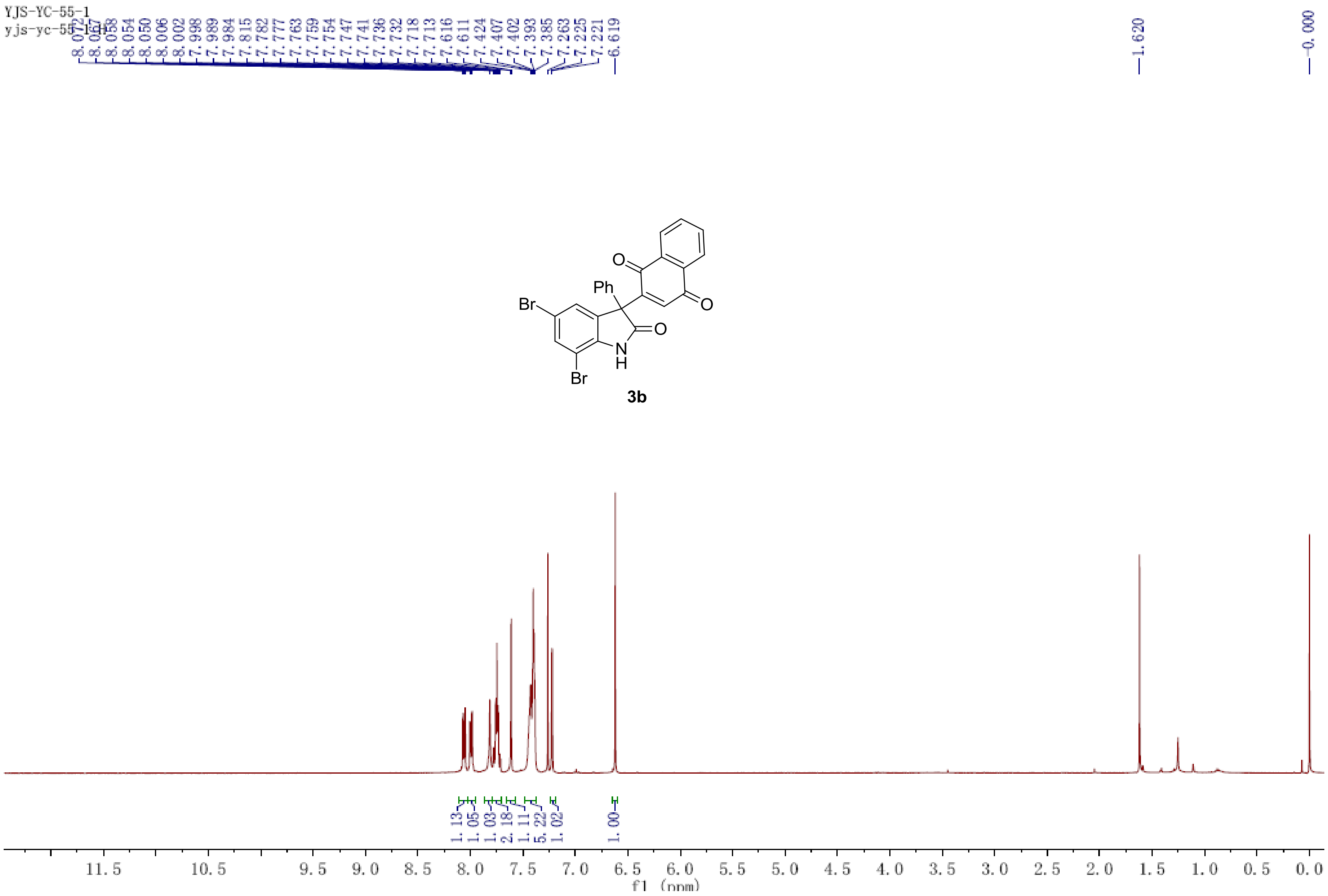
YJS-YC-55-1
8.072
8.067
8.058
8.054
8.050
8.006
8.002
7.998
7.989
7.984
7.815
7.782
7.777
7.763
7.759
7.754
7.747
7.741
7.736
7.732
7.718
7.713
7.616
7.611
7.424
7.407
7.402
7.393
7.385
7.263
7.225
7.221
6.619
1.620
0.000
1.13
1.05
1.03
2.18
1.11
5.22
1.02
1.00
11.5
10.5
9.5
9.0
8.5
8.0
7.5
7.0
6.5
6.0
5.5
5.0
4.5
4.0
3.5
3.0
2.5
2.0
1.5
1.0
0.5
0.0
f1 (ppm)
Ph
Br
O
O
O
N
H
Br

3b

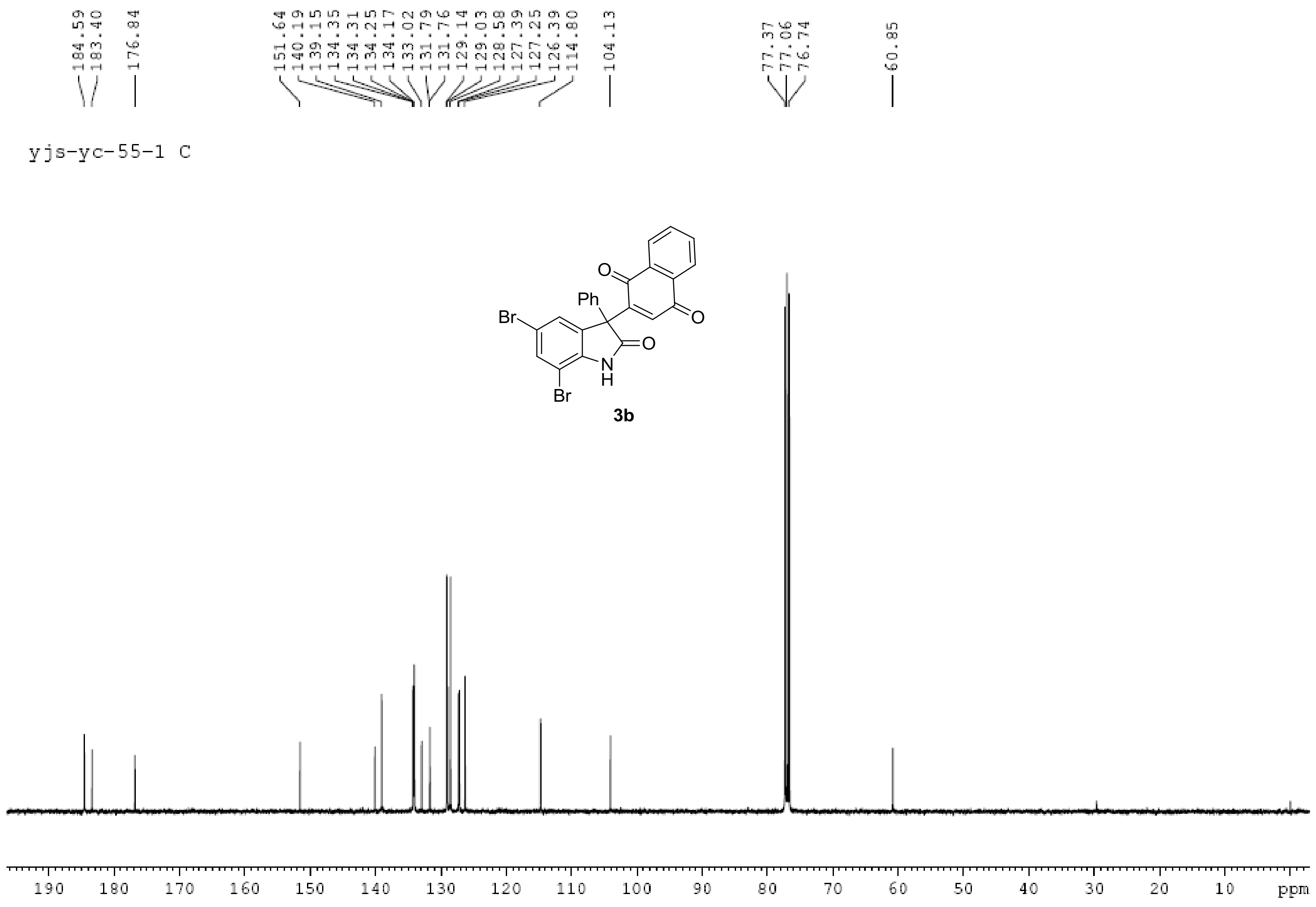
184.59
183.40
176.84
151.64
140.19
139.15
134.35
134.31
134.25
134.17
133.02
131.79
131.76
129.14
129.03
128.58
127.39
127.25
126.39
114.80
104.13
77.37
77.06
76.74
60.85
yjs-yc-55-1 C
Ph
Br
O
N
H
Br
3b
190 180 170 160 150 140 130 120 110 100 90 80 70 60 50 40 30 20 10 ppm

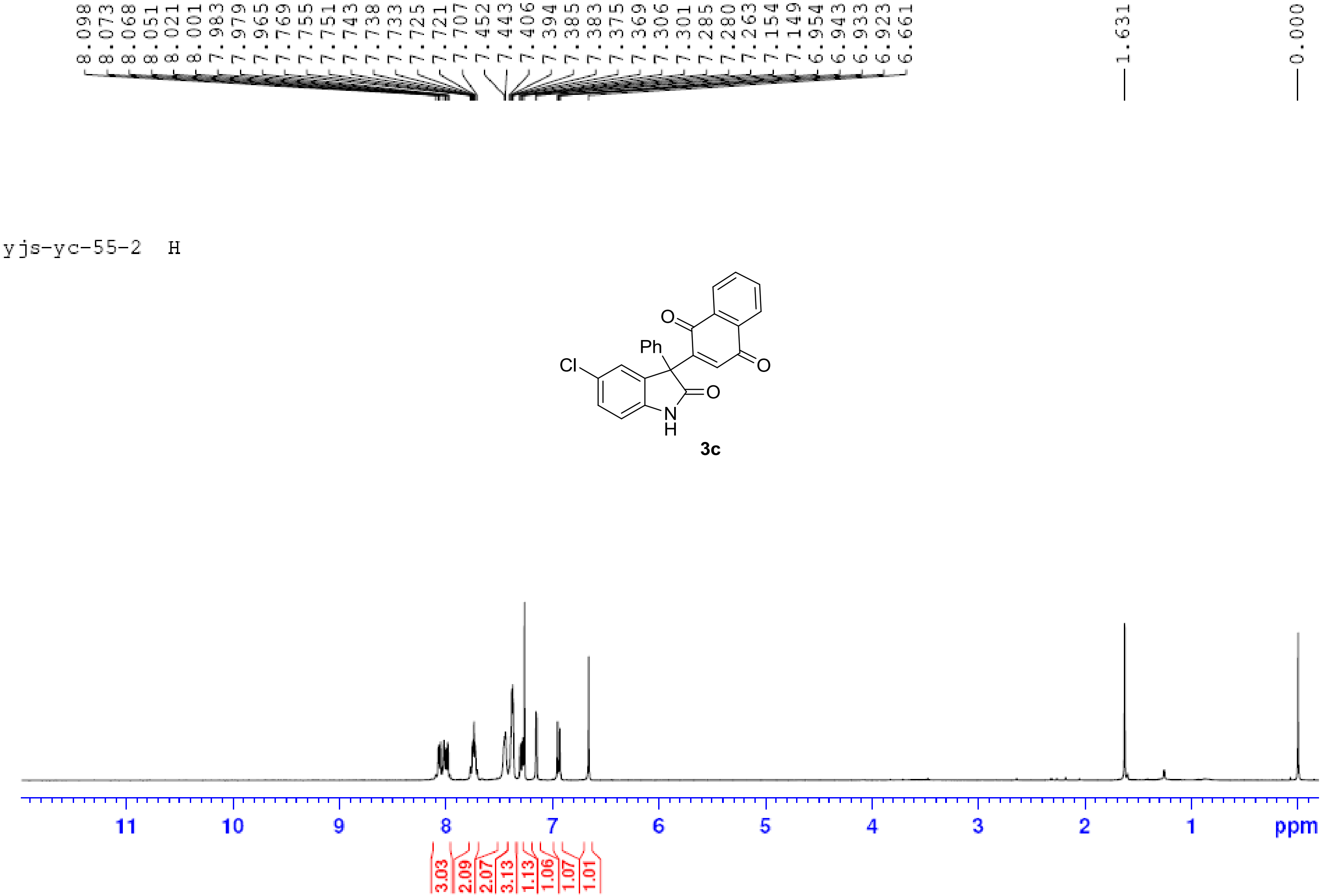
8.098
8.073
8.068
8.051
8.021
8.001
7.983
7.979
7.965
7.769
7.755
7.751
7.743
7.738
7.733
7.725
7.721
7.707
7.452
7.443
7.406
7.394
7.385
7.383
7.375
7.369
7.306
7.301
7.285
7.280
7.263
7.154
7.149
6.954
6.943
6.933
6.923
6.661
1.631
0.000
yjs-yc-55-2 H
Ph
Cl
O
O
O
N
H
11
10
9
8
7
6
5
4
3
2
1
ppm
3.03
2.09
2.07
3.13
1.13
1.06
1.07
1.01
3c

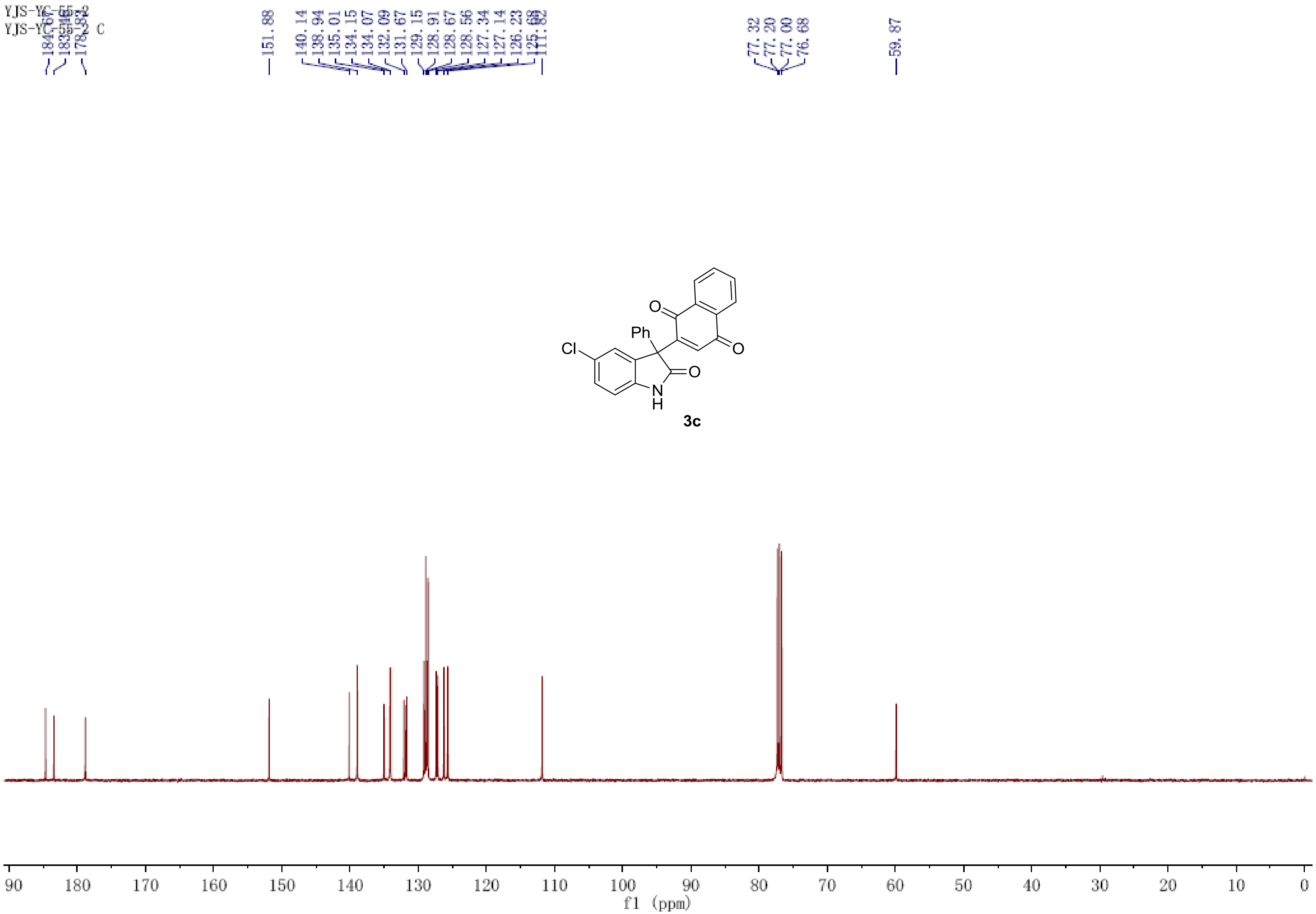
151.88
140.14
138.94
135.01
134.15
134.07
132.09
131.67
129.15
128.91
128.67
128.56
127.34
127.14
126.23
77.32
77.20
77.00
76.68
59.87
Ph
Cl
O
N
H
3c
f1 (ppm)

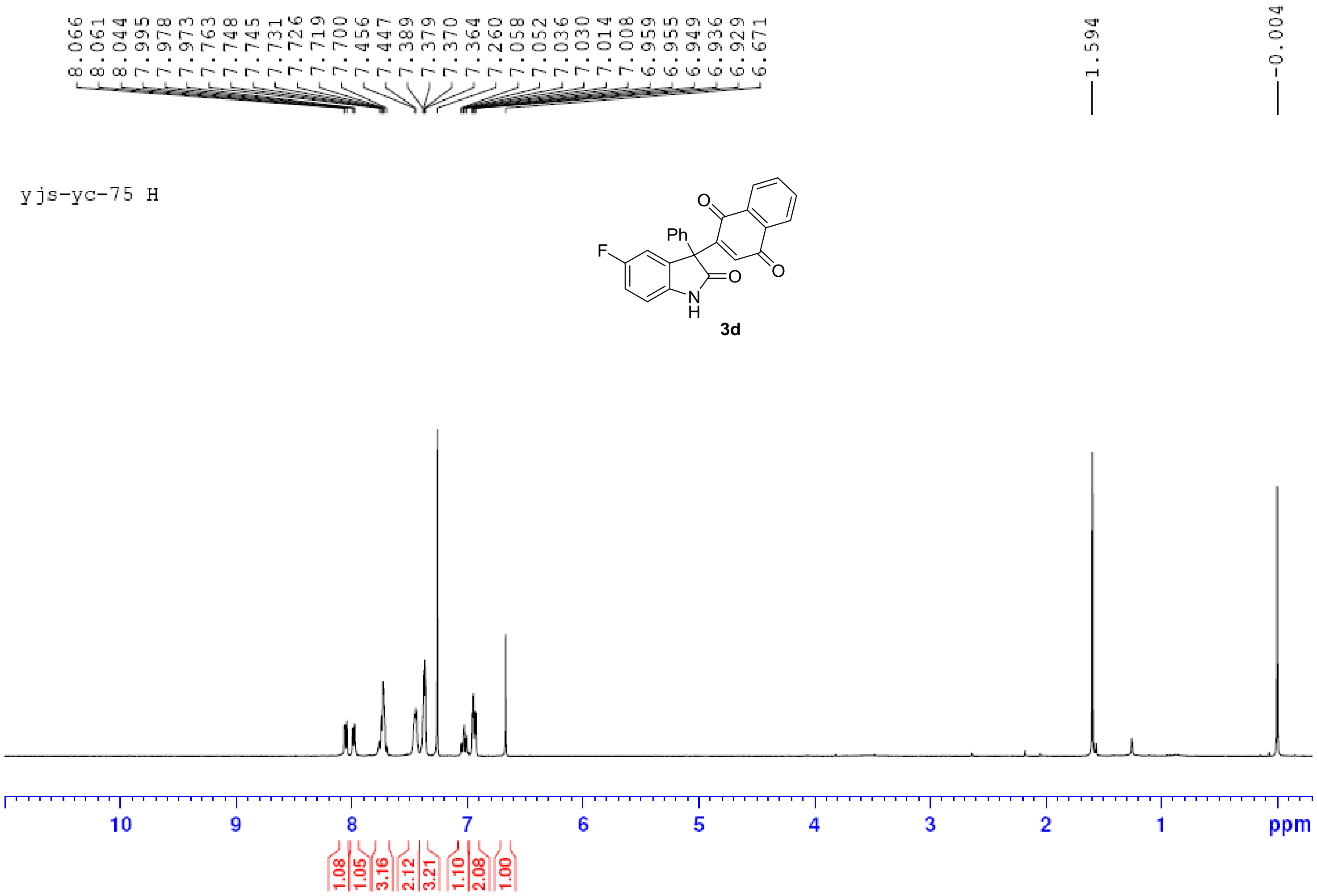

yjs-yc-75 H
8.066
8.061
8.044
7.995
7.978
7.973
7.763
7.748
7.745
7.731
7.726
7.719
7.700
7.456
7.447
7.389
7.379
7.370
7.364
7.260
7.058
7.052
7.036
7.030
7.014
7.008
6.959
6.955
6.949
6.936
6.929
6.671
1.594
-0.004
F
Ph
O
O
O
N
H
3d
1.08
1.05
3.16
2.12
3.21
1.10
2.08
1.00
10
9
8
7
6
5
4
3
2
1
ppm

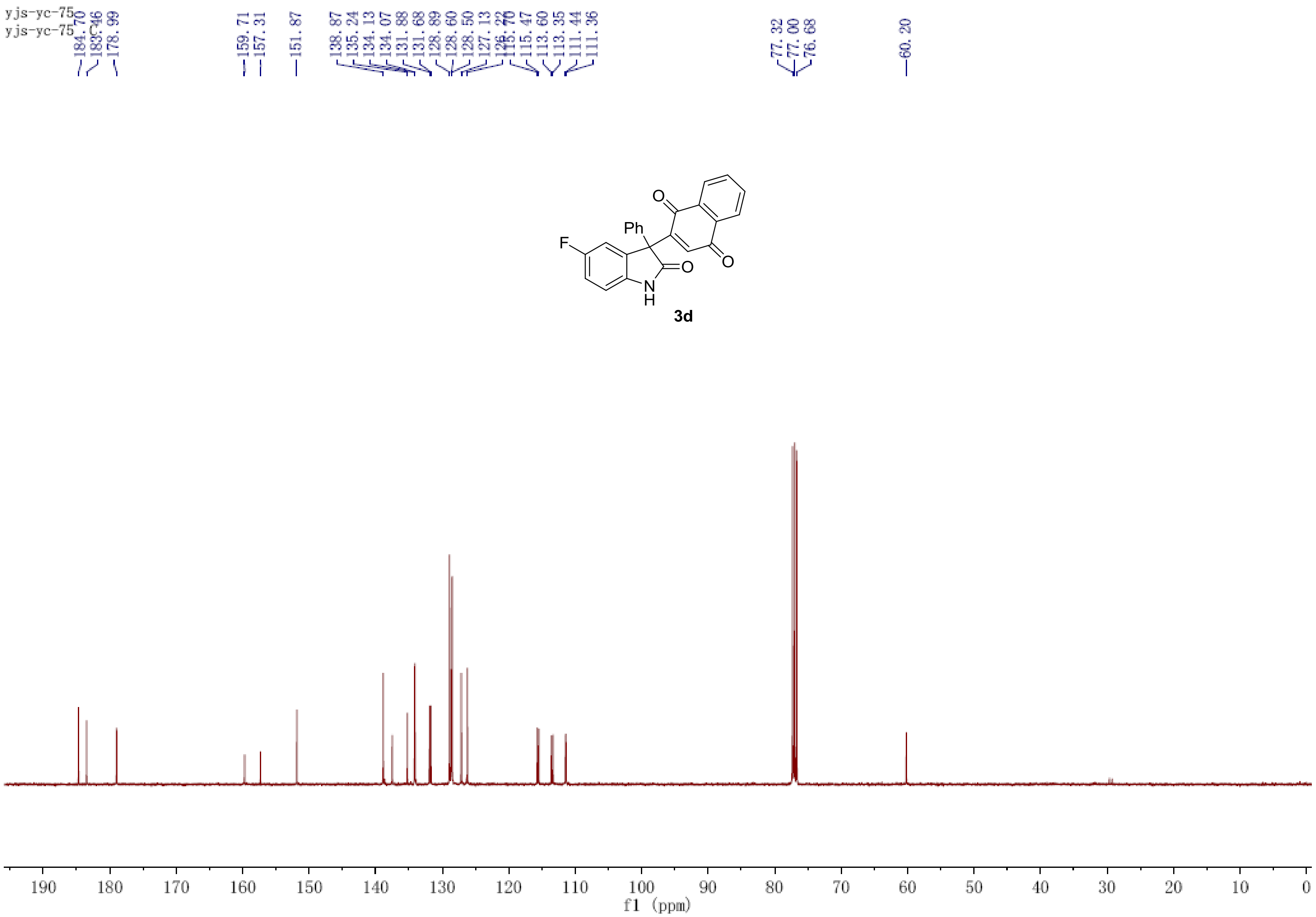

yjs-yc-75
yjs-yc-75_C
184.70
183.46
178.99
159.71
157.31
151.87
138.87
135.24
134.13
134.07
131.88
131.68
128.89
128.60
128.50
127.13
115.47
113.60
113.35
111.44
111.36
77.32
77.00
76.68
60.20
Ph
F
O
N
H
3d
f1 (ppm)

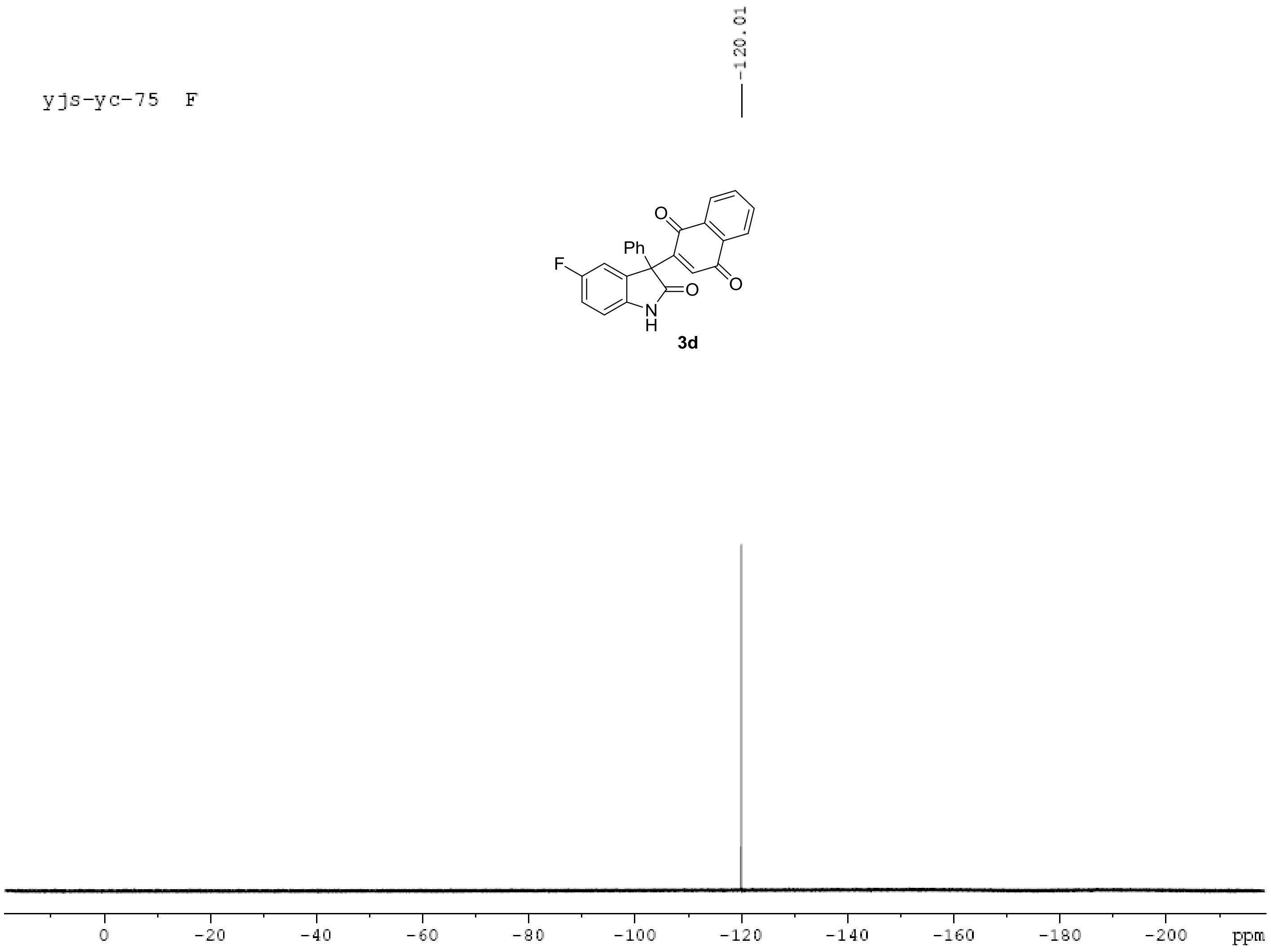
yjs-yc-75 F
-120.01
Ph
F
O
O
O
N
H
3d
0
-20
-40
-60
-80
-100
-120
-140
-160
-180
-200
ppm

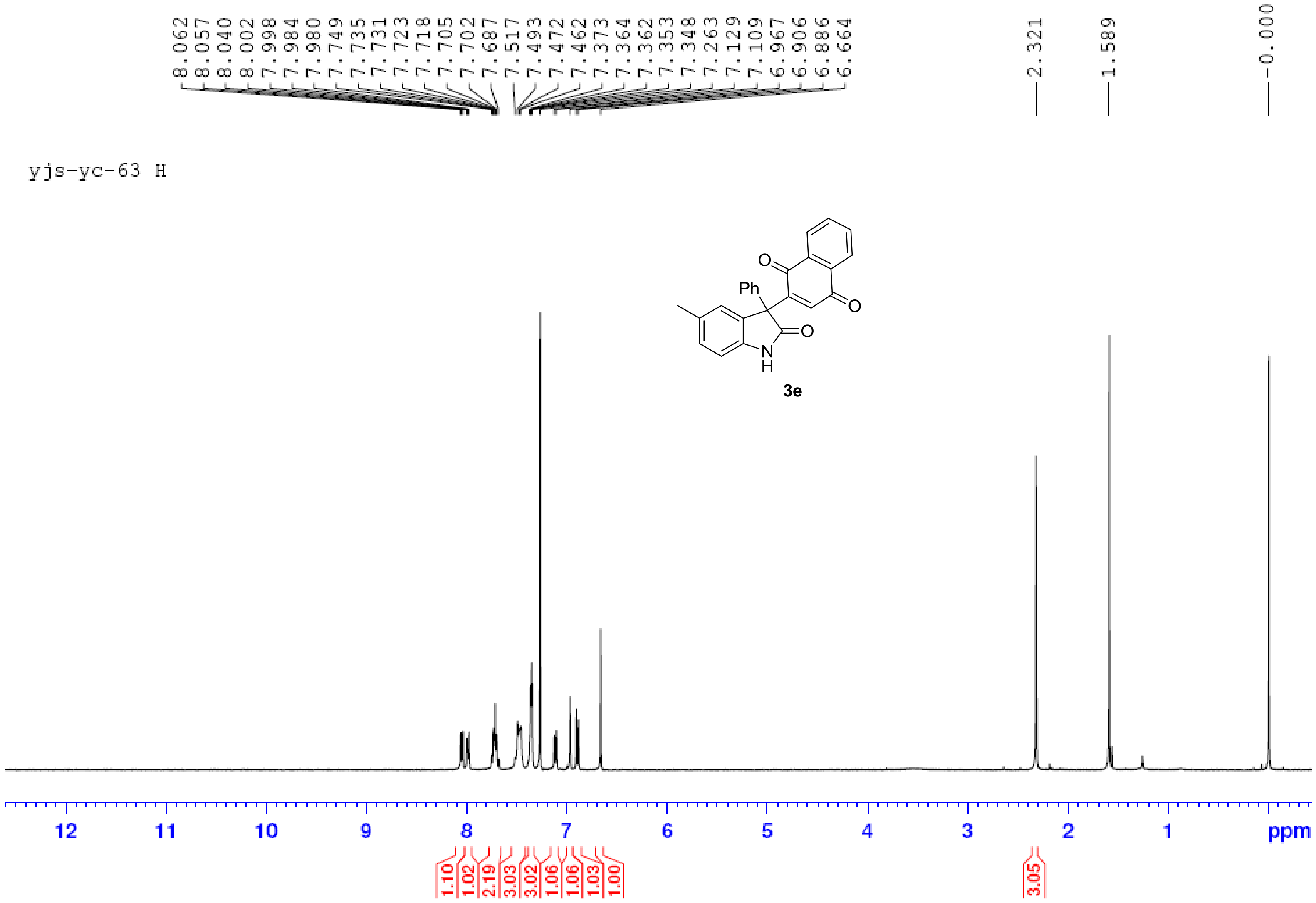
8.062 8.057 8.040 8.002 7.998 7.984 7.980 7.749 7.735 7.731 7.723 7.718 7.705 7.702 7.687 7.517 7.493 7.472 7.462 7.373 7.364 7.362 7.353 7.348 7.263 7.129 7.109 6.967 6.906 6.886 6.664
2.321
1.589
-0.000
yjs-yc-63 H
Ph
O
O
O
N
H
3e
12 11 10 9 8 7 6 5 4 3 2 1 ppm
1.10 1.02 2.19 3.03 3.02 1.06 1.06 1.03 1.00
3.05

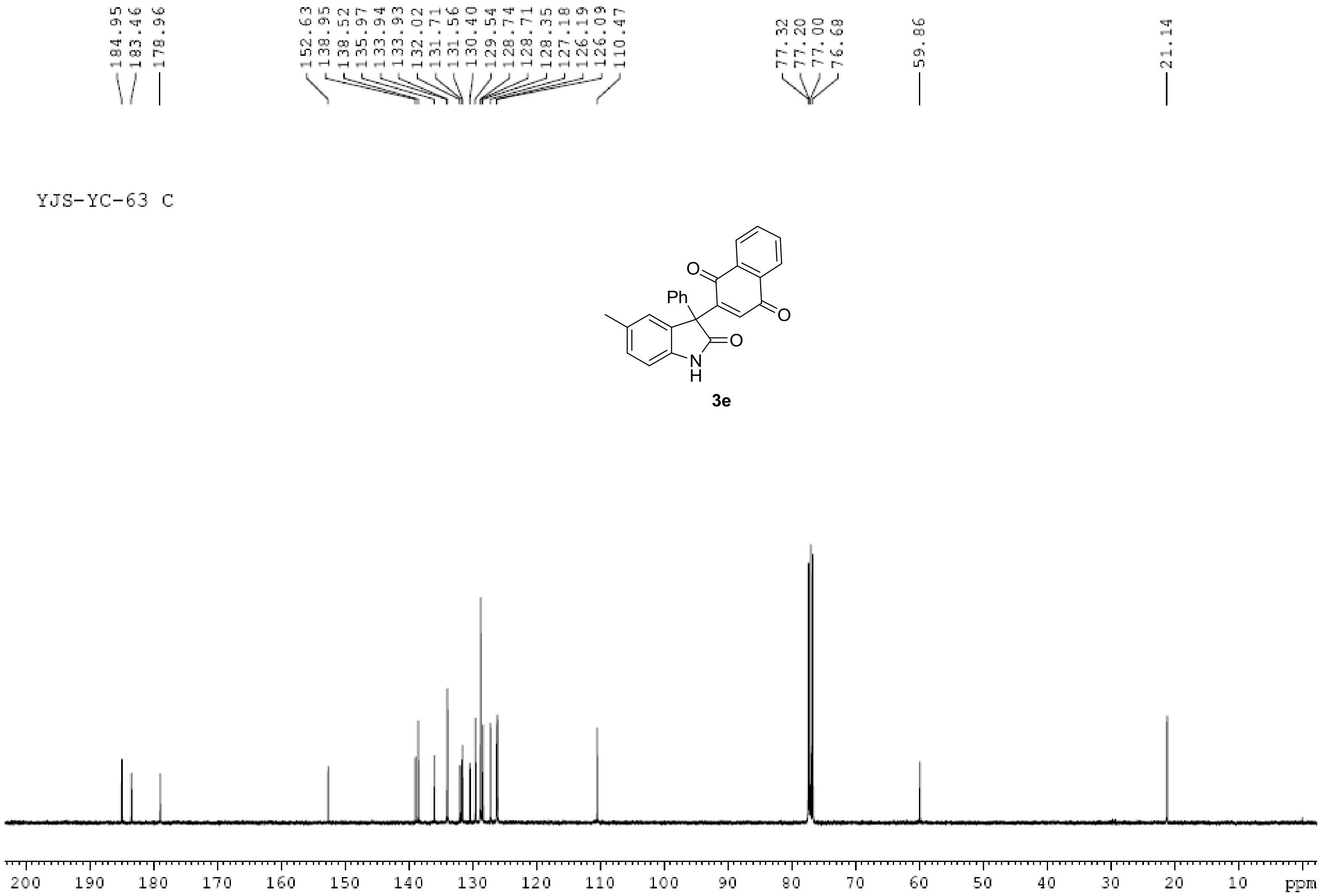
184.95
183.46
178.96
152.63
138.95
138.52
135.97
133.94
133.93
132.02
131.71
131.56
130.40
129.54
128.74
128.71
128.35
127.18
126.19
126.09
110.47
77.32
77.20
77.00
76.68
59.86
21.14
YJS-YC-63 C
Ph
O
N
H
3e
200 190 180 170 160 150 140 130 120 110 100 90 80 70 60 50 40 30 20 10 ppm

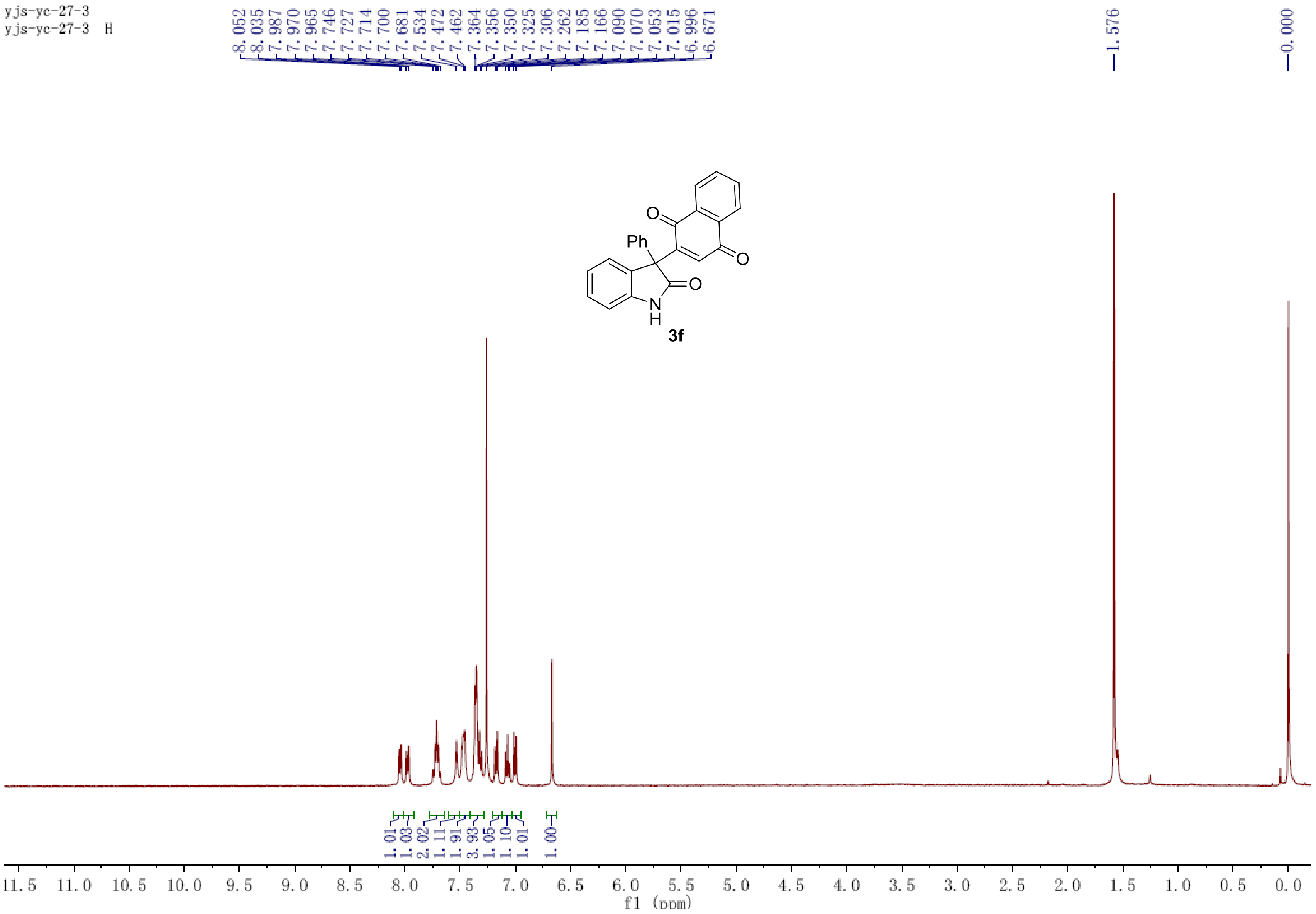
yjs-yc-27-3
yjs-yc-27-3 H
8.052 8.035 7.987 7.970 7.965 7.746 7.727 7.714 7.700 7.681 7.534 7.472 7.462 7.364 7.356 7.350 7.325 7.306 7.262 7.185 7.166 7.090 7.070 7.053 7.015 6.996 6.671
1.576
0.000
Ph
O
O
O
N
H
3f
1.01 1.03 2.02 1.11 1.91 3.93 1.05 1.10 1.01 1.00
11.5 11.0 10.5 10.0 9.5 9.0 8.5 8.0 7.5 7.0 6.5 6.0 5.5 5.0 4.5 4.0 3.5 3.0 2.5 2.0 1.5 1.0 0.5 0.0
f1 (ppm)

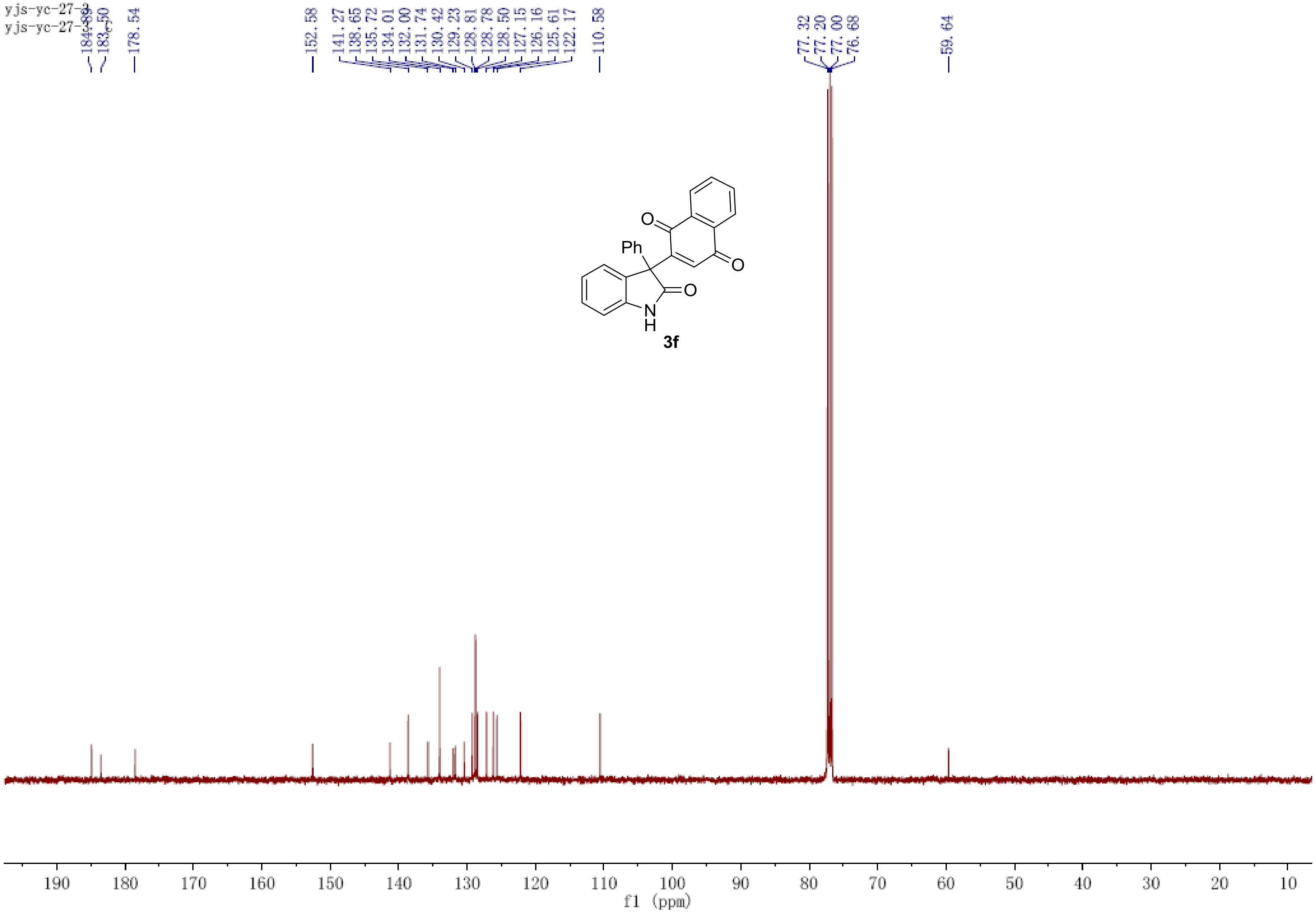
yjs-yc-27-3
yjs-yc-27-3-C
184.89
183.50
178.54
152.58
141.27
138.65
135.72
134.01
132.00
131.74
130.42
129.23
128.81
128.78
128.50
127.15
126.16
125.61
122.17
110.58
77.32
77.20
77.00
76.68
59.64
Ph
O
O
O
N
H
3f
190
180
170
160
150
140
130
120
110
100
90
80
70
60
50
40
30
20
10
f1 (ppm)

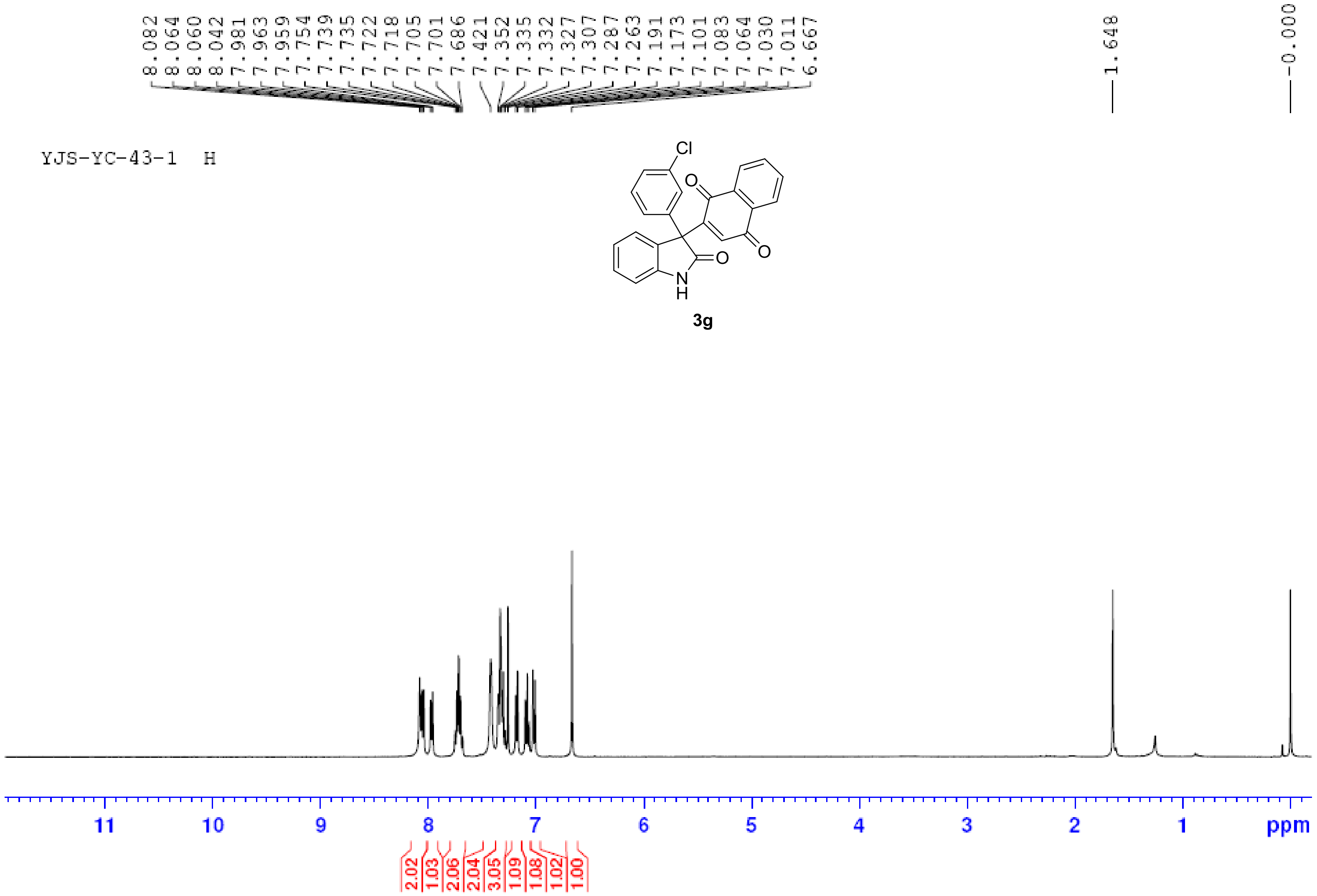
8.082
8.064
8.060
8.042
7.981
7.963
7.959
7.754
7.739
7.735
7.722
7.718
7.705
7.701
7.686
7.421
7.352
7.335
7.332
7.327
7.307
7.287
7.263
7.191
7.173
7.101
7.083
7.064
7.030
7.011
6.667
1.648
-0.000
YJS-YC-43-1 H
Cl
O
O
O
N
H
3g
11
10
9
8
7
6
5
4
3
2
1
ppm
2.02
1.03
2.06
2.04
3.05
1.09
1.08
1.02
1.00

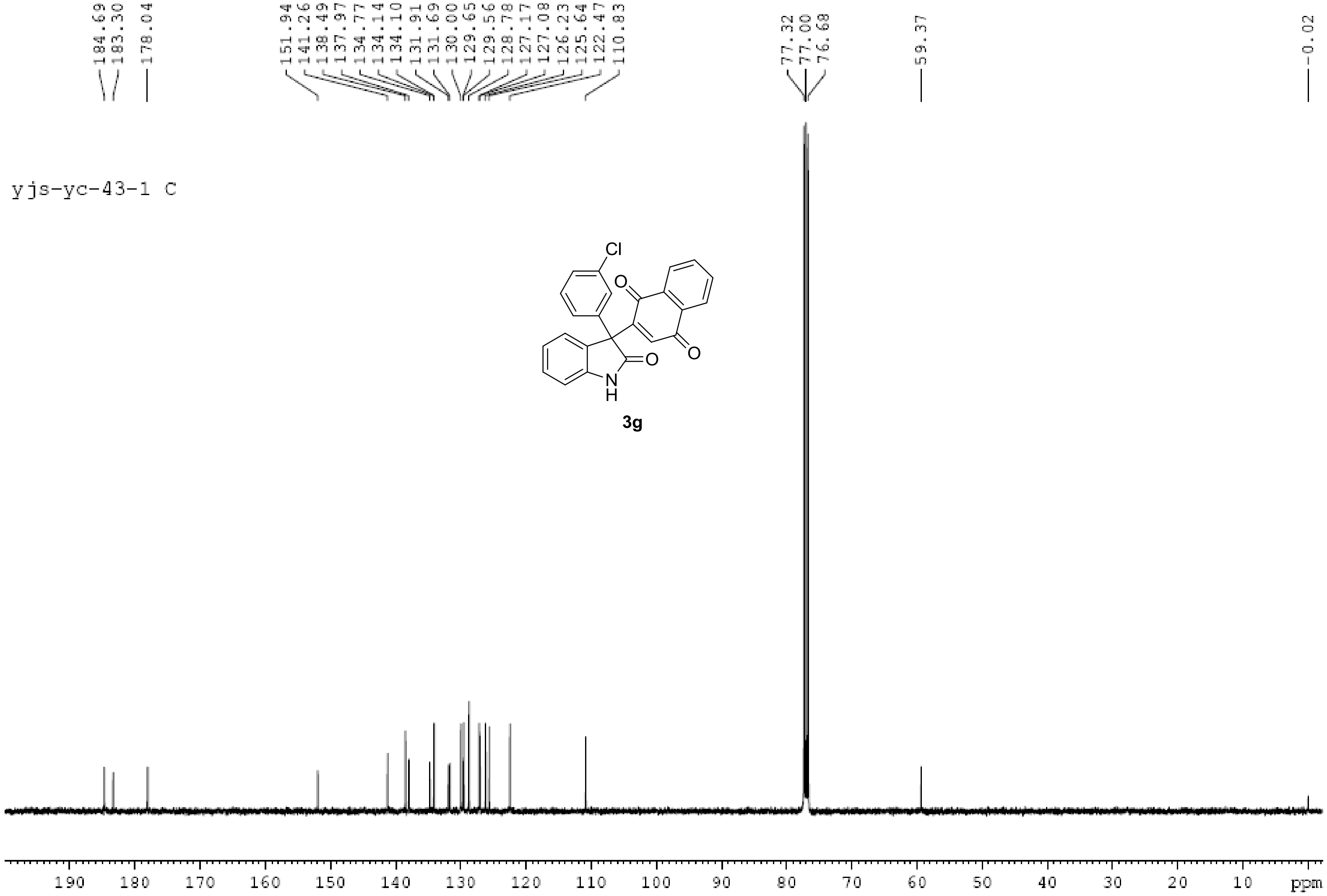
184.69
183.30
178.04
151.94
141.26
138.49
137.97
134.77
134.14
134.10
131.91
131.69
130.00
129.65
129.56
128.78
127.17
127.08
126.23
125.64
122.47
110.83
77.32
77.00
76.68
59.37
-0.02
yjs-yc-43-1 C
Cl
O
O
O
N
H
3g
190 180 170 160 150 140 130 120 110 100 90 80 70 60 50 40 30 20 10 ppm

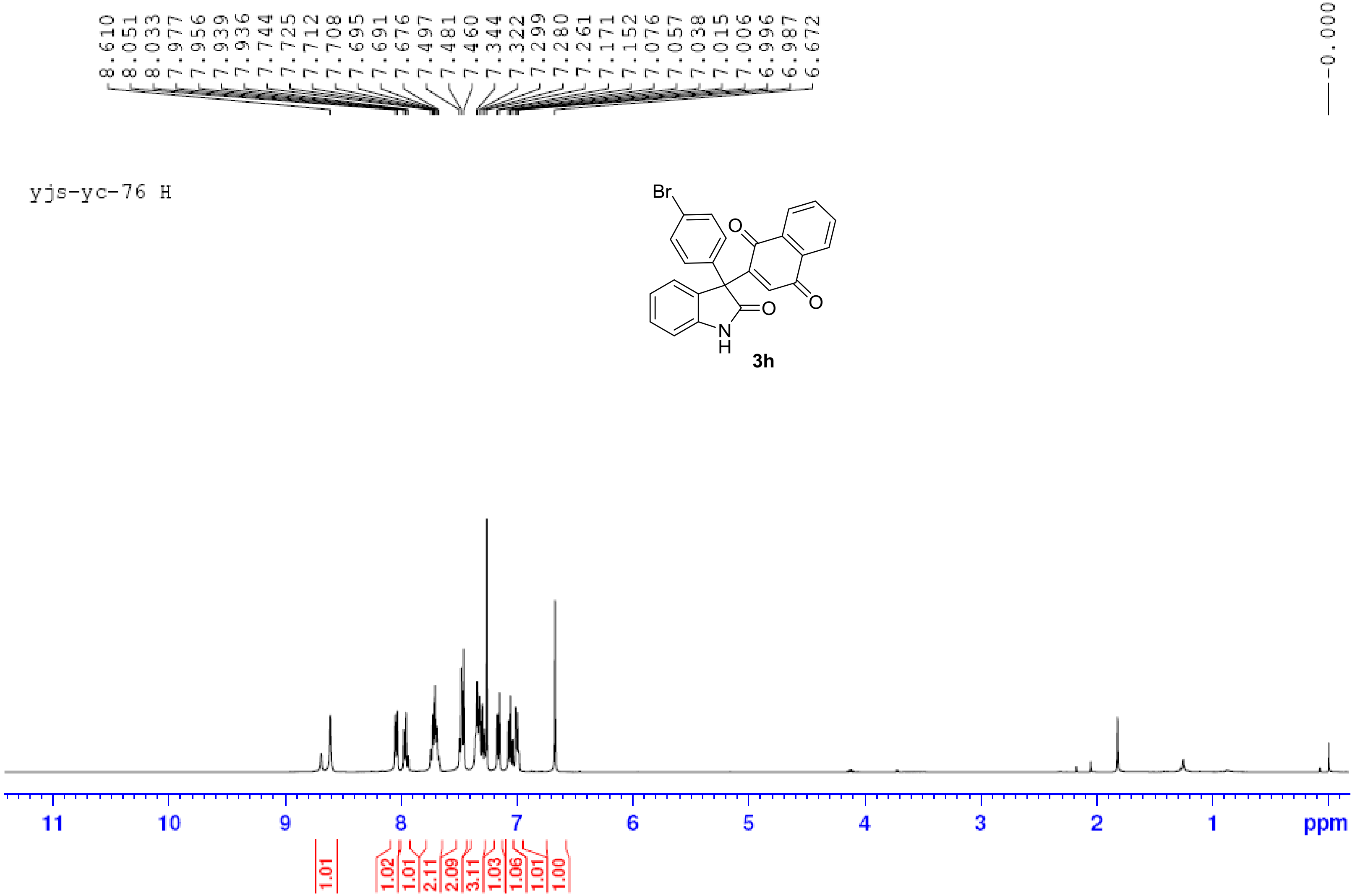
8.610
8.051
8.033
7.977
7.956
7.939
7.936
7.744
7.725
7.712
7.708
7.695
7.691
7.676
7.497
7.481
7.460
7.344
7.322
7.299
7.280
7.261
7.171
7.152
7.076
7.057
7.038
7.015
7.006
6.996
6.987
6.672
-0.000
yjs-yc-76 H
Br
O
O
O
N
H
3h
11 10 9 8 7 6 5 4 3 2 1 ppm
1.01
1.02
1.01
2.11
2.09
3.11
1.03
1.06
1.01
1.00

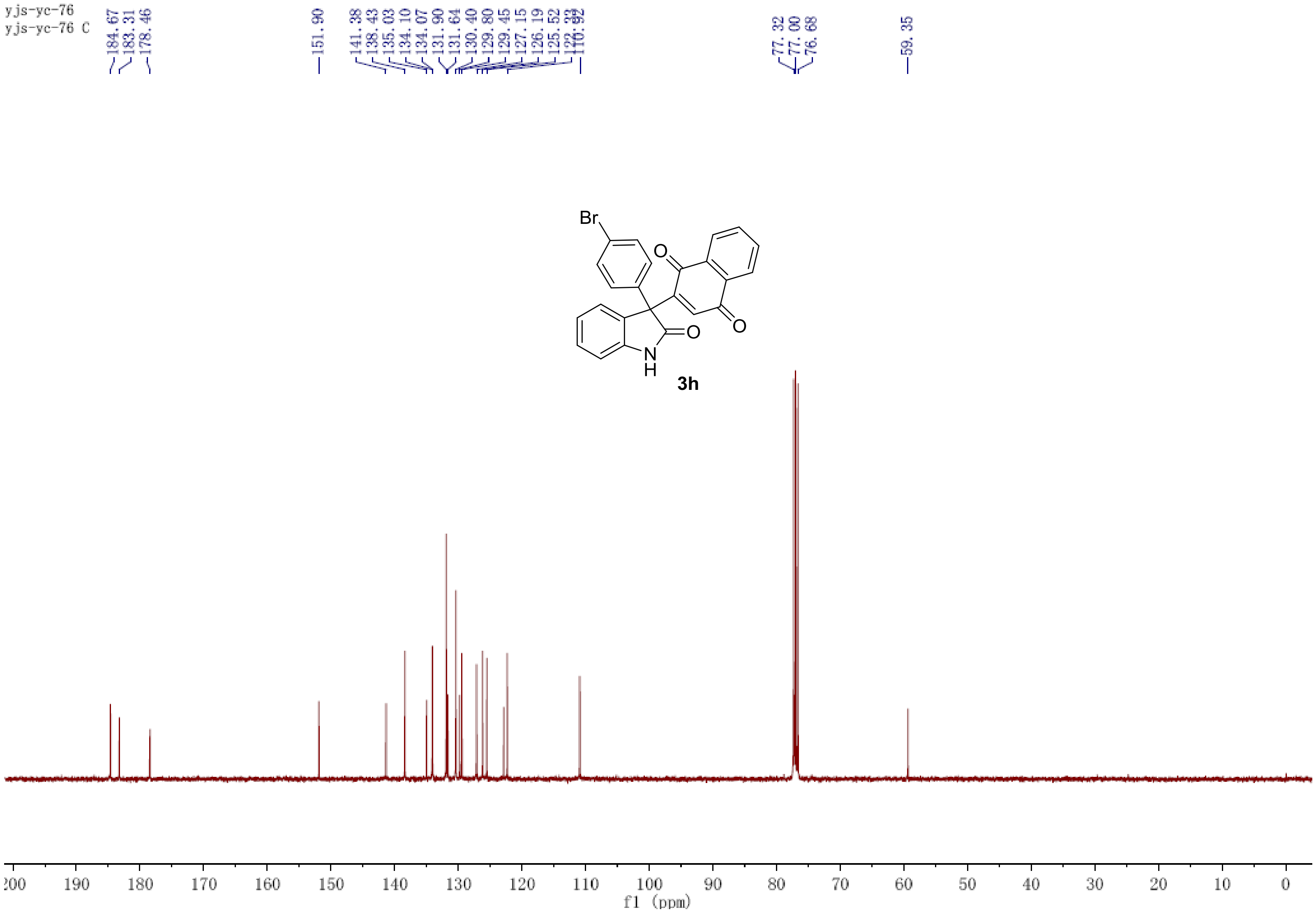
yjs-yc-76
yjs-yc-76 C
184.67
183.31
178.46
151.90
141.38
138.43
135.03
134.10
134.07
131.90
131.64
130.40
129.80
129.45
127.15
126.19
125.52
110.92
77.32
77.00
76.68
59.35
Br
O
O
O
N
H
3h
200 190 180 170 160 150 140 130 120 110 100 90 80 70 60 50 40 30 20 10 0
f1 (ppm)

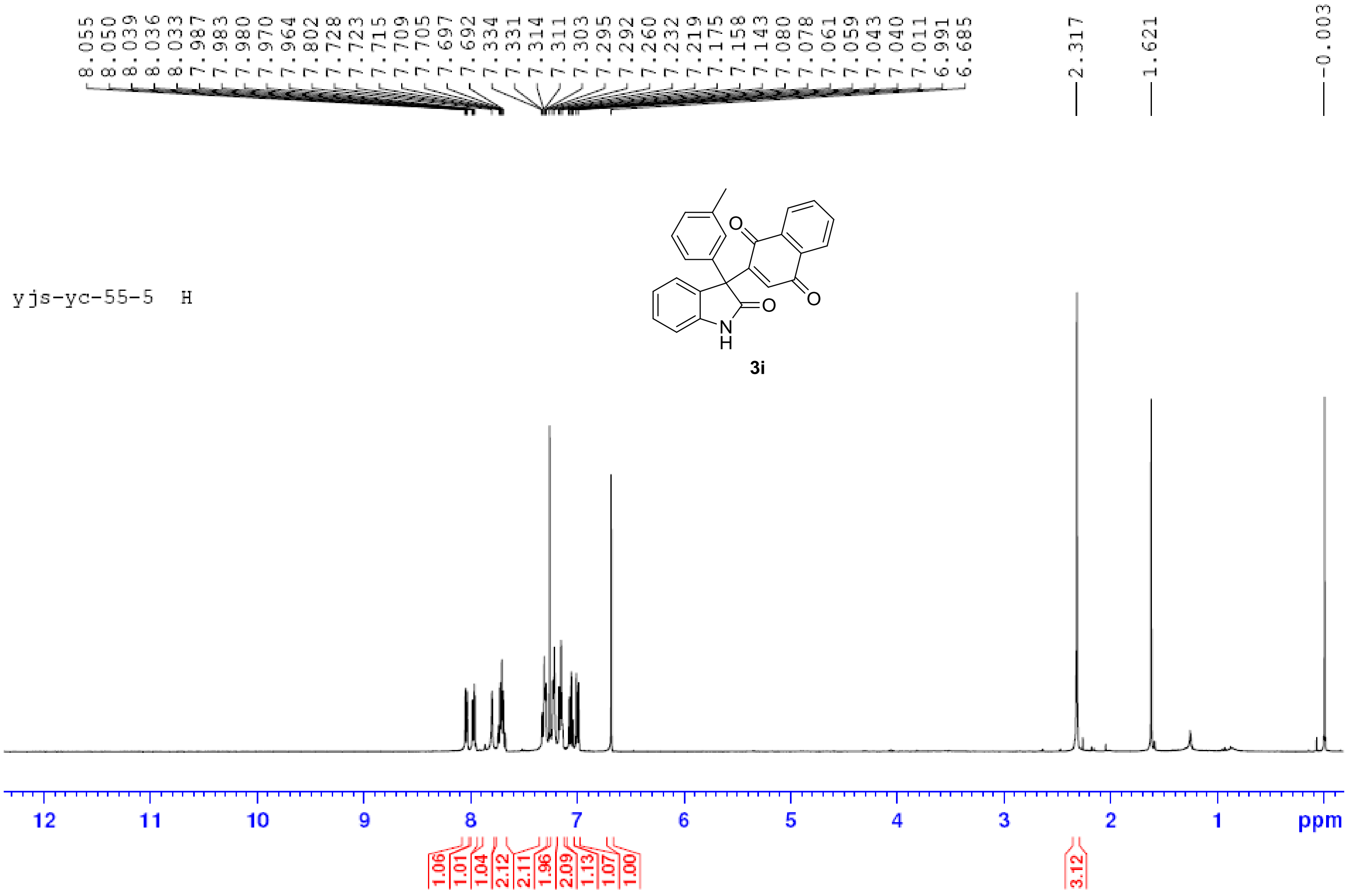
8.055
8.050
8.039
8.036
8.033
7.987
7.983
7.980
7.970
7.964
7.802
7.728
7.723
7.715
7.709
7.705
7.697
7.692
7.334
7.331
7.314
7.311
7.303
7.295
7.292
7.260
7.232
7.219
7.175
7.158
7.143
7.080
7.078
7.061
7.059
7.043
7.040
7.011
6.991
6.685
2.317
1.621
-0.003
yjs-yc-55-5 H
3i
12
11
10
9
8
7
6
5
4
3
2
1
ppm
1.06
1.01
1.04
2.12
2.11
1.96
2.09
1.13
1.07
1.00
3.12

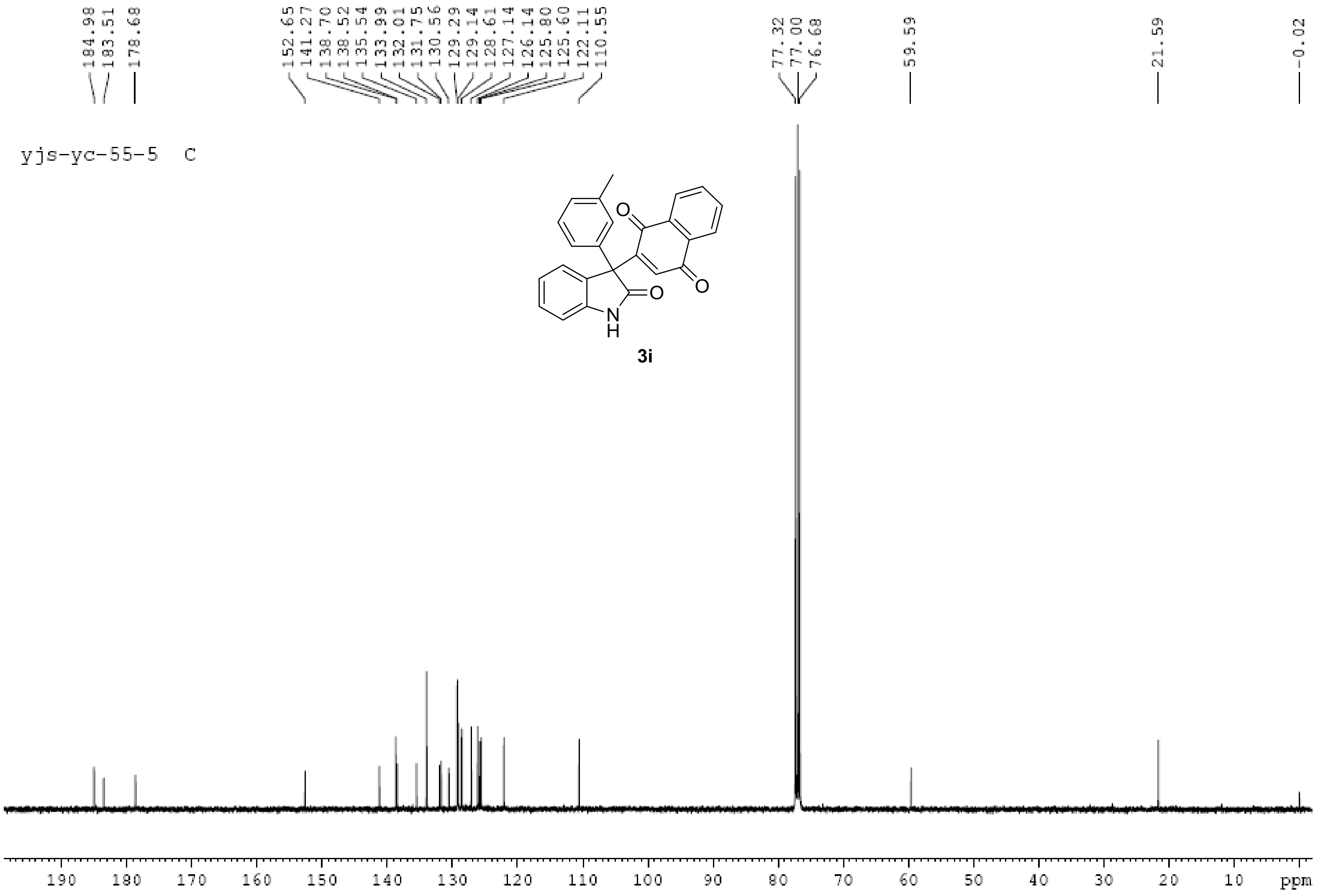
184.98
183.51
178.68
152.65
141.27
138.70
138.52
135.54
133.99
132.01
131.75
130.56
129.29
129.14
128.61
127.14
126.14
125.80
125.60
122.11
110.55
77.32
77.00
76.68
59.59
21.59
-0.02
yjs-yc-55-5 C
O
O
O
N
H
3i
190 180 170 160 150 140 130 120 110 100 90 80 70 60 50 40 30 20 10 ppm

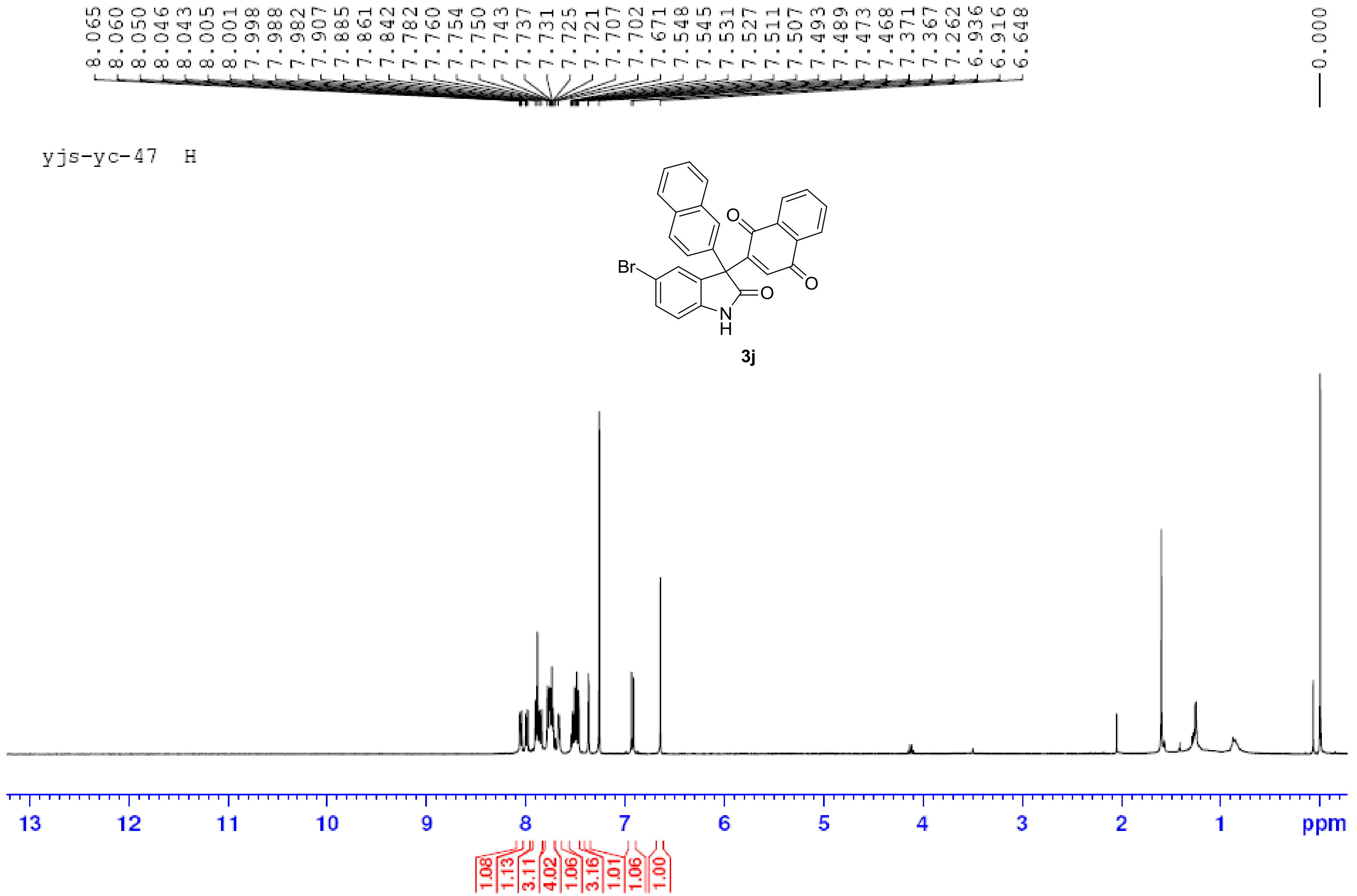
8.065 8.060 8.050 8.046 8.043 8.005 8.001 7.998 7.988 7.982 7.907 7.885 7.861 7.842 7.782 7.760 7.754 7.750 7.743 7.737 7.731 7.725 7.721 7.707 7.702 7.671 7.548 7.545 7.531 7.527 7.511 7.507 7.493 7.489 7.473 7.468 7.371 7.367 7.262 6.936 6.916 6.648
0.000
yjs-yc-47 H
Br
O
O
O
N
H
3j
13 12 11 10 9 8 7 6 5 4 3 2 1 ppm
1.08 1.13 3.11 4.02 1.06 3.16 1.01 1.06 1.00

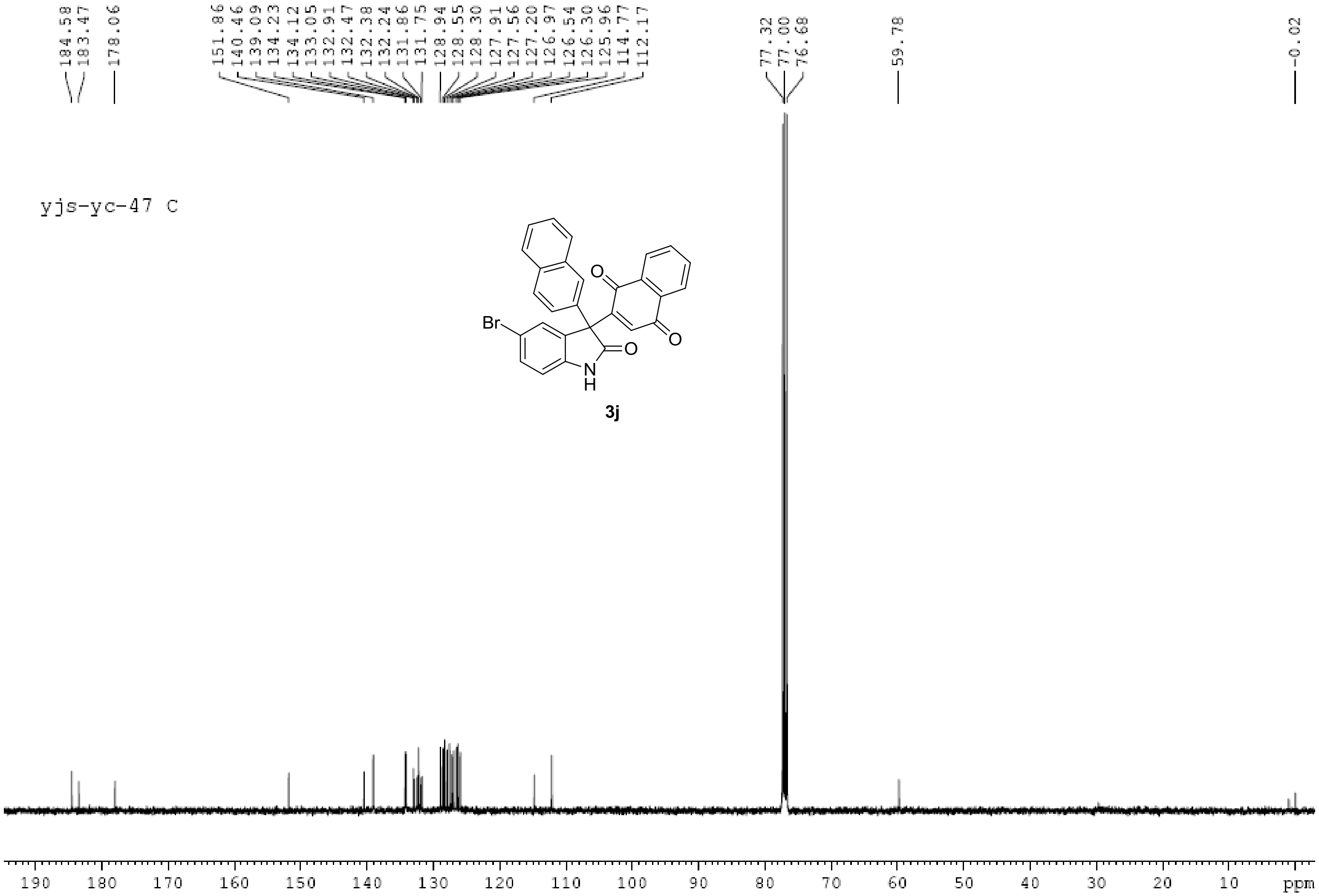
184.58
183.47
178.06
151.86
140.46
139.09
134.23
134.12
133.05
132.91
132.47
132.38
132.24
131.86
131.75
128.94
128.55
128.30
127.91
127.56
127.20
126.97
126.54
126.30
125.96
114.77
112.17
77.32
77.00
76.68
59.78
-0.02
yjs-yc-47 C
Br
O
O
O
N
H
3j
190
180
170
160
150
140
130
120
110
100
90
80
70
60
50
40
30
20
10
ppm

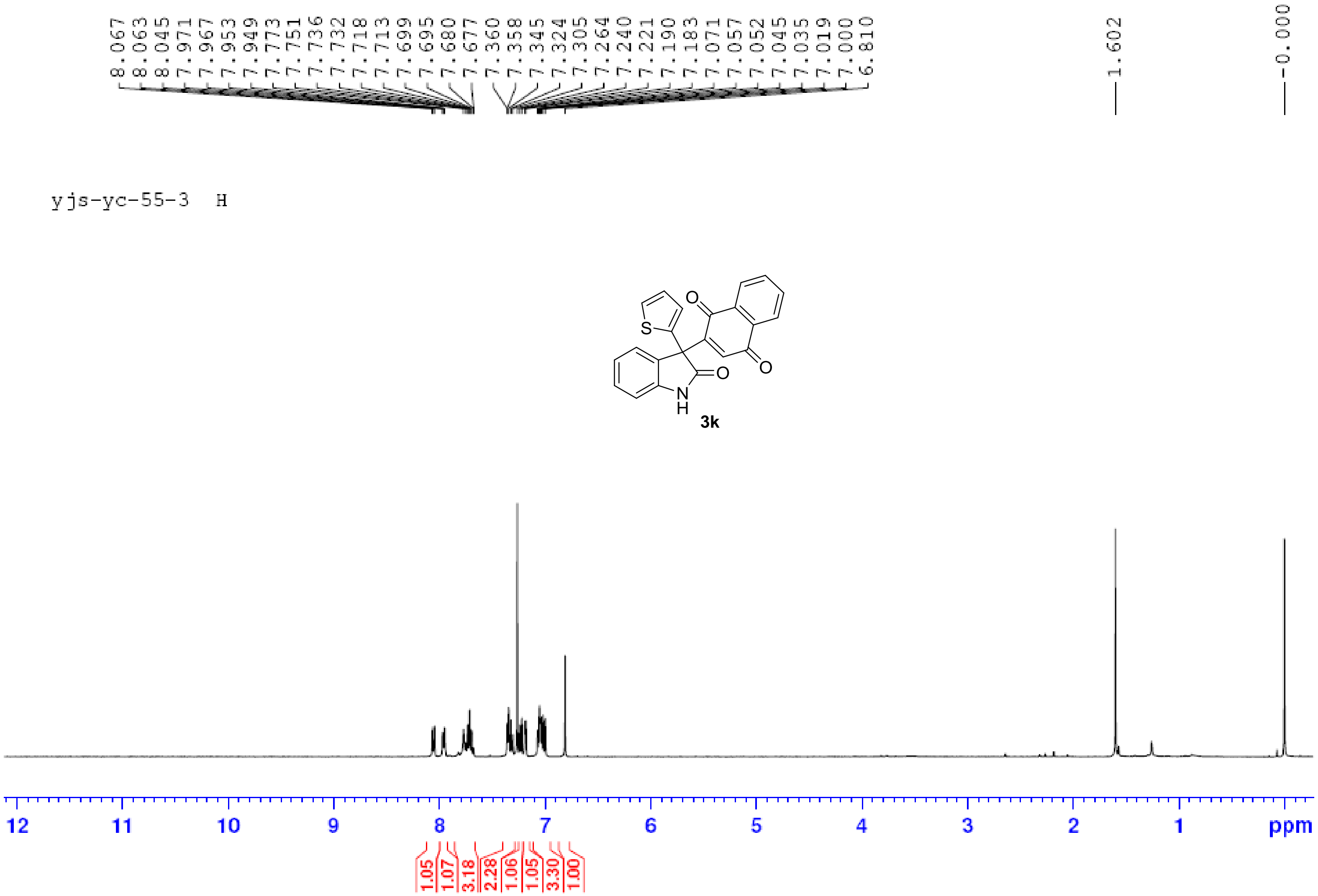
8.067
8.063
8.045
7.971
7.967
7.953
7.949
7.773
7.751
7.736
7.732
7.718
7.713
7.699
7.695
7.680
7.677
7.360
7.358
7.345
7.324
7.305
7.264
7.240
7.221
7.190
7.183
7.071
7.057
7.052
7.045
7.035
7.019
7.000
6.810
1.602
-0.000
yjs-yc-55-3 H
O
S
O
N
H
O
3k
12
11
10
9
8
7
6
5
4
3
2
1
ppm
1.05
1.07
3.18
2.28
1.06
1.05
3.30
1.00

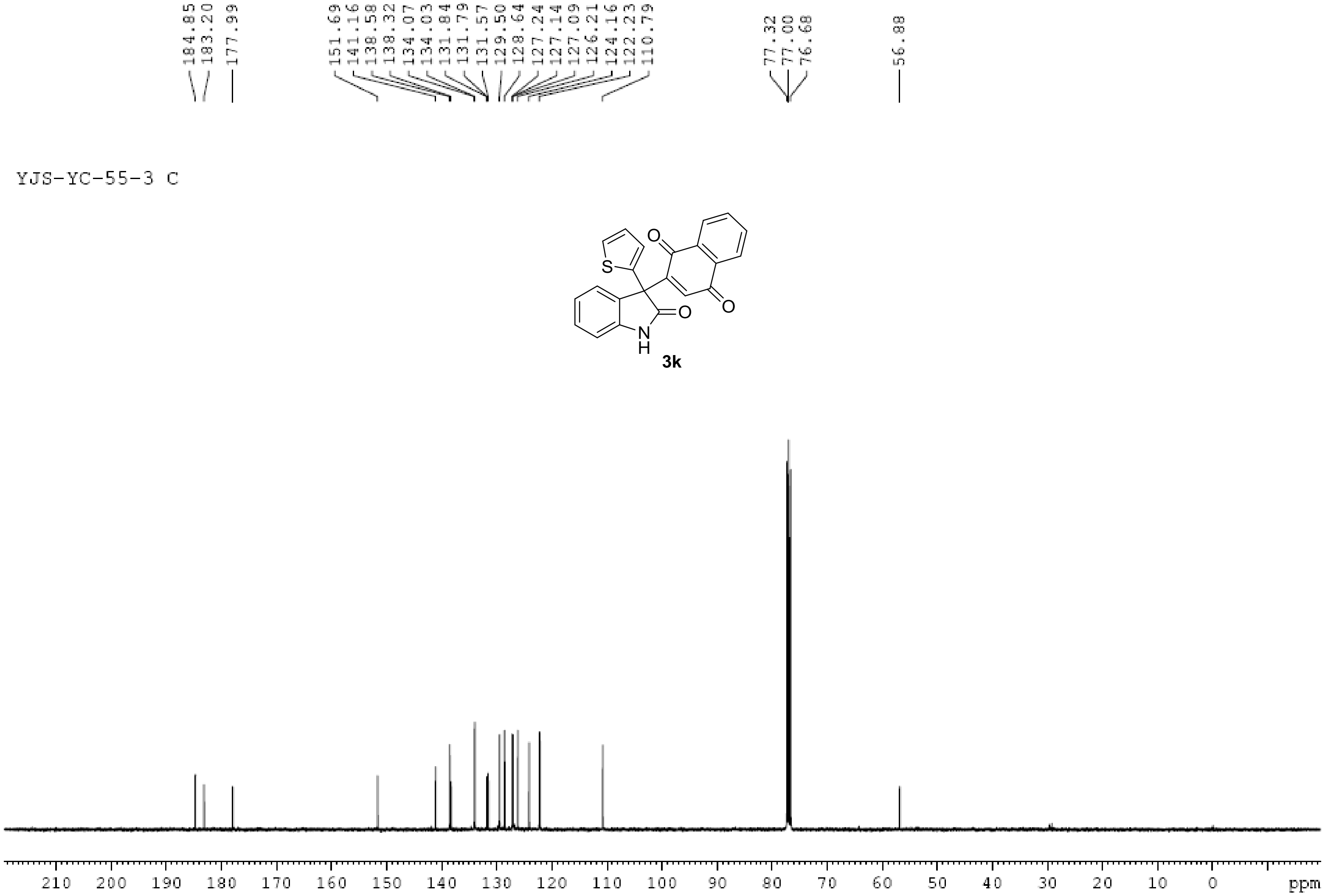
184.85
183.20
177.99
151.69
141.16
138.58
138.32
134.07
134.03
131.84
131.79
131.57
129.50
128.64
127.24
127.14
127.09
126.21
124.16
122.23
110.79
77.32
77.00
76.68
56.88
YJS-YC-55-3 C
S
O
O
O
N
H
3k
210 200 190 180 170 160 150 140 130 120 110 100 90 80 70 60 50 40 30 20 10 0 ppm

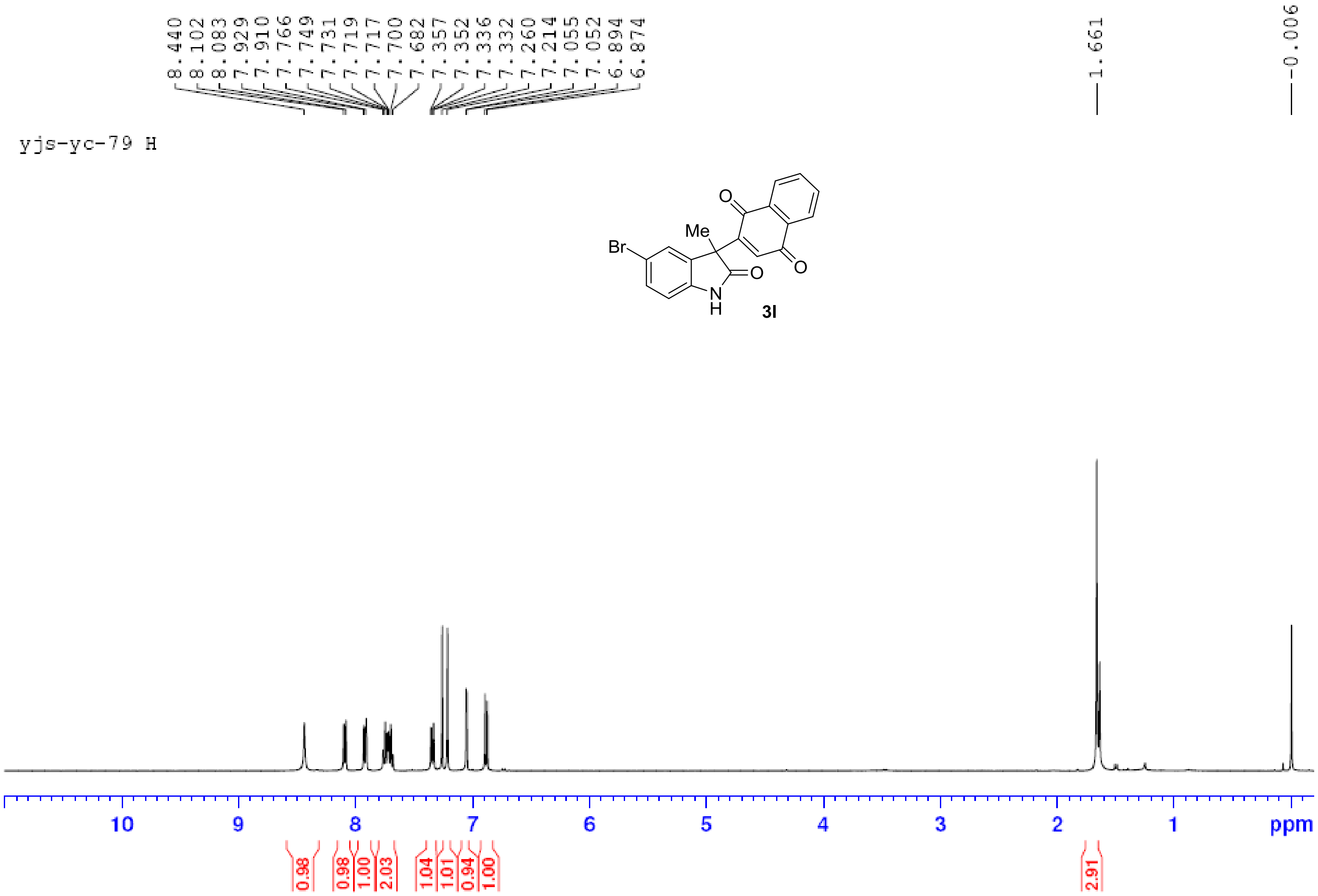
8.440
8.102
8.083
7.929
7.910
7.766
7.749
7.731
7.719
7.717
7.700
7.682
7.357
7.352
7.336
7.332
7.260
7.214
7.055
7.052
6.894
6.874
1.661
-0.006
yjs-yc-79 H
Br
Me
O
O
O
N
H
3l
10
9
8
7
6
5
4
3
2
1
ppm
0.98
0.98
1.00
2.03
1.04
1.01
0.94
1.00
2.91

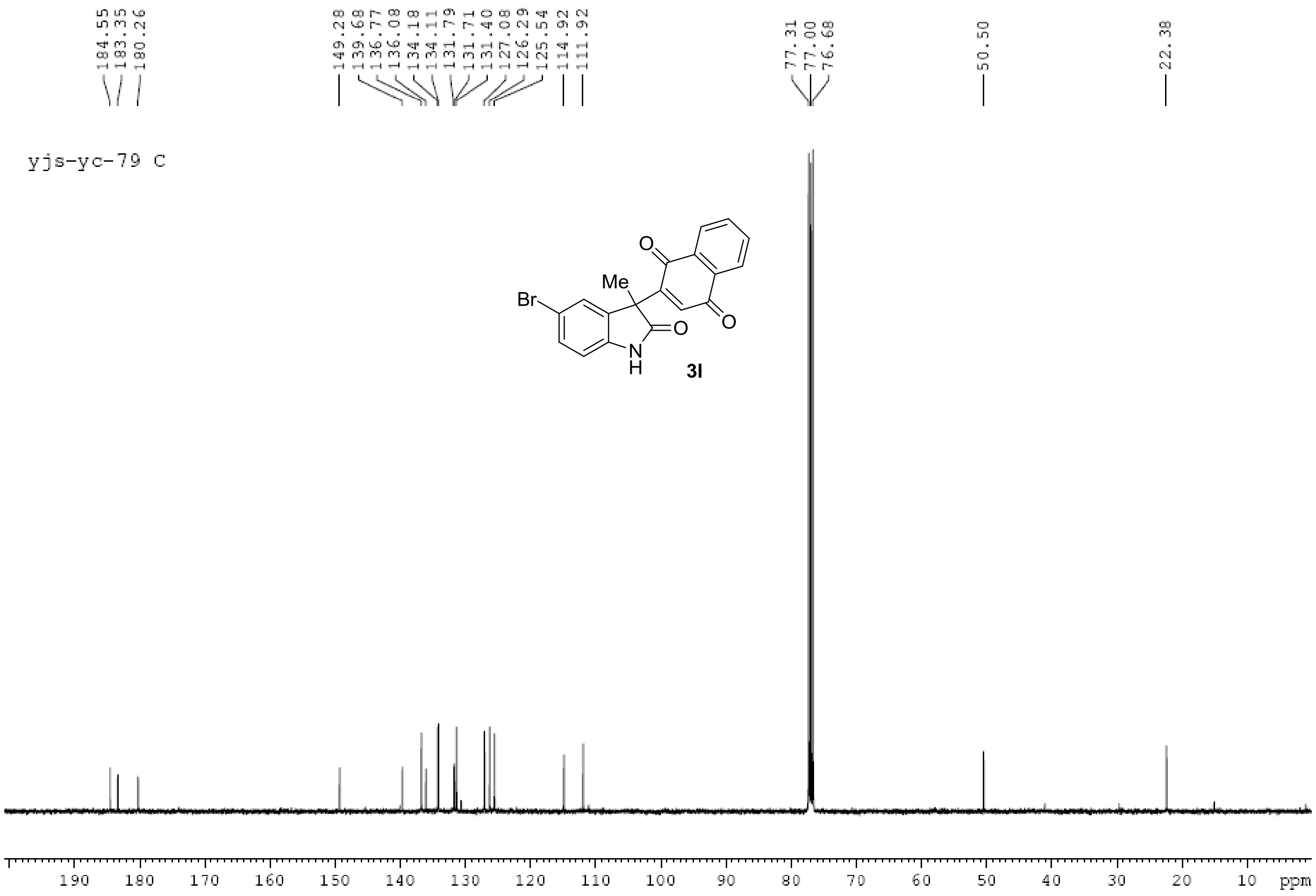
184.55
183.35
180.26
149.28
139.68
136.77
136.08
134.18
134.11
131.79
131.71
131.40
127.08
126.29
125.54
114.92
111.92
77.31
77.00
76.68
50.50
22.38
yjs-yc-79 C
Br
Me
O
NH
3l
190 180 170 160 150 140 130 120 110 100 90 80 70 60 50 40 30 20 10 ppm

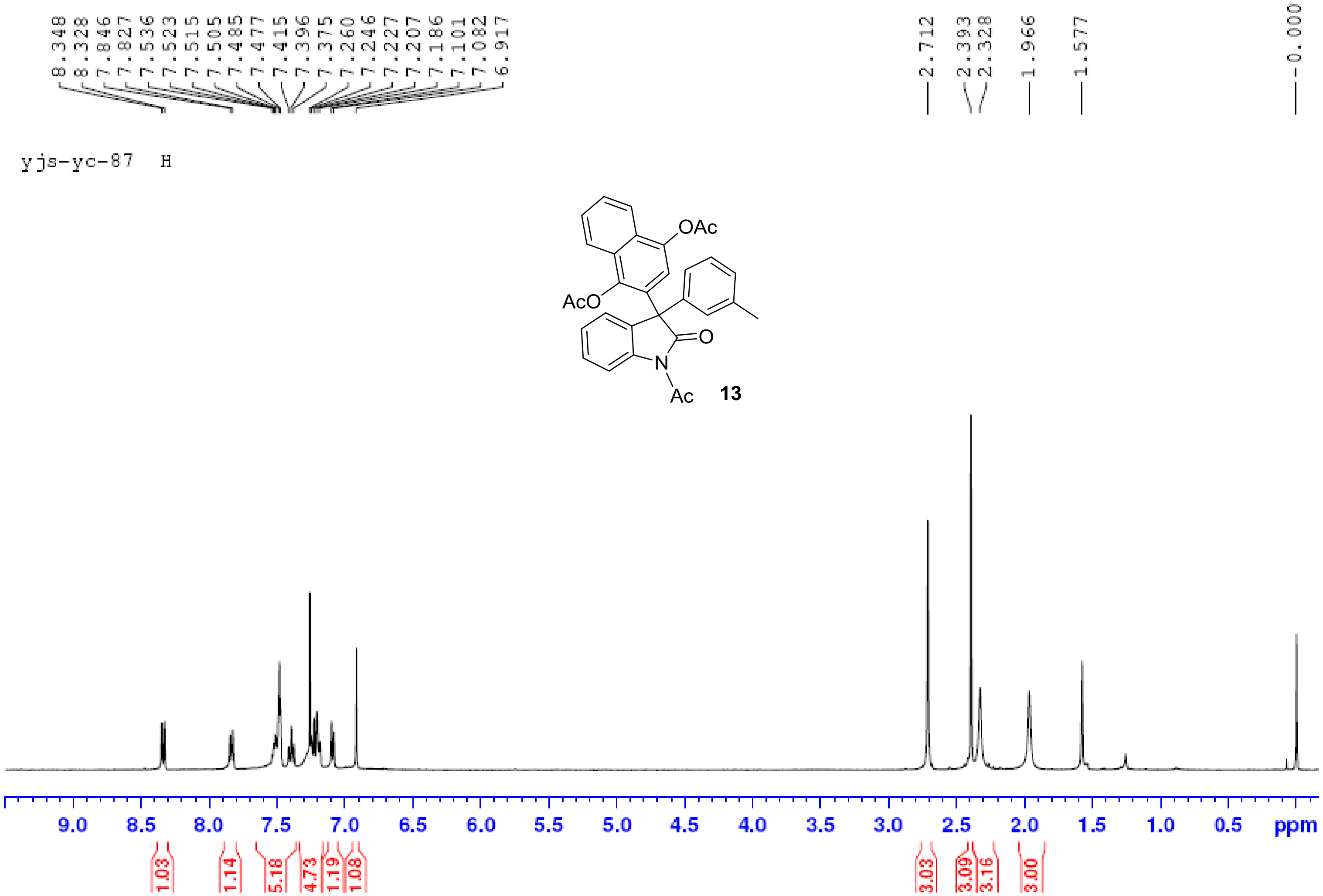
8.348 8.328 7.846 7.827 7.536 7.523 7.515 7.505 7.485 7.477 7.415 7.396 7.375 7.260 7.246 7.227 7.207 7.186 7.101 7.082 6.917
2.712 2.393 2.328 1.966 1.577
0.000
yjs-yc-87 H
OAc
AcO
O
N
Ac
13
9.0 8.5 8.0 7.5 7.0 6.5 6.0 5.5 5.0 4.5 4.0 3.5 3.0 2.5 2.0 1.5 1.0 0.5 ppm
1.03 1.14 5.18 4.73 1.19 1.08 3.03 3.09 3.16 3.00

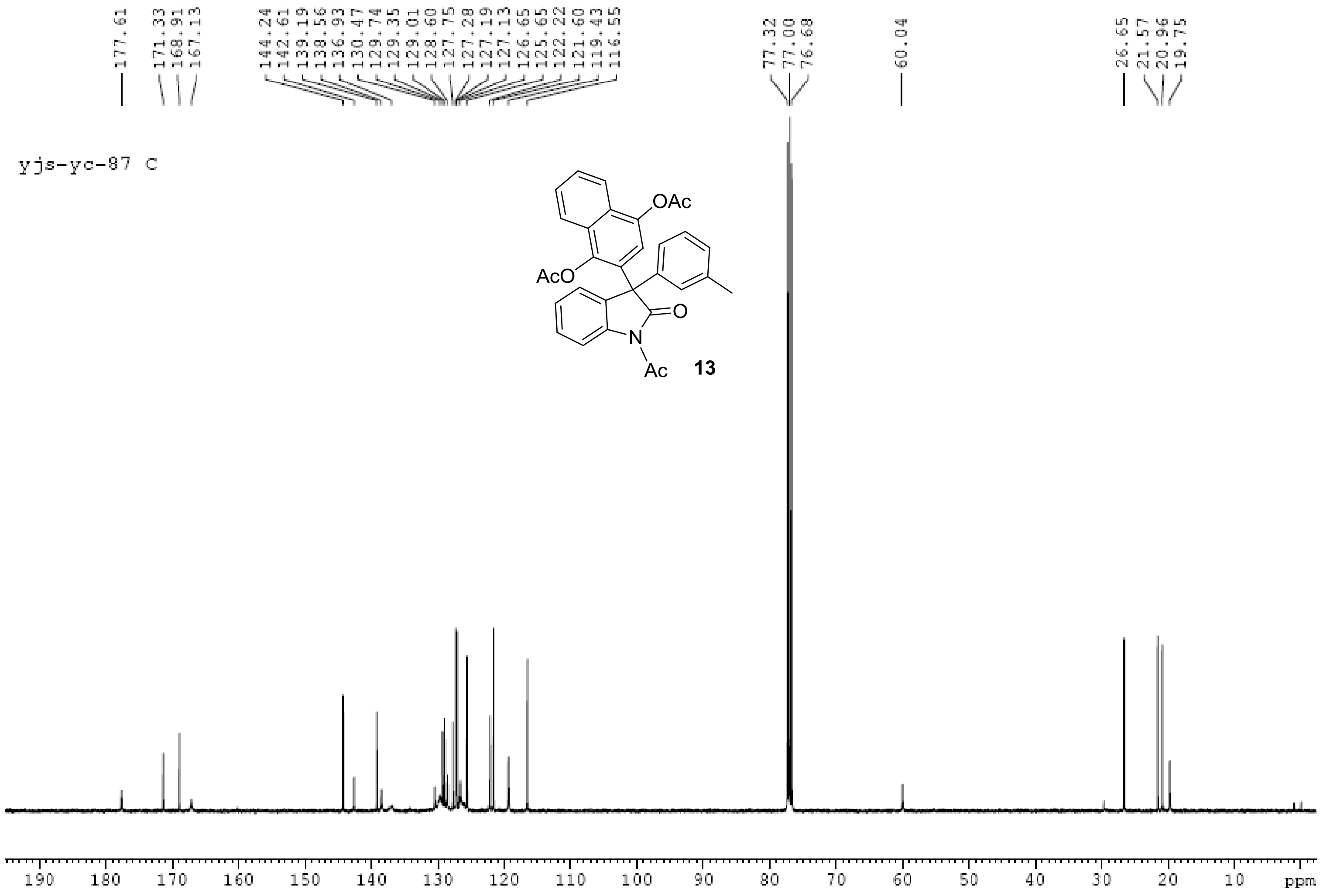

yjs-yc-87 C
177.61
171.33
168.91
167.13
144.24
142.61
139.19
138.56
136.93
130.47
129.74
129.35
129.01
128.60
127.75
127.28
127.19
127.13
126.65
125.65
122.22
121.60
119.43
116.55
77.32
77.00
76.68
60.04
26.65
21.57
20.96
19.75
OAc
AcO
O
N
Ac
13
190 180 170 160 150 140 130 120 110 100 90 80 70 60 50 40 30 20 10 ppm

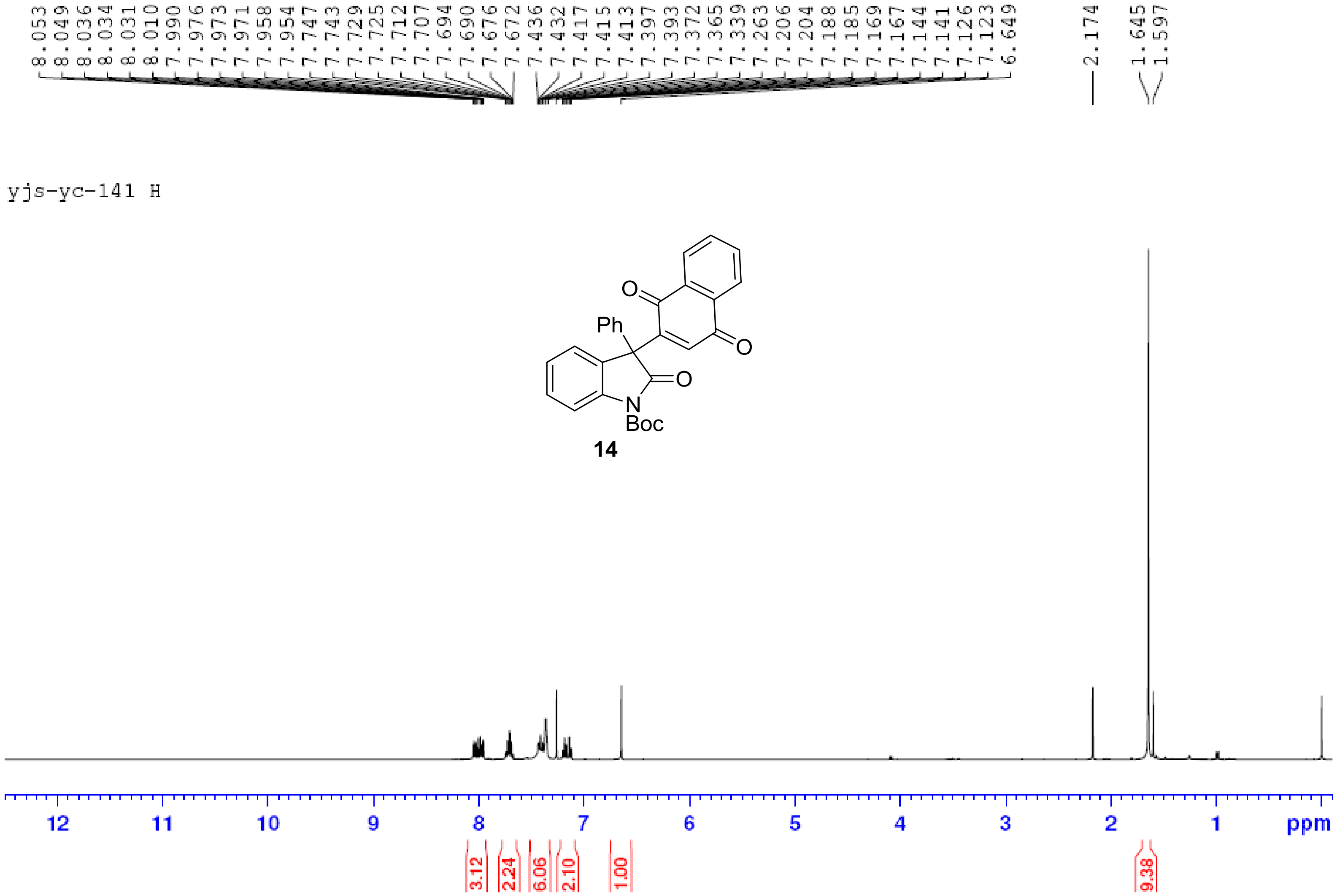

yjs-yc-141 H
Ph
O
O
O
N
Boc
14
ppm

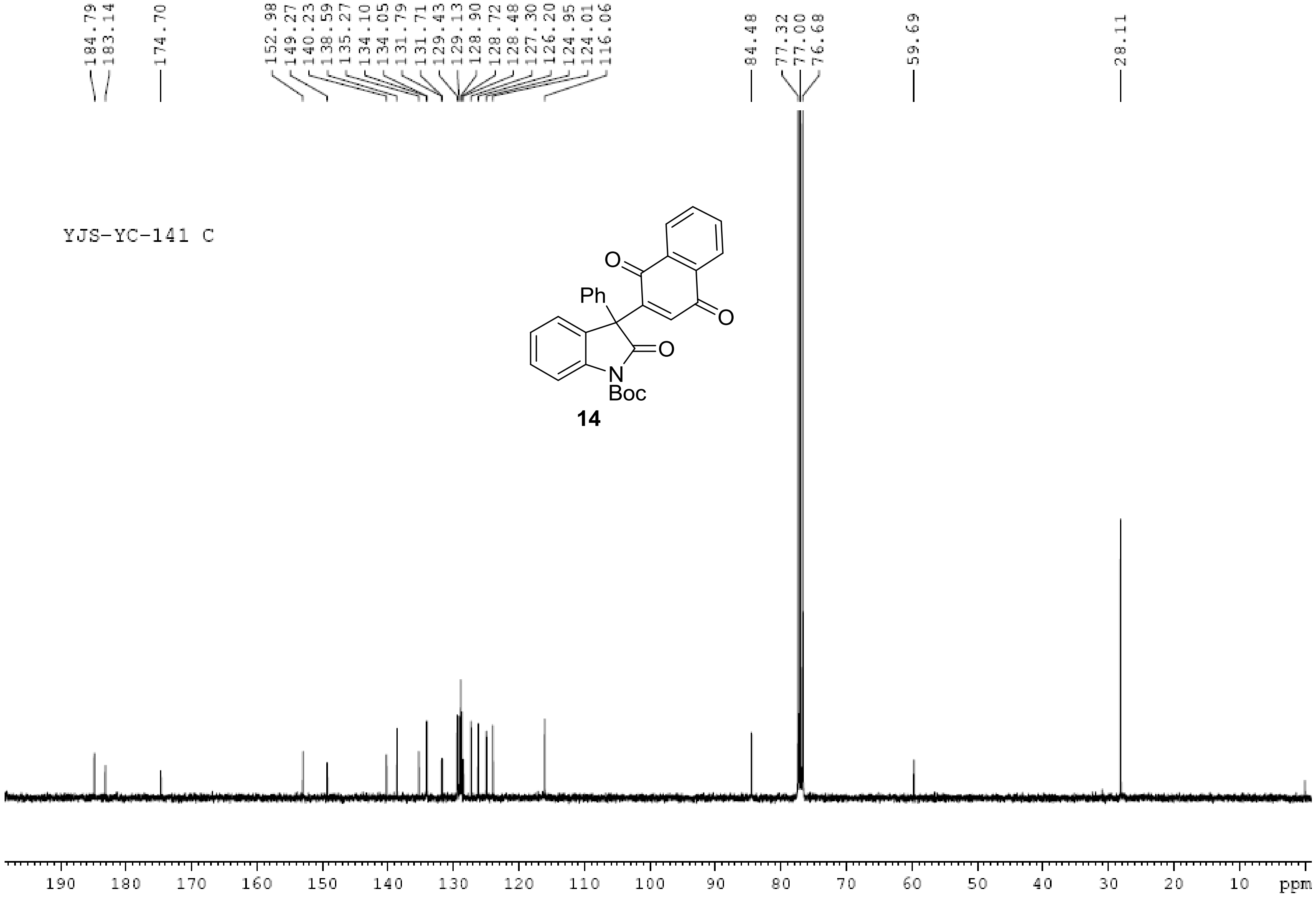

184.79
183.14
174.70
152.98
149.27
140.23
138.59
135.27
134.10
134.05
131.79
131.71
129.43
129.13
128.90
128.72
128.48
127.30
126.20
124.95
124.01
116.06
84.48
77.32
77.00
76.68
59.69
28.11
YJS-YC-141 C
O
Ph
O
O
N
Boc
14
190
180
170
160
150
140
130
120
110
100
90
80
70
60
50
40
30
20
10
ppm

Operator:dell Timebase:U-3000 Sequence:YJS

Pag
2012-5-23 3:37

339 yjs-yb-24-Br-ODH-rac-80/20-230-1.0-1

Sample Name:	**yjs-yb-24-Br-ODH-rac-80/20-230-1.0-1**	*Injection Volume:*	**20.0**
Vial Number:	**668**	*Channel:*	**UV_VIS_1**
Sample Type:	**standard**	*Wavelength:*	**230**
Control Program:	**YJS**	*Bandwidth:*	**n.a.**
Quantif. Method:	**YJS**	*Dilution Factor:*	**1.0000**
Recording Time:	**2012-5-23 13:31**	*Sample Weight:*	**1.0000**
Run Time (min):	**32.76**	*Sample Amount:*	**1.0000**

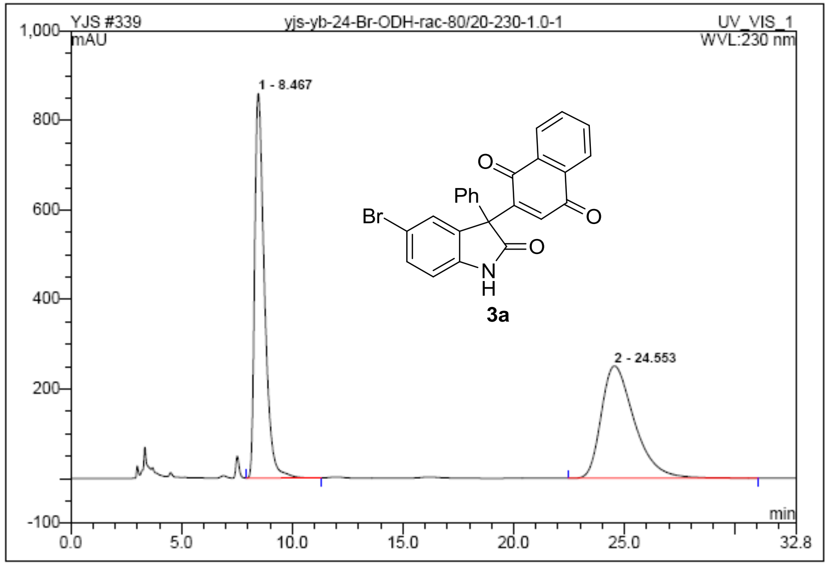

No.	Ret.Time min	Peak Name	Height mAU	Area mAU*min	Rel.Area %	Amount	Type
1	8.47	n.a.	859.086	439.318	50.12	n.a.	BMB
2	24.55	n.a.	251.030	437.181	49.88	n.a.	BMB
Total:			1110.116	876.500	100.00	0.000	

Operator:dell Timebase:U-3000 Sequence:YJS

Pag
2012-5-20 5:07

227 YJS-YB-142-ODH-ASY-80/20-230-1.0-1

Sample Name:	**YJS-YB-142-ODH-ASY-80/20-230-1.0-1**	*Injection Volume:*	**20.0**
Vial Number:	**552**	*Channel:*	**UV_VIS_1**
Sample Type:	**standard**	*Wavelength:*	**230**
Control Program:	**YJS**	*Bandwidth:*	**n.a.**
Quantif. Method:	**YJS**	*Dilution Factor:*	**1.0000**
Recording Time:	**2012-4-3 18:48**	*Sample Weight:*	**1.0000**
Run Time (min):	**34.65**	*Sample Amount:*	**1.0000**

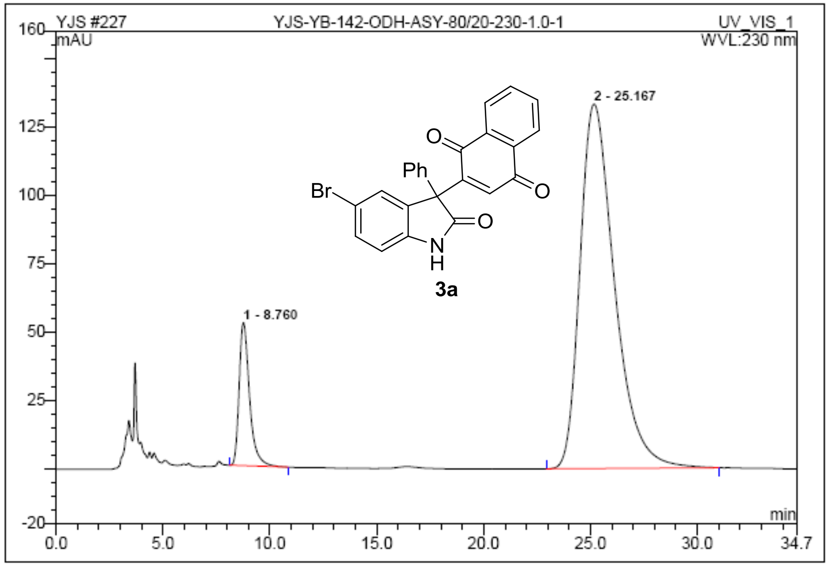

No.	Ret.Time min	Peak Name	Height mAU	Area mAU*min	Rel.Area %	Amount	Type
1	8.76	n.a.	52.253	29.519	10.59	n.a.	BMB
2	25.17	n.a.	133.058	249.125	89.41	n.a.	BMB
Total:			185.311	278.645	100.00	0.000	

Operator:dell Timebase:U-3000 Sequence:YJS

Pa[illegible]
2012-5-23 12:5[illegible]

234 YJS-YB-139-3-ODH-RAC-80/20-230-1.0-1

Sample Name:	**YJS-YB-139-3-ODH-RAC-80/20-230-1.0-1**	*Injection Volume:*	**20.0**
Vial Number:	**559**	*Channel:*	**UV_VIS_1**
Sample Type:	**standard**	*Wavelength:*	**230**
Control Program:	**YJS**	*Bandwidth:*	**n.a.**
Quantif. Method:	**YJS**	*Dilution Factor:*	**1.0000**
Recording Time:	**2012-4-6 13:06**	*Sample Weight:*	**1.0000**
Run Time (min):	**27.58**	*Sample Amount:*	**1.0000**

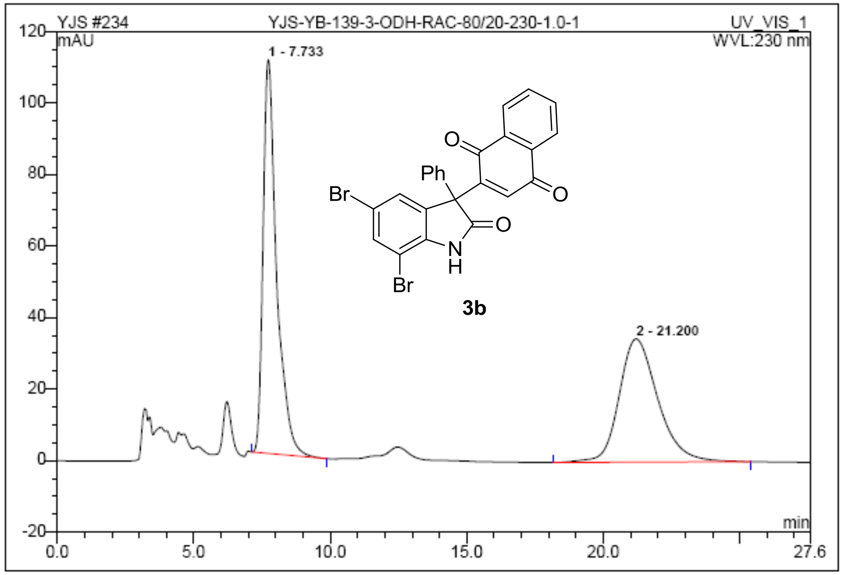

No.	Ret.Time min	Peak Name	Height mAU	Area mAU*min	Rel.Area %	Amount	Type
1	7.73	n.a.	110.100	63.136	52.64	n.a.	BMB
2	21.20	n.a.	34.464	56.808	47.36	n.a.	BMB
Total:			144.564	119.944	100.00	0.000	

Operator:dell Timebase:U-3000 Sequence:YJS

Pag[illegible]
2012-5-20 5:22

296 YJS-YC-55-1-ODH-ASY-80/20-230-1.0-1

Sample Name:	**YJS-YC-55-1-ODH-ASY-80/20-230-1.0-1**	*Injection Volume:*	**20.0**
Vial Number:	**624**	*Channel:*	**UV_VIS_1**
Sample Type:	**standard**	*Wavelength:*	**230**
Control Program:	**YJS**	*Bandwidth:*	**n.a.**
Quantif. Method:	**YJS**	*Dilution Factor:*	**1.0000**
Recording Time:	**2012-5-4 16:00**	*Sample Weight:*	**1.0000**
Run Time (min):	**28.78**	*Sample Amount:*	**1.0000**

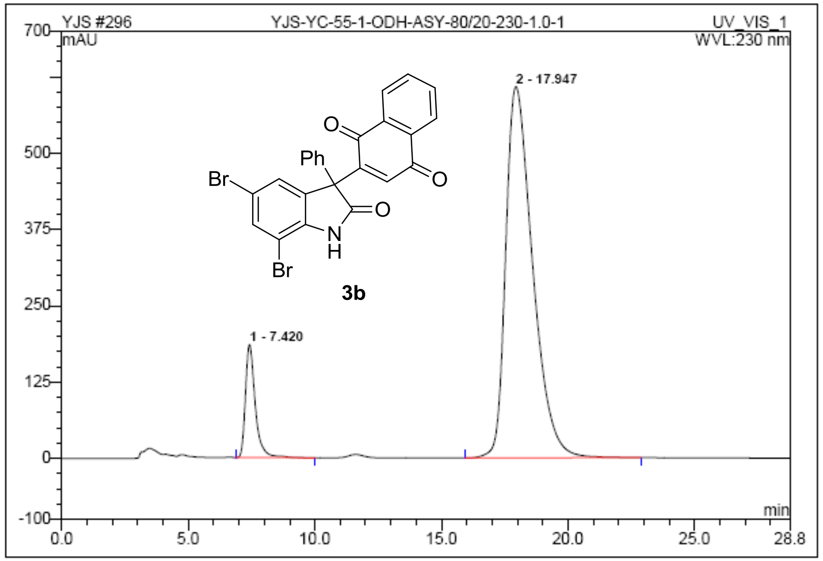

No.	Ret.Time min	Peak Name	Height mAU	Area mAU*min	Rel.Area %	Amount	Type
1	7.42	n.a.	185.168	83.590	10.05	n.a.	BMB
2	17.95	n.a.	607.707	748.037	89.95	n.a.	BMB
Total:			792.875	831.628	100.00	0.000	

Operator:dell Timebase:U-3000 Sequence:YJS

Pag[illegible]
2012-5-23 3:3[illegible]

338 yjs-yb-24-cl-ODH-RAC-80/20-254-1.0-1

Sample Name:	yjs-yb-24-cl-ODH-RAC-80/20-254-1.0-1	*Injection Volume:*	20.0
Vial Number:	666	*Channel:*	UV_VIS_1
Sample Type:	standard	*Wavelength:*	254
Control Program:	YJS	*Bandwidth:*	n.a.
Quantif. Method:	YJS	*Dilution Factor:*	1.0000
Recording Time:	2012-5-23 12:12	*Sample Weight:*	1.0000
Run Time (min):	33.24	*Sample Amount:*	1.0000

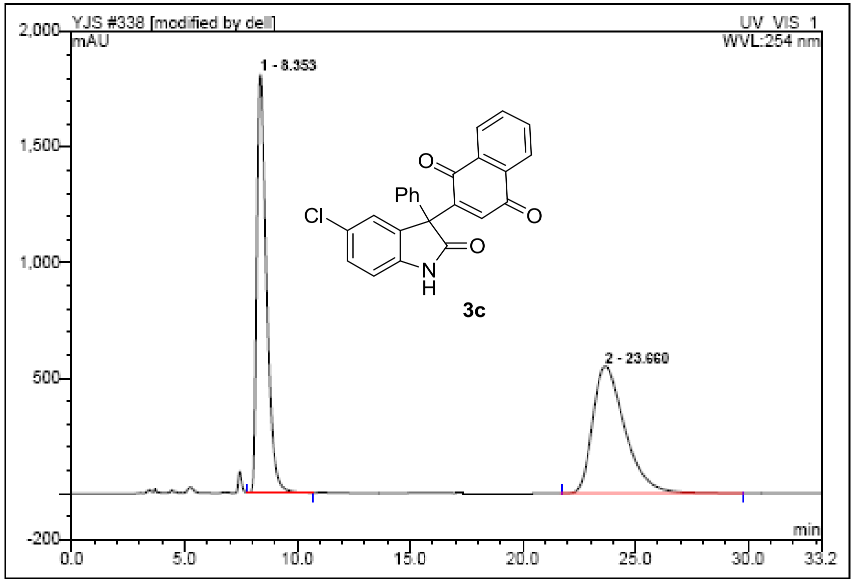

No.	Ret.Time min	Peak Name	Height mAU	Area mAU*min	Rel.Area %	Amount	Type
1	8.35	n.a.	1810.885	904.508	49.71	n.a.	MB*
2	23.66	n.a.	550.209	914.951	50.29	n.a.	BMB
Total:			2361.094	1819.459	100.00	0.000	

Operator:dell Timebase:U-3000 Sequence:YJS

Pag
2012-5-20 7:02

314 YJS-YC-55-2-ODH-ASY-80/20-254-1.0-1

Sample Name:	YJS-YC-55-2-ODH-ASY-80/20-254-1.0-1	*Injection Volume:*	20.0
Vial Number:	642	*Channel:*	UV_VIS_1
Sample Type:	standard	*Wavelength:*	254
Control Program:	YJS	*Bandwidth:*	n.a.
Quantif. Method:	YJS	*Dilution Factor:*	1.0000
Recording Time:	2012-5-9 12:09	*Sample Weight:*	1.0000
Run Time (min):	36.20	*Sample Amount:*	1.0000

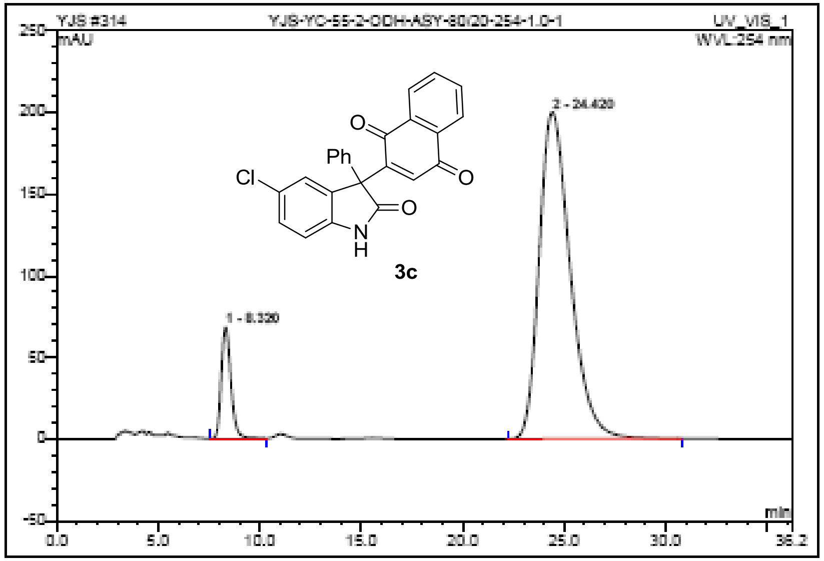

No.	Ret.Time min	Peak Name	Height mAU	Area mAU*min	Rel.Area %	Amount	Type
1	8.32	n.a.	67.893	37.694	9.58	n.a.	BMB
2	24.42	n.a.	200.217	355.695	90.42	n.a.	BMB
Total:			268.110	393.388	100.00	0.000	

Operator:dell Timebase:U-3000 Sequence:YJS

Pa
2012-5-20 7:(

278 YJS-YB-24-F-ODH-rac-80/20-230-1.0-1

Sample Name:	**YJS-YB-24-F-ODH-rac-80/20-230-1.0-1**	*Injection Volume:*	**20.0**
Vial Number:	**605**	*Channel:*	**UV_VIS_1**
Sample Type:	**standard**	*Wavelength:*	**230**
Control Program:	**YJS**	*Bandwidth:*	**n.a.**
Quantif. Method:	**YJS**	*Dilution Factor:*	**1.0000**
Recording Time:	**2012-4-22 13:02**	*Sample Weight:*	**1.0000**
Run Time (min):	**34.75**	*Sample Amount:*	**1.0000**

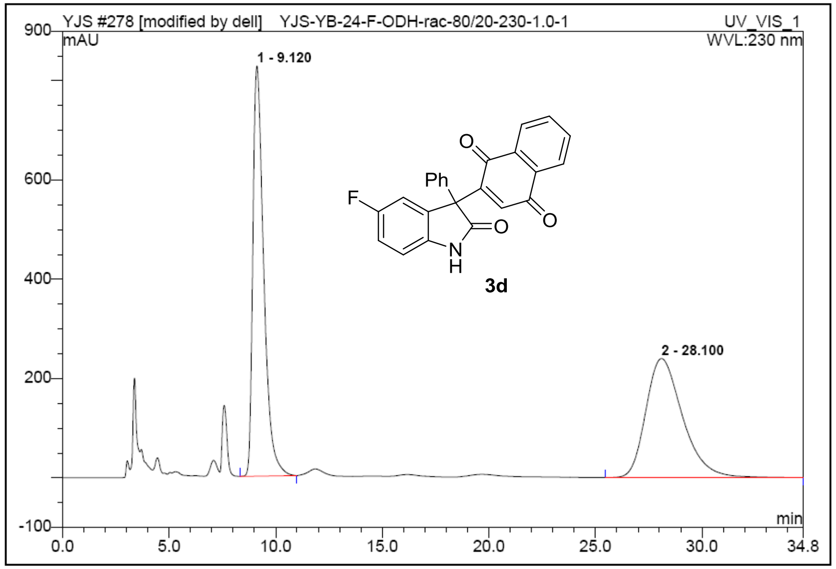

No.	Ret.Time min	Peak Name	Height mAU	Area mAU*min	Rel.Area %	Amount	Type
1	9.12	n.a.	826.661	488.099	50.52	n.a.	BMB*
2	28.10	n.a.	239.696	478.028	49.48	n.a.	BMB
**Total:**			1066.357	966.127	100.00	0.000	

Operator:dell Timebase:U-3000 Sequence:YJS

Pag
2012-5-20 5:29

327 YJS-YC-75-ODH-ASY-80/20-230-1.0-1

Sample Name:	**YJS-YC-75-ODH-ASY-80/20-230-1.0-1**	*Injection Volume:*	**20.0**
Vial Number:	**655**	*Channel:*	**UV_VIS_1**
Sample Type:	**standard**	*Wavelength:*	**230**
Control Program:	**YJS**	*Bandwidth:*	**n.a.**
Quantif. Method:	**YJS**	*Dilution Factor:*	**1.0000**
Recording Time:	**2012-5-16 17:42**	*Sample Weight:*	**1.0000**
Run Time (min):	**43.02**	*Sample Amount:*	**1.0000**

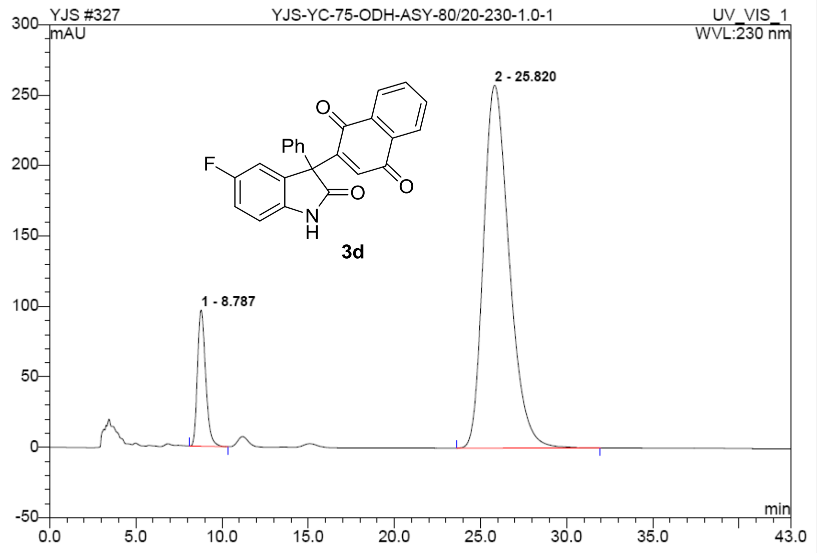

No.	Ret.Time min	Peak Name	Height mAU	Area mAU*min	Rel.Area %	Amount	Type
1	8.79	n.a.	96.623	53.270	10.68	n.a.	BMB
2	25.82	n.a.	257.477	445.444	89.32	n.a.	BMB
**Total:**			354.100	498.714	100.00	0.000	

Operator:dell Timebase:U-3000 Sequence:YJS

Pa
2012-5-20 7:1

231 YJS-YB-135-4-ODH-ASY-80/20-230-1.0-1

Sample Name:	**YJS-YB-135-4-ODH-ASY-80/20-230-1.0-1**	*Injection Volume:*	**20.0**
Vial Number:	**556**	*Channel:*	**UV_VIS_1**
Sample Type:	**standard**	*Wavelength:*	**230**
Control Program:	**YJS**	*Bandwidth:*	**n.a.**
Quantif. Method:	**YJS**	*Dilution Factor:*	**1.0000**
Recording Time:	**2012-4-6 11:31**	*Sample Weight:*	**1.0000**
Run Time (min):	**29.70**	*Sample Amount:*	**1.0000**

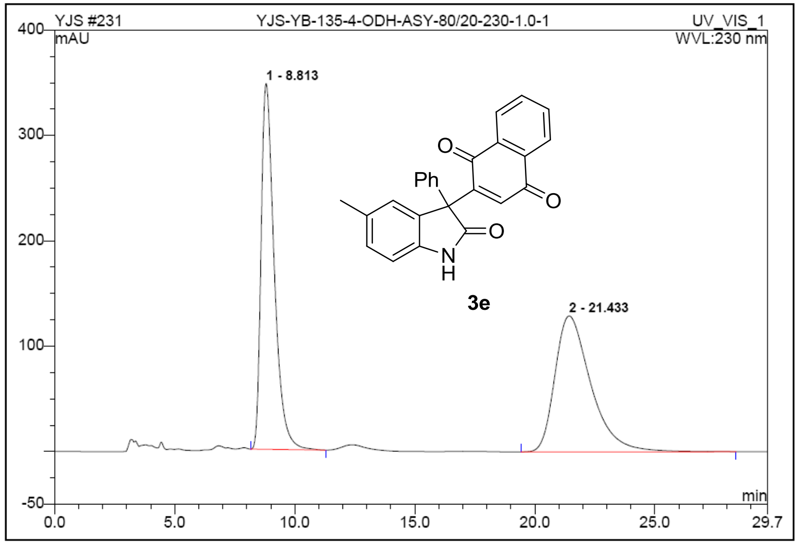

No.	Ret.Time min	Peak Name	Height mAU	Area mAU*min	Rel.Area %	Amount	Type
1	8.81	n.a.	347.033	220.667	49.63	n.a.	BMB
2	21.43	n.a.	128.954	223.939	50.37	n.a.	BMB
**Total:**			475.987	444.606	100.00	0.000	

Operator:dell Timebase:U-3000 Sequence:YJS

Pag
2012-5-20 7:09

317 YJS-YC-63-ODH-ASY-80/20-230-1.0-1

Sample Name:	**YJS-YC-63-ODH-ASY-80/20-230-1.0-1**	*Injection Volume:*	**20.0**
Vial Number:	**645**	*Channel:*	**UV_VIS_1**
Sample Type:	**standard**	*Wavelength:*	**230**
Control Program:	**YJS**	*Bandwidth:*	**n.a.**
Quantif. Method:	**YJS**	*Dilution Factor:*	**1.0000**
Recording Time:	**2012-5-12 15:31**	*Sample Weight:*	**1.0000**
Run Time (min):	**27.98**	*Sample Amount:*	**1.0000**

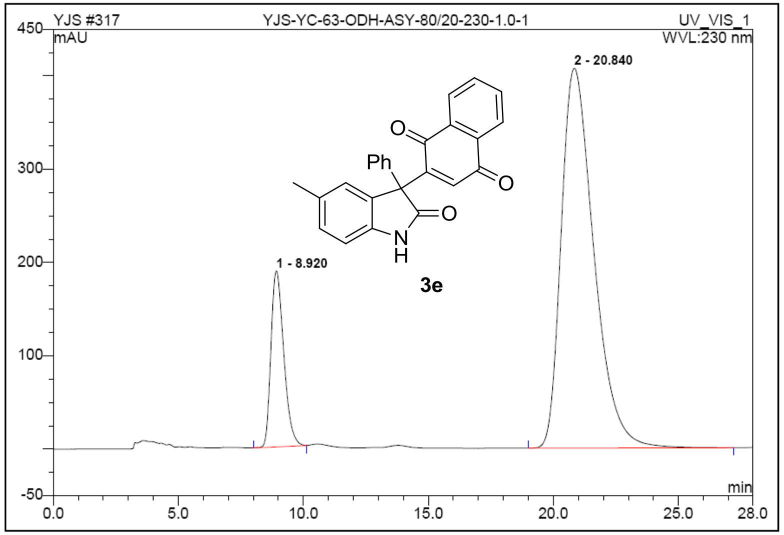

No.	Ret.Time min	Peak Name	Height mAU	Area mAU*min	Rel.Area %	Amount	Type
1	8.92	n.a.	188.580	111.780	15.34	n.a.	BMB
2	20.84	n.a.	407.205	616.910	84.66	n.a.	BMB
**Total:**			595.785	728.690	100.00	0.000	

294 YJS-YC-27-3-ODH-rac-80/20-230-1.0-1

Sample Name:	**YJS-YC-27-3-ODH-rac-80/20-230-1.0-1**	*Injection Volume:*	**20.0**
Vial Number:	**621**	*Channel:*	**UV_VIS_1**
Sample Type:	**standard**	*Wavelength:*	**230**
Control Program:	**YJS**	*Bandwidth:*	**n.a.**
Quantif. Method:	**YJS**	*Dilution Factor:*	**1.0000**
Recording Time:	**2012-5-2 19:44**	*Sample Weight:*	**1.0000**
Run Time (min):	**39.51**	*Sample Amount:*	**1.0000**

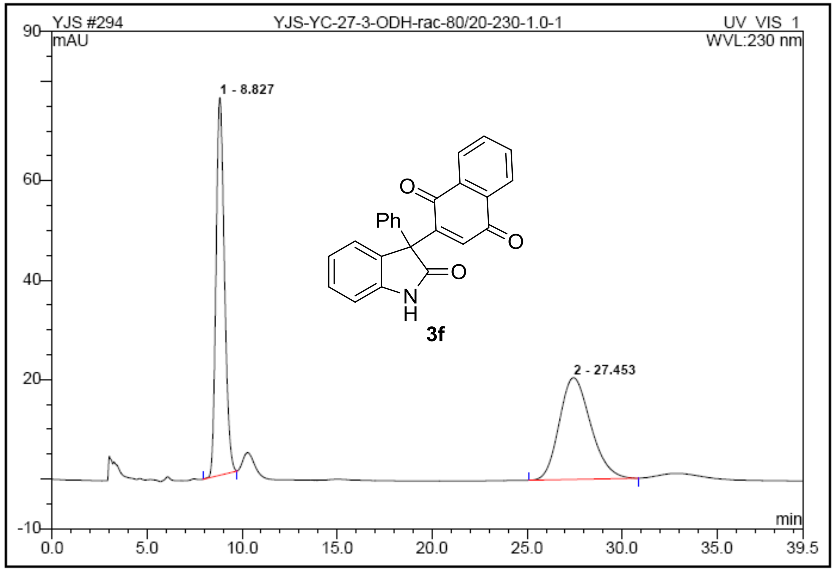

No.	Ret.Time min	Peak Name	Height mAU	Area mAU*min	Rel.Area %	Amount	Type
1	8.83	n.a.	75.941	38.955	49.79	n.a.	BMB
2	27.45	n.a.	20.460	39.280	50.21	n.a.	BMB
**Total:**			96.401	78.235	100.00	0.000	

321 YJS-YC-60-ODH-ASY-80/20-230-1.0-1

Sample Name:	**YJS-YC-60-ODH-ASY-80/20-230-1.0-1**	*Injection Volume:*	**20.0**
Vial Number:	**649**	*Channel:*	**UV_VIS_1**
Sample Type:	**standard**	*Wavelength:*	**230**
Control Program:	**YJS**	*Bandwidth:*	**n.a.**
Quantif. Method:	**YJS**	*Dilution Factor:*	**1.0000**
Recording Time:	**2012-5-12 17:46**	*Sample Weight:*	**1.0000**
Run Time (min):	**37.86**	*Sample Amount:*	**1.0000**

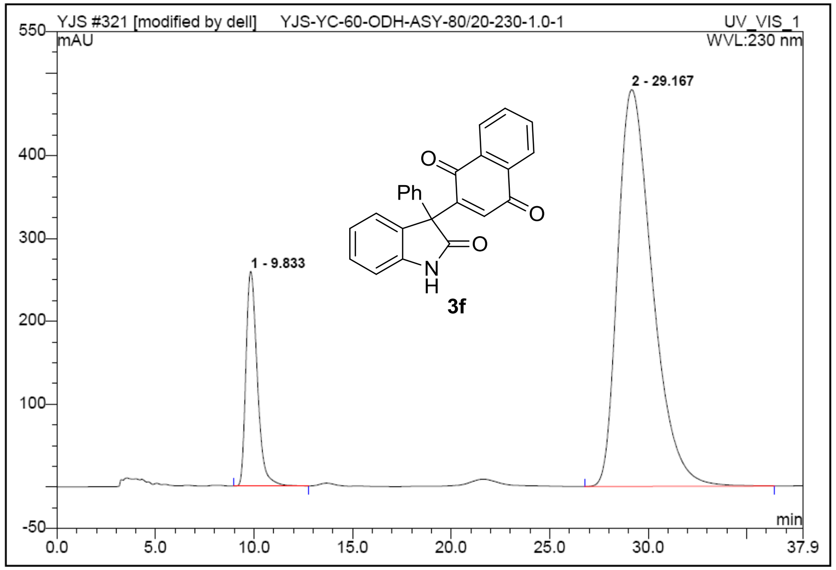

No.	Ret.Time min	Peak Name	Height mAU	Area mAU*min	Rel.Area %	Amount	Type
1	9.83	n.a.	259.155	173.133	15.07	n.a.	BMB
2	29.17	n.a.	479.137	975.796	84.93	n.a.	BMB
**Total:**			738.292	1148.930	100.00	0.000	

Operator:dell Timebase:U-3000 Sequence:YJS

Page
2012-5-20 5:32

332 YJS-YC-43-1-ODH-RAC-80/20-254-1.0-1

Sample Name:	**YJS-YC-43-1-ODH-RAC-80/20-254-1.0-1**	*Injection Volume:*	**20.0**
Vial Number:	**660**	*Channel:*	**UV_VIS_1**
Sample Type:	**standard**	*Wavelength:*	**254**
Control Program:	**YJS**	*Bandwidth:*	**n.a.**
Quantif. Method:	**YJS**	*Dilution Factor:*	**1.0000**
Recording Time:	**2012-5-16 20:14**	*Sample Weight:*	**1.0000**
Run Time (min):	**22.66**	*Sample Amount:*	**1.0000**

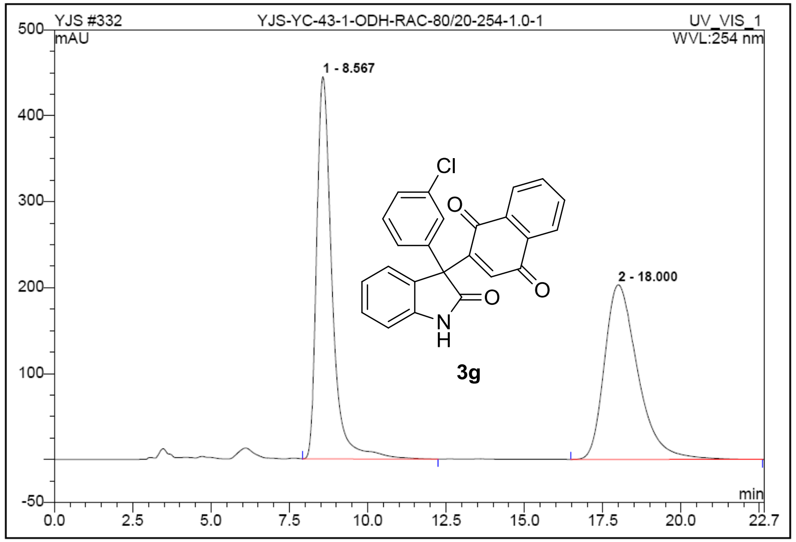

No.	Ret.Time min	Peak Name	Height mAU	Area mAU*min	Rel.Area %	Amount	Type
1	8.57	n.a.	444.818	251.840	50.16	n.a.	BMB
2	18.00	n.a.	203.230	250.268	49.84	n.a.	BMB
**Total:**			648.048	502.109	100.00	0.000	

Operator:dell Timebase:U-3000 Sequence:YJS

Page
2012-5-20 5:29

322 YJS-YC-72-ODH-ASY-80/20-254-1.0-1

Sample Name:	**YJS-YC-72-ODH-ASY-80/20-254-1.0-1**	*Injection Volume:*	**20.0**
Vial Number:	**650**	*Channel:*	**UV_VIS_1**
Sample Type:	**standard**	*Wavelength:*	**254**
Control Program:	**YJS**	*Bandwidth:*	**n.a.**
Quantif. Method:	**YJS**	*Dilution Factor:*	**1.0000**
Recording Time:	**2012-5-12 18:30**	*Sample Weight:*	**1.0000**
Run Time (min):	**26.70**	*Sample Amount:*	**1.0000**

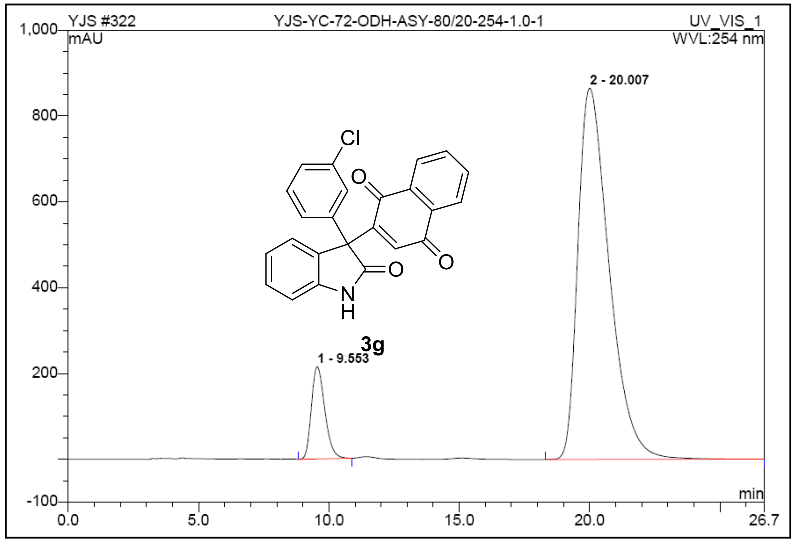

No.	Ret.Time min	Peak Name	Height mAU	Area mAU*min	Rel.Area %	Amount	Type
1	9.55	n.a.	214.667	128.257	9.64	n.a.	BMB
2	20.01	n.a.	865.182	1201.694	90.36	n.a.	BMB
**Total:**			1079.849	1329.952	100.00	0.000	

Operator:dell Timebase:U-3000 Sequence:YJS

Pag
2012-5-20 5:34

209 YJS-YB-133-1-RAC-ODH-80/20-230-1.0-1

Sample Name:	**YJS-YB-133-1-RAC-ODH-80/20-230-1.0-1**	*Injection Volume:*	**20.0**
Vial Number:	**534**	*Channel:*	**UV_VIS_1**
Sample Type:	**standard**	*Wavelength:*	**230**
Control Program:	**YJS**	*Bandwidth:*	**n.a.**
Quantif. Method:	**YJS**	*Dilution Factor:*	**1.0000**
Recording Time:	**2012-3-29 17:18**	*Sample Weight:*	**1.0000**
Run Time (min):	**34.93**	*Sample Amount:*	**1.0000**

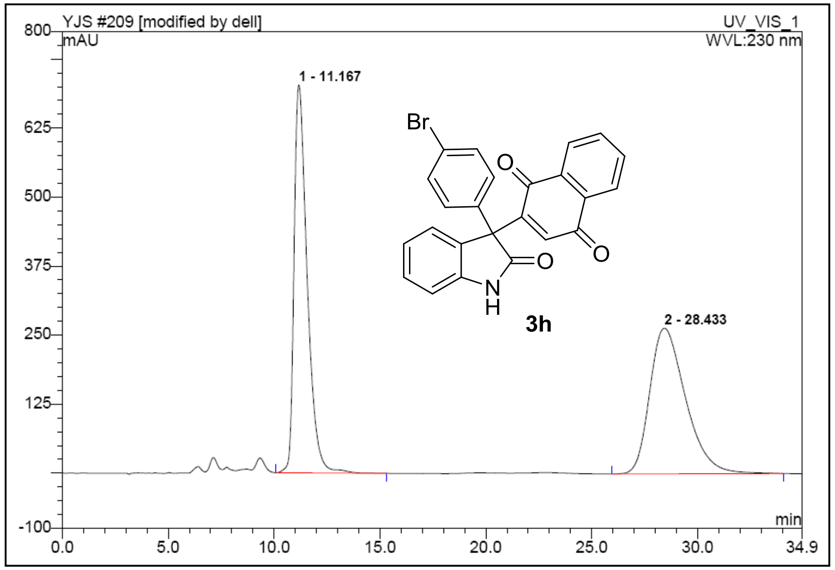

No.	Ret.Time min	Peak Name	Height mAU	Area mAU*min	Rel.Area %	Amount	Type
1	11.17	n.a.	702.526	504.446	49.31	n.a.	BMB
2	28.43	n.a.	263.898	518.543	50.69	n.a.	BMB
**Total:**			966.424	1022.989	100.00	0.000	

Operator:dell Timebase:U-3000 Sequence:YJS

Pag
2012-5-20 5:33

328 YJS-YC-76-ODH-ASY-80/20-230-1.0-1

Sample Name:	**YJS-YC-76-ODH-ASY-80/20-230-1.0-1**	*Injection Volume:*	**20.0**
Vial Number:	**656**	*Channel:*	**UV_VIS_1**
Sample Type:	**standard**	*Wavelength:*	**230**
Control Program:	**YJS**	*Bandwidth:*	**n.a.**
Quantif. Method:	**YJS**	*Dilution Factor:*	**1.0000**
Recording Time:	**2012-5-16 18:27**	*Sample Weight:*	**1.0000**
Run Time (min):	**36.33**	*Sample Amount:*	**1.0000**

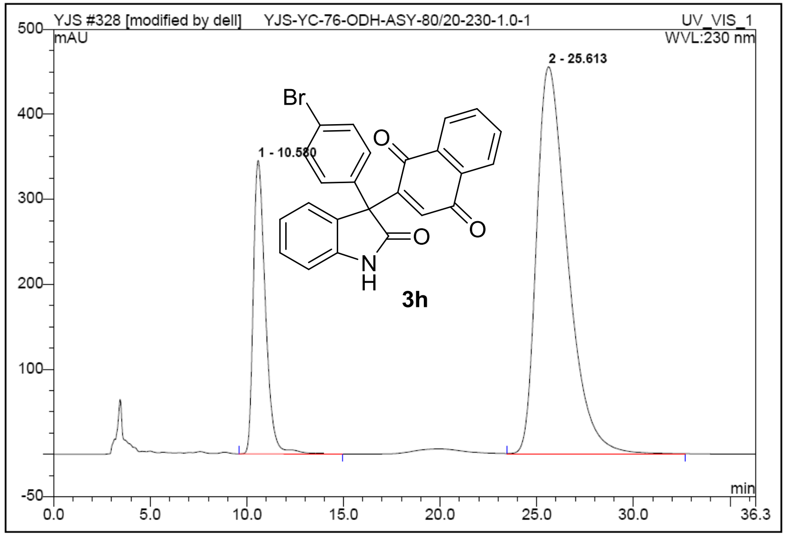

No.	Ret.Time min	Peak Name	Height mAU	Area mAU*min	Rel.Area %	Amount	Type
1	10.58	n.a.	345.204	250.432	22.85	n.a.	BMB
2	25.61	n.a.	455.192	845.496	77.15	n.a.	BMB*
**Total:**			800.395	1095.927	100.00	0.000	

212 YJS-YB-133-4-RAC-ODH-80/20-230-1.0-1

Sample Name:	**YJS-YB-133-4-RAC-ODH-80/20-230-1.0-1**	*Injection Volume:*	**20.0**
Vial Number:	**537**	*Channel:*	**UV_VIS_1**
Sample Type:	**standard**	*Wavelength:*	**230**
Control Program:	**YJS**	*Bandwidth:*	**n.a.**
Quantif. Method:	**YJS**	*Dilution Factor:*	**1.0000**
Recording Time:	**2012-3-29 19:03**	*Sample Weight:*	**1.0000**
Run Time (min):	**21.37**	*Sample Amount:*	**1.0000**

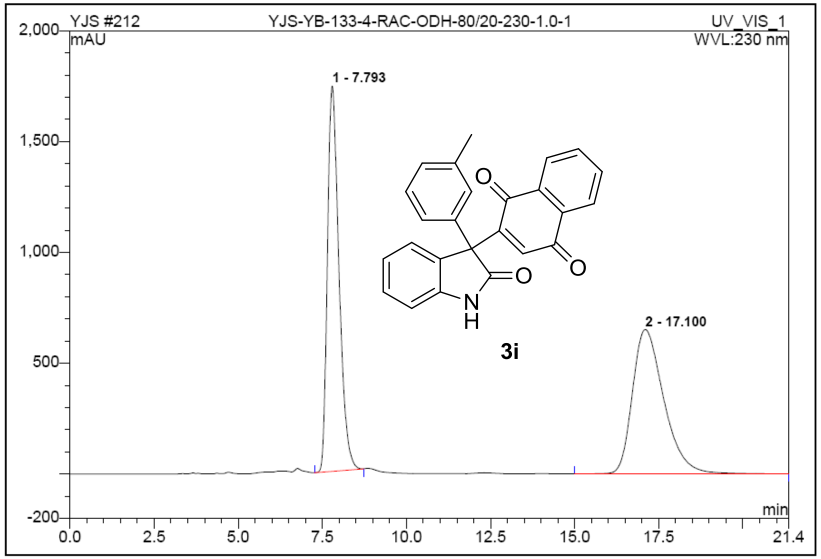

No.	Ret.Time min	Peak Name	Height mAU	Area mAU*min	Rel.Area %	Amount	Type
1	7.79	n.a.	1738.515	691.397	49.47	n.a.	BMB
2	17.10	n.a.	651.589	706.187	50.53	n.a.	BMB
**Total:**			2390.104	1397.584	100.00	0.000	

305 YJS-YC-55-5-ODH-ASY-80/20-230-1.0-1

Sample Name:	**YJS-YC-55-5-ODH-ASY-80/20-230-1.0-1**	*Injection Volume:*	**20.0**
Vial Number:	**633**	*Channel:*	**UV_VIS_1**
Sample Type:	**standard**	*Wavelength:*	**230**
Control Program:	**YJS**	*Bandwidth:*	**n.a.**
Quantif. Method:	**YJS**	*Dilution Factor:*	**1.0000**
Recording Time:	**2012-5-4 21:00**	*Sample Weight:*	**1.0000**
Run Time (min):	**26.26**	*Sample Amount:*	**1.0000**

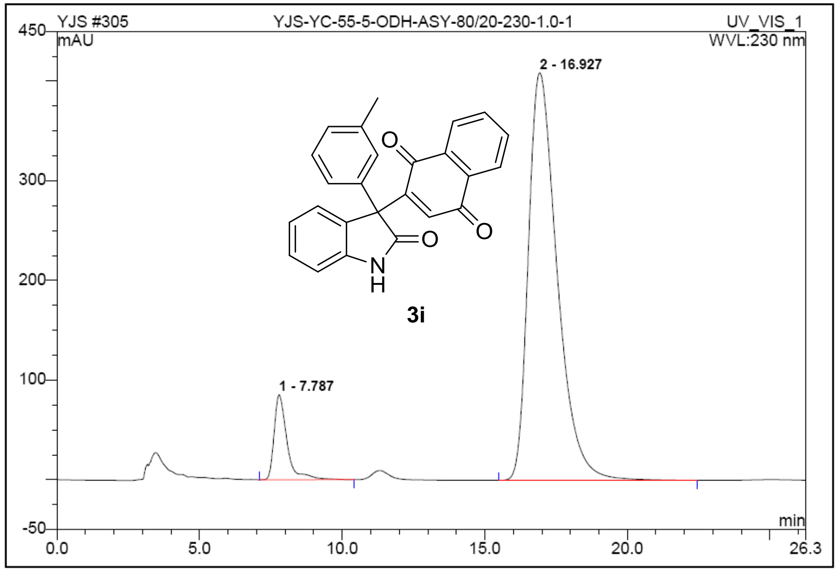

No.	Ret.Time min	Peak Name	Height mAU	Area mAU*min	Rel.Area %	Amount	Type
1	7.79	n.a.	85.315	44.982	8.58	n.a.	BMB
2	16.93	n.a.	409.129	479.318	91.42	n.a.	BMB
**Total:**			494.444	524.300	100.00	0.000	

Operator:dell Timebase:U-3000 Sequence:YJS

Page
2012-5-23 4:25

343 yjs-yC-47-ODH-rac-80/20-254-1.0-1

Sample Name:	**yjs-yC-47-ODH-rac-80/20-254-1.0-1**	*Injection Volume:*	**20.0**
Vial Number:	**672**	*Channel:*	**UV_VIS_1**
Sample Type:	**standard**	*Wavelength:*	**254**
Control Program:	**YJS**	*Bandwidth:*	**n.a.**
Quantif. Method:	**YJS**	*Dilution Factor:*	**1.0000**
Recording Time:	**2012-5-23 15:39**	*Sample Weight:*	**1.0000**
Run Time (min):	**44.36**	*Sample Amount:*	**1.0000**

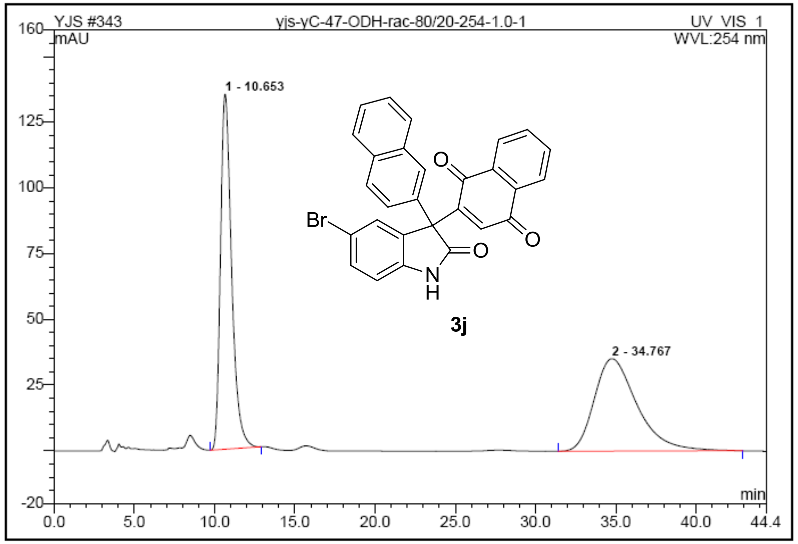

No.	Ret.Time min	Peak Name	Height mAU	Area mAU*min	Rel.Area %	Amount	Type
1	10.65	n.a.	134.990	108.542	49.89	n.a.	BMB
2	34.77	n.a.	35.176	109.001	50.11	n.a.	BMB
**Total:**			170.166	217.543	100.00	0.000	

Operator:dell Timebase:U-3000 Sequence:YJS

Page 1
2012-5-20 5:25

323 YJS-YC-71-ODH-ASY-80/20-254-1.0-1

Sample Name:	**YJS-YC-71-ODH-ASY-80/20-254-1.0-1**	*Injection Volume:*	**20.0**
Vial Number:	**651**	*Channel:*	**UV_VIS_1**
Sample Type:	**standard**	*Wavelength:*	**254**
Control Program:	**YJS**	*Bandwidth:*	**n.a.**
Quantif. Method:	**YJS**	*Dilution Factor:*	**1.0000**
Recording Time:	**2012-5-12 18:58**	*Sample Weight:*	**1.0000**
Run Time (min):	**50.00**	*Sample Amount:*	**1.0000**

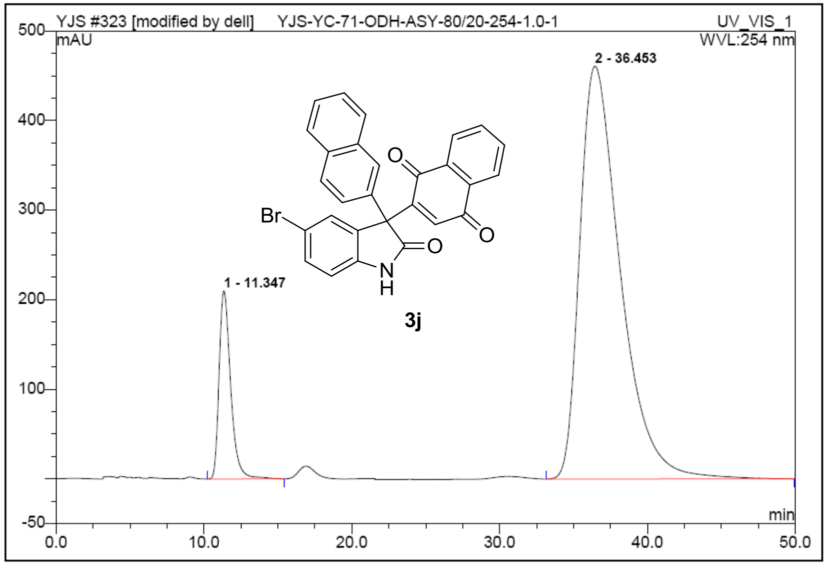

No.	Ret.Time min	Peak Name	Height mAU	Area mAU*min	Rel.Area %	Amount	Type
1	11.35	n.a.	209.865	190.384	11.51	n.a.	BMB
2	36.45	n.a.	460.936	1464.031	88.49	n.a.	BMB
**Total:**			670.801	1654.415	100.00	0.000	

Operator:dell Timebase:U-3000 Sequence:YJS Pag
2012-5-23 3:40

342 yjs-yB-139-4-ODH-rac-80/20-254-1.0-1

Sample Name:	yjs-yB-139-4-ODH-rac-80/20-254-1.0-1	*Injection Volume:*	20.0
Vial Number:	671	*Channel:*	UV_VIS_1
Sample Type:	standard	*Wavelength:*	254
Control Program:	YJS	*Bandwidth:*	n.a.
Quantif. Method:	YJS	*Dilution Factor:*	1.0000
Recording Time:	2012-5-23 15:04	*Sample Weight:*	1.0000
Run Time (min):	33.77	*Sample Amount:*	1.0000

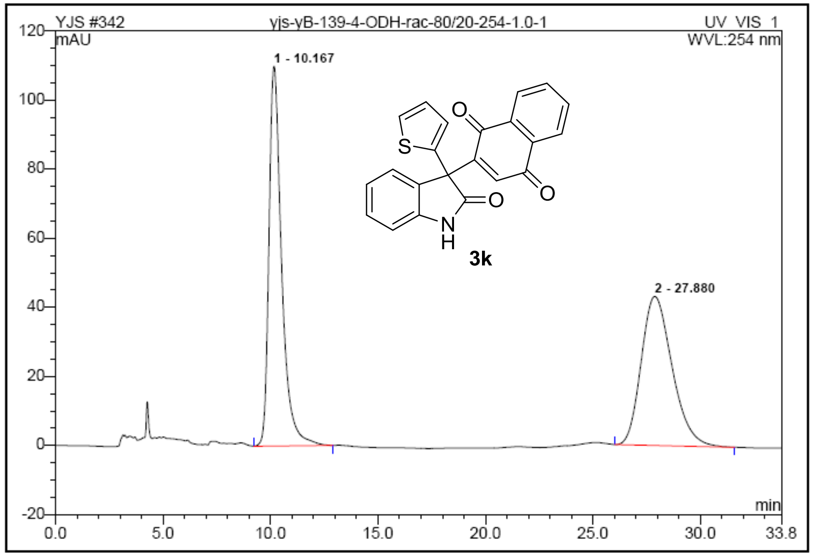

No.	Ret.Time min	Peak Name	Height mAU	Area mAU*min	Rel.Area %	Amount	Type
1	10.17	n.a.	109.987	75.067	51.07	n.a.	BMB
2	27.88	n.a.	43.178	71.918	48.93	n.a.	BMB
Total:			153.165	146.986	100.00	0.000	

Operator:dell Timebase:U-3000 Sequence:YJS Page 1
2012-5-20 7:03

313 YJS-YC-55-3-ODH-ASY-80/20-254-1.0-1

Sample Name:	YJS-YC-55-3-ODH-ASY-80/20-254-1.0-1	*Injection Volume:*	20.0
Vial Number:	641	*Channel:*	UV_VIS_1
Sample Type:	standard	*Wavelength:*	254
Control Program:	YJS	*Bandwidth:*	n.a.
Quantif. Method:	YJS	*Dilution Factor:*	1.0000
Recording Time:	2012-5-9 11:35	*Sample Weight:*	1.0000
Run Time (min):	33.71	*Sample Amount:*	1.0000

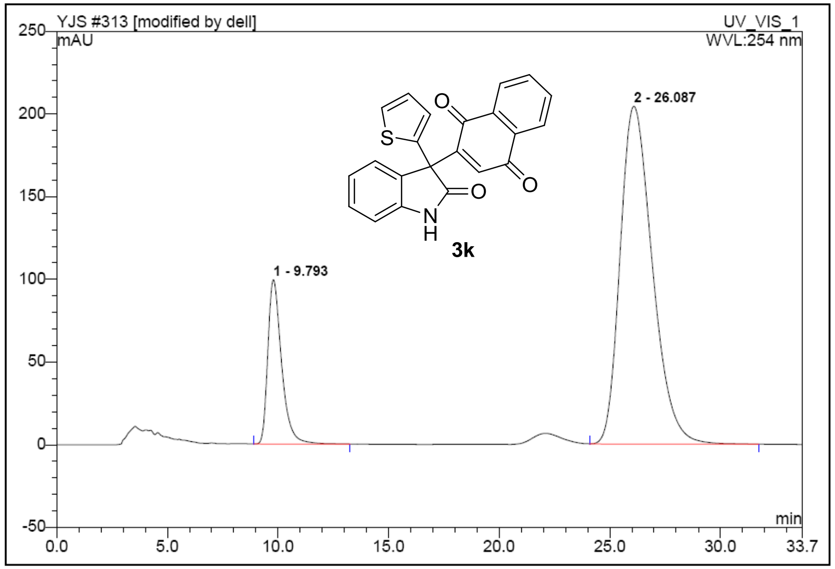

No.	Ret.Time min	Peak Name	Height mAU	Area mAU*min	Rel.Area %	Amount	Type
1	9.79	n.a.	99.394	71.860	17.24	n.a.	BMB
2	26.09	n.a.	204.189	344.946	82.76	n.a.	BMB*
Total:			303.583	416.806	100.00	0.000	

Operator:dell Timebase:U-3000 Sequence:YJS

Pa
2012-5-20 5:3

330 YJS-YB-135-1-ODH-RAC-80/20-254-1.0-1

Sample Name:	**YJS-YB-135-1-ODH-RAC-80/20-254-1.0-1**	*Injection Volume:*	**20.0**
Vial Number:	**658**	*Channel:*	**UV_VIS_1**
Sample Type:	**standard**	*Wavelength:*	**254**
Control Program:	**YJS**	*Bandwidth:*	**n.a.**
Quantif. Method:	**YJS**	*Dilution Factor:*	**1.0000**
Recording Time:	**2012-5-16 19:34**	*Sample Weight:*	**1.0000**
Run Time (min):	**17.63**	*Sample Amount:*	**1.0000**

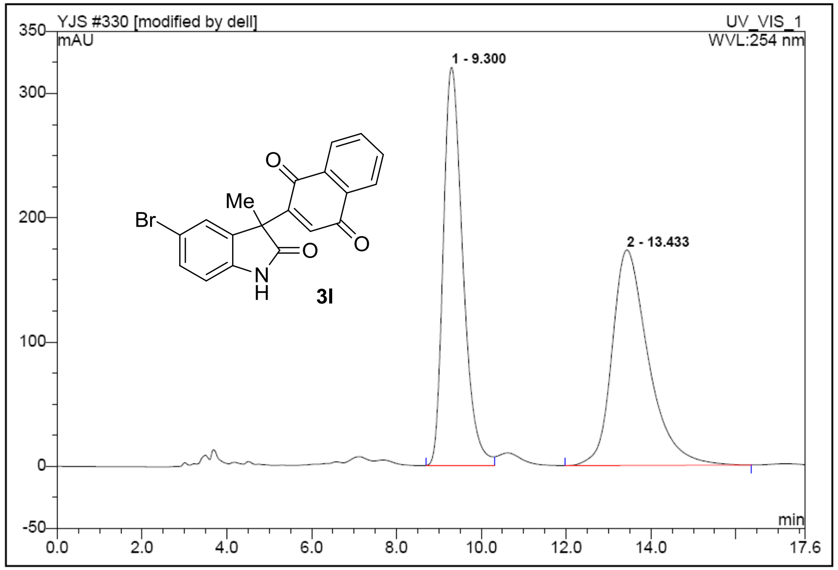

No.	Ret.Time min	Peak Name	Height mAU	Area mAU*min	Rel.Area %	Amount	Type
1	9.30	n.a.	320.300	170.772	49.78	n.a.	BM *
2	13.43	n.a.	173.333	172.288	50.22	n.a.	BMB
Total:			493.633	343.060	100.00	0.000	

Operator:dell Timebase:U-3000 Sequence:YJS

P
2012-5-20 5:

329 YJS-YC-77-ODH-ASY-80/20-254-1.0-1

Sample Name:	**YJS-YC-77-ODH-ASY-80/20-254-1.0-1**	*Injection Volume:*	**20.0**
Vial Number:	**657**	*Channel:*	**UV_VIS_1**
Sample Type:	**standard**	*Wavelength:*	**254**
Control Program:	**YJS**	*Bandwidth:*	**n.a.**
Quantif. Method:	**YJS**	*Dilution Factor:*	**1.0000**
Recording Time:	**2012-5-16 19:15**	*Sample Weight:*	**1.0000**
Run Time (min):	**18.90**	*Sample Amount:*	**1.0000**

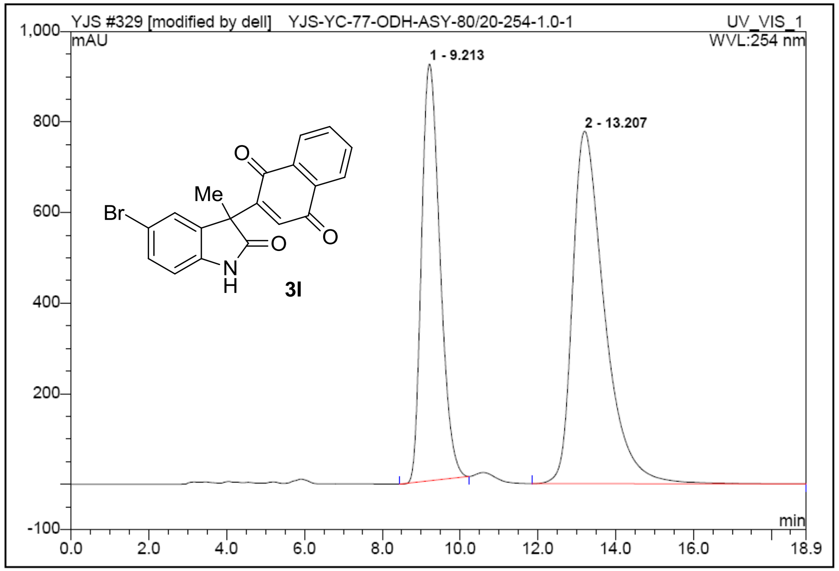

No.	Ret.Time min	Peak Name	Height mAU	Area mAU*min	Rel.Area %	Amount	Type
1	9.21	n.a.	920.491	505.019	40.54	n.a.	BMB*
2	13.21	n.a.	778.373	740.628	59.46	n.a.	BMB*
Total:			1698.864	1245.647	100.00	0.000	

Operator:dell Timebase:U-3000 Sequence:YJS

2012-5-26 6:[illegible]

348 yjs-yc-87-OZH-RAC-90/10-230-1.0-1

Sample Name:	**yjs-yc-87-OZH-RAC-90/10-230-1.0-1**	*Injection Volume:*	**20.0**
Vial Number:	**677**	*Channel:*	**UV_VIS_1**
Sample Type:	**standard**	*Wavelength:*	**230**
Control Program:	**YJS**	*Bandwidth:*	**n.a.**
Quantif. Method:	**YJS**	*Dilution Factor:*	**1.0000**
Recording Time:	**2012-5-26 17:29**	*Sample Weight:*	**1.0000**
Run Time (min):	**35.15**	*Sample Amount:*	**1.0000**

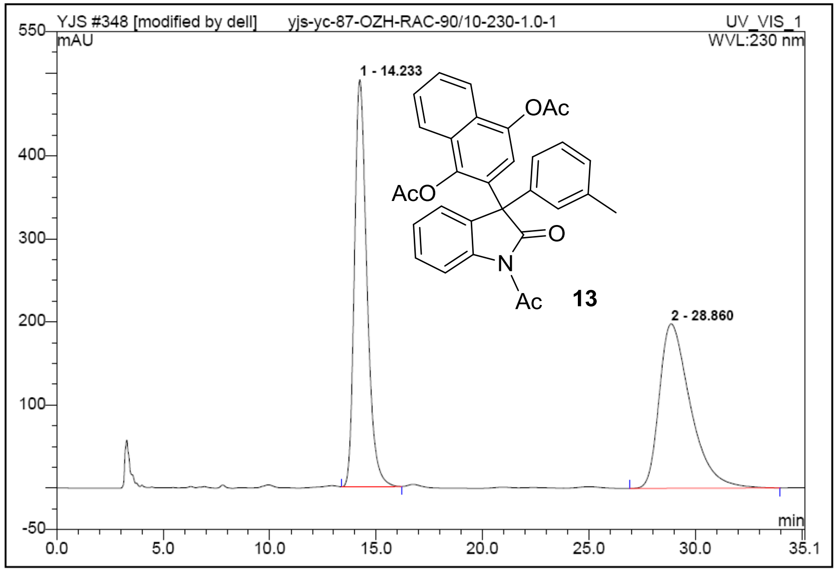

No.	Ret.Time min	Peak Name	Height mAU	Area mAU*min	Rel.Area %	Amount	Type
1	14.23	n.a.	490.423	332.720	49.78	n.a.	BMB
2	28.86	n.a.	198.103	335.603	50.22	n.a.	BMB
**Total:**			688.526	668.323	100.00	0.000	

Operator:dell Timebase:U-3000 Sequence:YJS

2012-5-26 4:[illegible]

346 yjs-yc-92-OZH-asy-90/10-230-1.0-1

Sample Name:	**yjs-yc-92-OZH-asy-90/10-230-1.0-1**	*Injection Volume:*	**20.0**
Vial Number:	**675**	*Channel:*	**UV_VIS_1**
Sample Type:	**standard**	*Wavelength:*	**230**
Control Program:	**YJS**	*Bandwidth:*	**n.a.**
Quantif. Method:	**YJS**	*Dilution Factor:*	**1.0000**
Recording Time:	**2012-5-26 16:14**	*Sample Weight:*	**1.0000**
Run Time (min):	**37.45**	*Sample Amount:*	**1.0000**

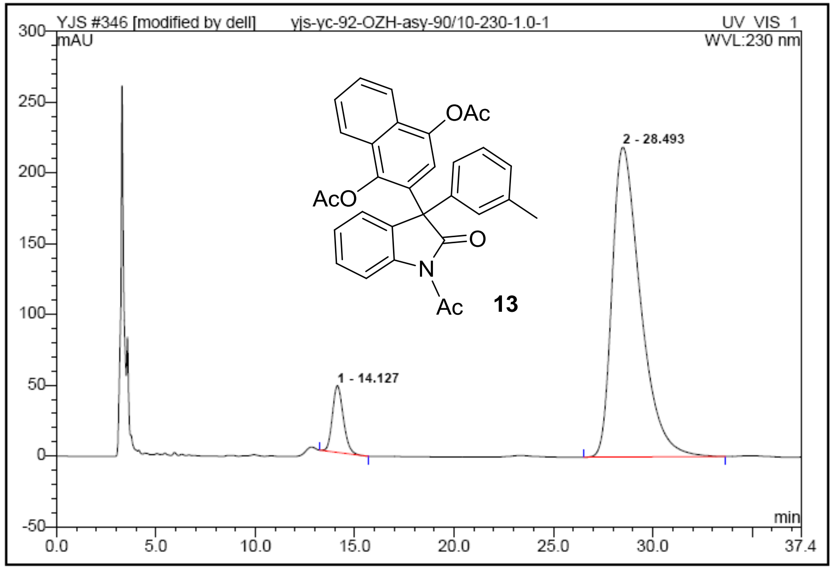

No.	Ret.Time min	Peak Name	Height mAU	Area mAU*min	Rel.Area %	Amount	Type
1	14.13	n.a.	47.106	29.798	7.62	n.a.	BMB*
2	28.49	n.a.	218.913	361.453	92.38	n.a.	BMB
**Total:**			266.018	391.252	100.00	0.000	

Operator:dell Timebase:U-3000 Sequence:YJS

2012-5-26 6:[illegible]

348 yjs-yc-87-OZH-RAC-90/10-230-1.0-1

Sample Name:	**yjs-yc-87-OZH-RAC-90/10-230-1.0-1**	*Injection Volume:*	**20.0**
Vial Number:	**677**	*Channel:*	**UV_VIS_1**
Sample Type:	**standard**	*Wavelength:*	**230**
Control Program:	**YJS**	*Bandwidth:*	**n.a.**
Quantif. Method:	**YJS**	*Dilution Factor:*	**1.0000**
Recording Time:	**2012-5-26 17:29**	*Sample Weight:*	**1.0000**
Run Time (min):	**35.15**	*Sample Amount:*	**1.0000**

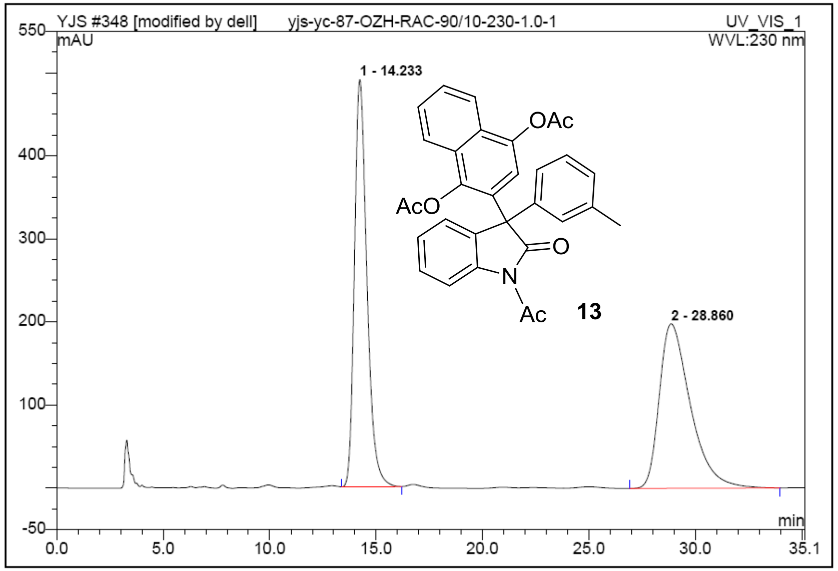

No.	Ret.Time min	Peak Name	Height mAU	Area mAU*min	Rel.Area %	Amount	Type
1	14.23	n.a.	490.423	332.720	49.78	n.a.	BMB
2	28.86	n.a.	198.103	335.603	50.22	n.a.	BMB
**Total:**			688.526	668.323	100.00	0.000	

Operator:dell Timebase:U-3000 Sequence:YJS

2012-5-26 5:32

347 yjs-yc-95-OZH-asy-90/10-230-1.0-1

Sample Name:	**yjs-yc-95-OZH-asy-90/10-230-1.0-1**	*Injection Volume:*	**20.0**
Vial Number:	**676**	*Channel:*	**UV_VIS_1**
Sample Type:	**standard**	*Wavelength:*	**230**
Control Program:	**YJS**	*Bandwidth:*	**n.a.**
Quantif. Method:	**YJS**	*Dilution Factor:*	**1.0000**
Recording Time:	**2012-5-26 16:52**	*Sample Weight:*	**1.0000**
Run Time (min):	**36.45**	*Sample Amount:*	**1.0000**

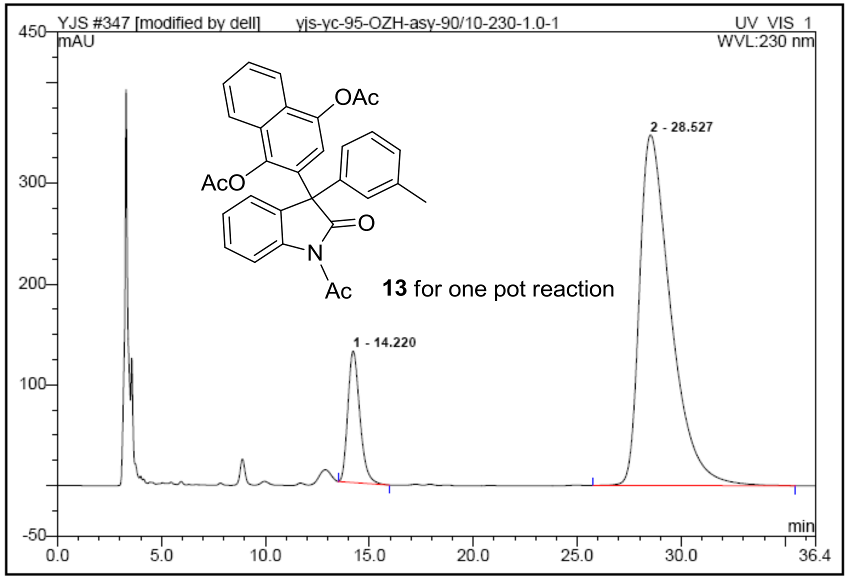

No.	Ret.Time min	Peak Name	Height mAU	Area mAU*min	Rel.Area %	Amount	Type
1	14.22	n.a.	130.305	87.144	12.56	n.a.	BMB
2	28.53	n.a.	348.043	606.463	87.44	n.a.	BMB
**Total:**			478.348	693.607	100.00	0.000	

Operator:dell Timebase:U-3000 Sequence:YJS

Pa
2012-7-10 9:5

349 yjs-yc-134-RAC-80/20-254-1.0-1

Sample Name:	**yjs-yc-134-RAC-80/20-254-1.0-1**	*Injection Volume:*	**20.0**
Vial Number:	**678**	*Channel:*	**UV_VIS_1**
Sample Type:	**standard**	*Wavelength:*	**254**
Control Program:	**YJS**	*Bandwidth:*	**n.a.**
Quantif. Method:	**YJS**	*Dilution Factor:*	**1.0000**
Recording Time:	**2012-7-3 9:22**	*Sample Weight:*	**1.0000**
Run Time (min):	**13.83**	*Sample Amount:*	**1.0000**

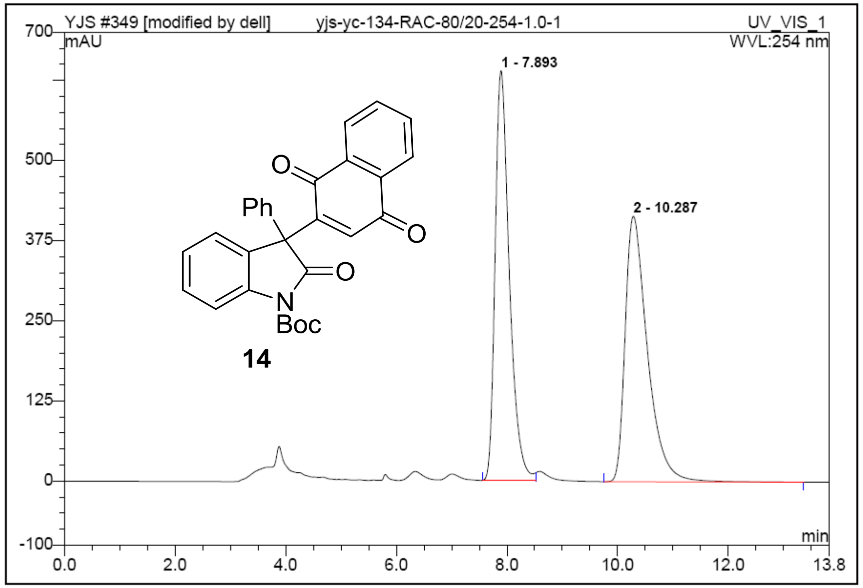

No.	Ret.Time min	Peak Name	Height mAU	Area mAU*min	Rel.Area %	Amount	Type
1	7.89	n.a.	638.883	190.198	49.64	n.a.	BM *
2	10.29	n.a.	413.984	192.940	50.36	n.a.	BMB
**Total:**			1052.867	383.138	100.00	0.000	

Operator:dell Timebase:U-3000 Sequence:YJS

P
2012-7-10 9:

377 yjs-yc-144-IC-asy-80/20-254-1.0-1

Sample Name:	**yjs-yc-144-IC-asy-80/20-254-1.0-1**	*Injection Volume:*	**20.0**
Vial Number:	**708**	*Channel:*	**UV_VIS_1**
Sample Type:	**standard**	*Wavelength:*	**254**
Control Program:	**YJS**	*Bandwidth:*	**n.a.**
Quantif. Method:	**YJS**	*Dilution Factor:*	**1.0000**
Recording Time:	**2012-7-9 22:35**	*Sample Weight:*	**1.0000**
Run Time (min):	**14.51**	*Sample Amount:*	**1.0000**

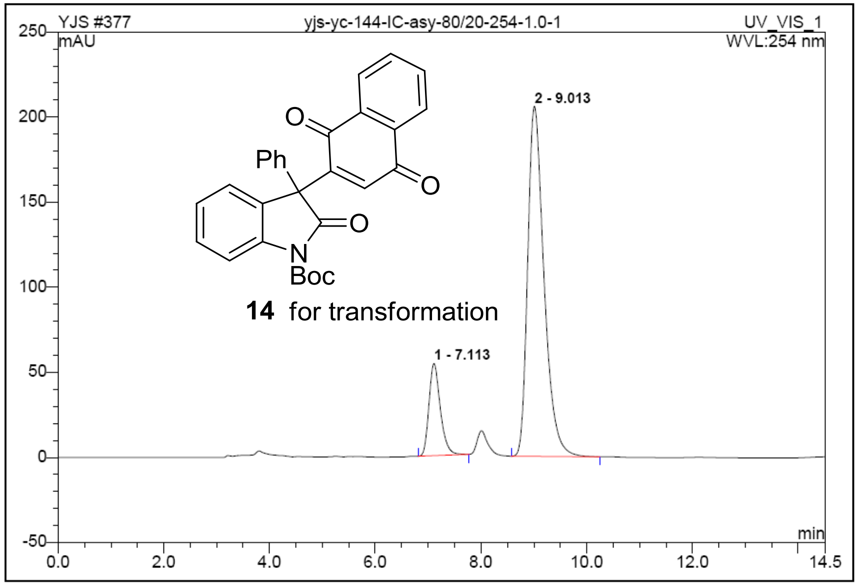

No.	Ret.Time min	Peak Name	Height mAU	Area mAU*min	Rel.Area %	Amount	Type
1	7.11	n.a.	54.076	13.494	15.50	n.a.	BMB
2	9.01	n.a.	205.704	73.568	84.50	n.a.	BMB
**Total:**			259.780	87.062	100.00	0.000	

Operator:dell Timebase:U-3000 Sequence:YJS
2012-7-10 9:[illegible]

349 yjs-yc-134-RAC-80/20-254-1.0-1

Sample Name:	**yjs-yc-134-RAC-80/20-254-1.0-1**	*Injection Volume:*	**20.0**
Vial Number:	**678**	*Channel:*	**UV_VIS_1**
Sample Type:	**standard**	*Wavelength:*	**254**
Control Program:	**YJS**	*Bandwidth:*	**n.a.**
Quantif. Method:	**YJS**	*Dilution Factor:*	**1.0000**
Recording Time:	**2012-7-3 9:22**	*Sample Weight:*	**1.0000**
Run Time (min):	**13.83**	*Sample Amount:*	**1.0000**

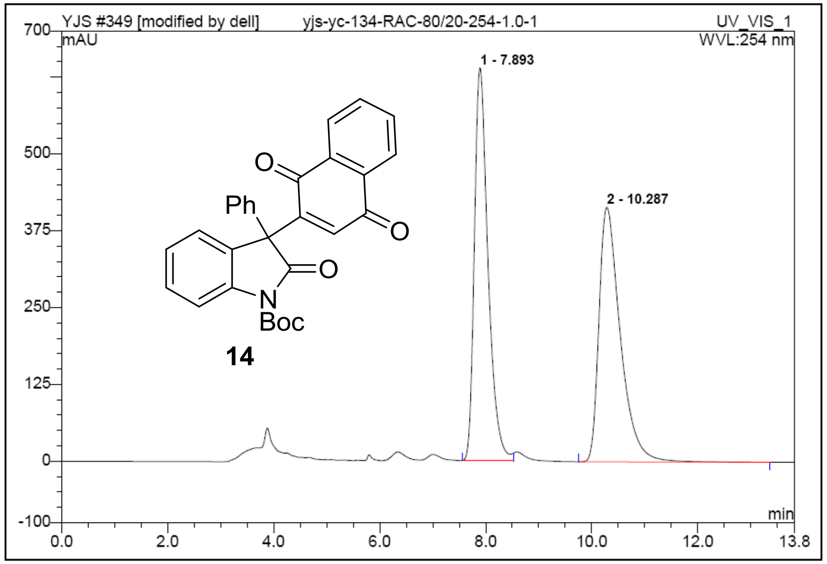

No.	Ret.Time min	Peak Name	Height mAU	Area mAU*min	Rel.Area %	Amount	Type
1	7.89	n.a.	638.883	190.198	49.64	n.a.	BM *
2	10.29	n.a.	413.984	192.940	50.36	n.a.	BMB
**Total:**			1052.867	383.138	100.00	0.000	

Operator:dell Timebase:U-3000 Sequence:YJS
2012-7-10 9[illegible]

350 yjs-yc-132-IC-asy-80/20-254-1.0-1

Sample Name:	**yjs-yc-132-IC-asy-80/20-254-1.0-1**	*Injection Volume:*	**20.0**
Vial Number:	**679**	*Channel:*	**UV_VIS_1**
Sample Type:	**standard**	*Wavelength:*	**254**
Control Program:	**YJS**	*Bandwidth:*	**n.a.**
Quantif. Method:	**YJS**	*Dilution Factor:*	**1.0000**
Recording Time:	**2012-7-3 9:39**	*Sample Weight:*	**1.0000**
Run Time (min):	**15.73**	*Sample Amount:*	**1.0000**

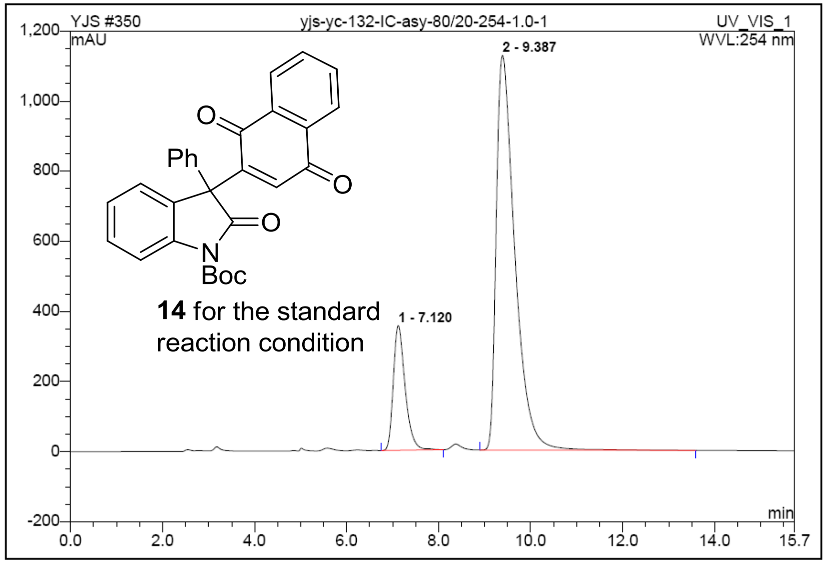

No.	Ret.Time min	Peak Name	Height mAU	Area mAU*min	Rel.Area %	Amount	Type
1	7.12	n.a.	355.559	105.542	16.70	n.a.	BMB
2	9.39	n.a.	1125.482	526.379	83.30	n.a.	BMB
**Total:**			1481.042	631.921	100.00	0.000	
